# Quorum-Sensing Synchronization of Synthetic Toggle Switches: A Design Based on Monotone Dynamical Systems Theory

**DOI:** 10.1371/journal.pcbi.1004881

**Published:** 2016-04-29

**Authors:** Evgeni V. Nikolaev, Eduardo D. Sontag

**Affiliations:** Department of Mathematics and Center for Quantitative Biology, Rutgers, The State University of New Jersey, Piscataway, New Jersy, United States of America; ETH Zürich, SWITZERLAND

## Abstract

Synthetic constructs in biotechnology, biocomputing, and modern gene therapy interventions are often based on plasmids or transfected circuits which implement some form of “on-off” switch. For example, the expression of a protein used for therapeutic purposes might be triggered by the recognition of a specific combination of inducers (e.g., antigens), and memory of this event should be maintained across a cell population until a specific stimulus commands a coordinated shut-off. The robustness of such a design is hampered by molecular (“intrinsic”) or environmental (“extrinsic”) noise, which may lead to spontaneous changes of state in a subset of the population and is reflected in the bimodality of protein expression, as measured for example using flow cytometry. In this context, a “majority-vote” correction circuit, which brings deviant cells back into the required state, is highly desirable, and quorum-sensing has been suggested as a way for cells to broadcast their states to the population as a whole so as to facilitate consensus. In this paper, we propose what we believe is the first such a design that has mathematically guaranteed properties of stability and auto-correction under certain conditions. Our approach is guided by concepts and theory from the field of “monotone” dynamical systems developed by M. Hirsch, H. Smith, and others. We benchmark our design by comparing it to an existing design which has been the subject of experimental and theoretical studies, illustrating its superiority in stability and self-correction of synchronization errors. Our stability analysis, based on dynamical systems theory, guarantees global convergence to steady states, ruling out unpredictable (“chaotic”) behaviors and even sustained oscillations in the limit of convergence. These results are valid no matter what are the values of parameters, and are based only on the wiring diagram. The theory is complemented by extensive computational bifurcation analysis, performed for a biochemically-detailed and biologically-relevant model that we developed. Another novel feature of our approach is that our theorems on exponential stability of steady states for homogeneous or mixed populations are valid independently of the number *N* of cells in the population, which is usually very large (*N* ≫ 1) and unknown. We prove that the exponential stability depends on relative proportions of each type of state only. While monotone systems theory has been used previously for systems biology analysis, the current work illustrates its power for synthetic biology design, and thus has wider significance well beyond the application to the important problem of coordination of toggle switches.

## Author Summary

For the last decade, outstanding progress has been made, and considerable practical experience has accumulated, in the construction of elementary genetic circuits that perform various tasks, such as memory storage and logical operations, in response to both exogenous and endogenous stimuli. Using modern molecular “plug-and-play” technologies, various (re-)programmable cellular populations can be engineered, and they can be combined into more complex cellular systems. Among all engineered synthetic circuits, a *toggle*, a robust bistable switch leading to a binary response dynamics, is the simplest basic synthetic biology device, analogous to the “flip-flop” or latch in electronic design, and it plays a key role in biotechnology, biocomputing, and proposed gene therapies. However, despite many remarkable properties of the existing toggle designs, they must be tightly controlled in order to avoid spontaneous switching between different expression states (loss of long-term memory) or even the breakdown of stability through the generation of stable oscillations. To address this concrete challenge, we have developed a new design for quorum-sensing synthetic toggles, based on monotone dynamical systems theory. Our design is endowed with strong theoretical guarantees that completely exclude unpredictable chaotic behaviors in the limit of convergence, as well as undesired stable oscillations, and leads to robust consensus states.

## Introduction

In the short period since the pioneering milestones in synthetic biology [1, 2], outstanding progress has been made, and considerable practical experience has accumulated, in the construction of genetic circuits that perform various tasks, such as memory storage and logical operations, as well as support biomedical interventions and biotechnological manipulations in response to both exogenous and endogenous stimuli [3–9]. These circuits often include plasmids or transfected circuits which implement some form of “on-off” binary device, generically referred to as a *toggle switch*. For example, the expression of a protein used for gene therapy could be triggered by the recognition of some combination of inducers such as antigens, and memory of this event should be maintained across a cell population until a specific stimulus commands a coordinated shut-off [1, 3, 4]. In this context, as well as in many others, it is desirable for populations of cells to achieve coordinated static and/or dynamic functionalities. However, this coordination is hampered by molecular (“intrinsic”) or environmental (“extrinsic”) noise, which may lead to spontaneous changes of state in a subset of the population and is reflected in the bimodality of protein expression, as measured for example using flow cytometry.

To achieve robustness across a population, one may implement a “majority-vote” correction circuit that brings deviant cells back into the desired state. Much synthetic biology research focuses on single-cell microorganisms, often bacteria [4, 6–9]. Bacterial populations are relatively simple, and some aspects of their complex sociality can be rationally understood [10], providing a foundation for building more complex cellular systems. For bacteria, quorum-sensing (QS) has been suggested as a way for cells to broadcast their states to the population as a whole so as to facilitate consensus. QS signaling pathways [11] can, for example, regulate bacterial gene expression in response to fluctuations in cell-population density. Bacteria produce and release various signaling molecules called autoinducers (AIs) [11–14]. The detection of a minimal threshold stimulatory concentration of an AI leads to an alteration in the host’s gene expression. Both Gram-positive and Gram-negative bacteria use QS communication to regulate a diverse array of physiological activities. Synthetic biology design has adopted QS communication in its toolbox [15], because natural and artificially engineered QS modules can be used to interface synthetic circuits with exogenous and endogenous cues [4], and a systematic modular approach to standardize engineering toggle genetic circuits that would allow programmed cells to be designed for various specific purposes and to communicate their states to other cells was suggested as a bioengineering “plug-and-play” modular approach [4]. The design of such QS-toggle combinations is the focus of this paper.

### A Known Design and Its Drawbacks

We benchmark our design by comparing it to Toggle B2 [4], see Fig SI-1.1 in S1 Text. Despite remarkable properties of design B2, observed experimentally in controllable experimental settings [4], and studied theoretically [16, 17], the fact that their functional repertoire includes not only a bistable long-term memory but also the generation of stable oscillations suggests that the environment-toggle system must be tightly controlled in order to avoid spontaneous switching, not merely between different expression states but even between different functions.

To address this challenge, we propose a novel design, which is endowed with mathematically guaranteed properties of stability and auto-correction. Our approach is closely guided by concepts and theory from the powerful framework of *monotone dynamical systems* pioneered by M. Hirsch and H. Smith [18–23].

We employ monotone theory to provide guarantees of global convergence to steady states, thus ruling out unpredictable (“chaotic”) behaviors and sustained oscillations. These theorems are valid no matter for all values of parameters and are based only on the network structure. We also provide an extensive computational bifurcation analysis of the corresponding biochemically-detailed and biologically-relevant mathematical models. Our results for homogeneous or mixed populations are valid independently of the number of cells in the population (*N* ≫ 1), and depend only on the relative proportions of each type of state.

### The Components

As a basic design, we chose a genetic toggle switch consisting of two mutually repressing genes, *lacI* and *tetR* [1]. We use two acylated homoserine lactones (Acyl-HSLs), (*i*) *N*-butanoyl-l-homoserine lactone (C4-HSL) secreted by *Pseudomonas aeruginosa* [24], and (*ii*) *N*-(3-hydroxy-7-*cis*-tetradecenoyl)-L-homoserine lactone (3-OH-C14-HSL) produced by *Rhizobium leguminosarum* [13] as a means of coordinating toggle-host activity. Our design has two QS arms built-in the toggle in such a way that each promoter-repressor pair is controlled by its own QS signaling pathway symmetrically. Because of this “mirror-like” toggle symmetry, we call our design a symmetric toggle or an “S” design.

To benchmark the new S toggle design and the monotone systems approach, we compare the S design to the well-studied asymmetric B2-strain (Fig SI-1.1 in S1 Text) which has one QS arm only [4, 16]. In this work, we call the asymmetric B2-strain the “A” design. Our S design cannot be reduced to the A design by removing one QS arm, and, thus, the S design cannot be viewed as a straightforward extension of the A design. From a theoretical standpoint, it is worth remarking that the A design is non-monotone.

Under certain experimentally controllable conditions (*e.g*, unsaturated levels of AAA+ proteases ClpXP, *etc.*), the S vs. A toggle comparative results obtained in this work can be summarized as follows:

- The S toggle design completely excludes any unpredictable chaotic behaviors, as well as stable oscillations. Typical trajectories converge globally to stable equilibria. This conclusion is valid for all parameter values, and provides a strong theoretical guarantee missing from other synthetic biology designs.
- We refer to mixed states leading to bimodal distributions as *spontaneous synchronization errors*. We find that the S toggle design is able to self-correct (or, auto-correct) synchronization errors, while the non-monotone A toggle design is not.
- We show how monotone systems theory can predict not only the dynamics of an S toggle population but it also explains certain monotonically increasing or decreasing parametric dependencies of population steady states. Some of these predictions can facilitate self-synchronization and, thereby, reduce the chance for synchronization errors to emerge spontaneously.

### Organization of Paper

In Models, the S toggle and A toggle mathematical models are introduced. The basic formalism and fundamental mathematical results of monotone systems theory, including Strong Monotonicity and Hirsch’s Theorem [18–21, 23] are as well reviewed. Reference values of dimensionless parameters, scaling, and selection and interpretation of bifurcation parameters are discussed. We also formalize a concept of spontaneous synchronization errors by considering three types of equilibrium populations: One homogeneous population, and two heterogeneous (mixed) populations (bimodal distributions) with both equally (1:1)-mixed and not-equally (*N*_1_:*N*_2_)-mixed transcriptional signatures, *N*_2_ ≪ *N*_1_, the latter giving rise to spontaneous synchronization errors, where *N* = *N*_1_ + *N*_2_, and *N* is the number of cells in the given population.

In Results and Discussion, we proceed to a comparative theoretical and computational analysis of the S and A toggle designs. We begin this section with results on the application of monotone systems theory to the S design, as these results constitute the main conceptual and practical subject motivating this work (Application of Monotone Systems Theory to the S Design). We then explain how monotone systems theory allows one to predict, based on qualitative knowledge only, that generically all solutions converge to equilibria, with no possible oscillations [16] nor chaotic behavior [25], no matter what the kinetic parameters are. This is in contrast to the A design, which may admit oscillations [16]. Next, we analyze single S and A toggles decoupled from the environment (Bistability in Single S and A Toggles), and observe that the S toggle remains bistable even if “redundant” repressor genes are removed from the corresponding plasmids. To show how the S design is more robust than the A design, we carry out a comparative bifurcation analysis of populations consisting of coupled S or A toggles. We select a free (bifurcation) dimensionless parameter which can be interpreted in terms of experimental interventions [6] leading to (*a*) changes in the membrane permeability, or (*b*) changes in the half-lives of repressor proteins, or (*c*) changes in the specific growth rate of the host cell. We finally test the toggle design capabilities to self-correct spontaneous synchronization errors by sampling the basin of attractor of the corresponding equilibrium solutions. We find that the S toggle design is able to self-correct synchronization errors far better than the A toggle design.

The paper also has Supplemental Information (SI) materials S1–S8 which can be found in S1 text (the file S1-text.pdf).

In SI-1 Toggle B2 (see S1 Text), we discuss the relationship between A design and its prototype, the *E. coli* strain “B2” developed by Kobayashi et al [4] who considered a number of genetic toggle switches, interfaced with a QS signaling pathway.

In SI-2 Model Derivation (see S1 Text), we derive mathematical models and carry out a nondimensionalization procedure, the conclusions of which are used in the main text (Scaling).

In SI-3 Estimation of Parameter Values (see S1 Text), we discuss ranges of biologically meaningful parameter values based on data available in the existing literature. Values of biologically meaningful parameters depend upon experimental conditions and other factors controlled by an experimenter, as reviewed in [6]. Therefore, we provide an example of a concrete estimation of the values of dimensionless parameters, which we interpret in terms of interventions reviewed in [6].

In SI-4 Alternative Definitions of Monotone Systems and Order Preservation (see S1 Text), balanced graphs, relation to graph partitions, and order presentation by flows are explained.

In SI-5 Symmetry (see S1 Text), we formalize symmetry of the S design and discuss interpretation of symmetric results with respect to nonsymmetric perturbations typical for experimental systems.

In SI-6 Exponential Stability of Cellular Populations (see S1 Text), we prove a number of general theorems to analyze exponential stability [26] of both homogeneous and heterogeneous (mixed) population equilibrium states, independently of the number *N* of cells in the given population, which (*i.e.,* the value of *N* ≥ 2) can be *a priori* unknown.

In SI-7 Additional Figures (see S1 Text), we provide additional bifurcation diagrams.

In SI-8 Modification of the S and A Models to Describe Sequestration of AAA+ protease ClpXP (see S1 Text), additional (modified) mathematical models describing competition of ssrA-tagged protein molecules for AAA+ proteases ClpXP are described.

## Models

Although our main objective in this paper is to present a conceptual and general organizing principle for the construction of self-correcting “majority-vote” multistable synthetic systems, we instantiate our ideas through a very concrete set of genes and protein products, all being standard molecular parts in synthetic biology [1, 2, 4, 7–9, 27–31]. We do that in order to emphasize the fact that our constructs can be realistically implemented with currently available molecular components. However, replacing these components with others does not change the basic mathematical framework.

To facilitate a conceptual and quantitative comparison of the S and A toggle designs, the corresponding genetic circuits are assumed to be built from the same tightly controlled *lac-tet* transcription entities [7–9, 32–37], which have been intensively used in a number of experimental and theoretic-modeling studies in the context of synthetic biology [1, 2, 4, 7–9, 28–31]. Below, we briefly characterize relevant molecular details and then form the corresponding mathematical models.

### Toggle Designs

**Fig 1:**
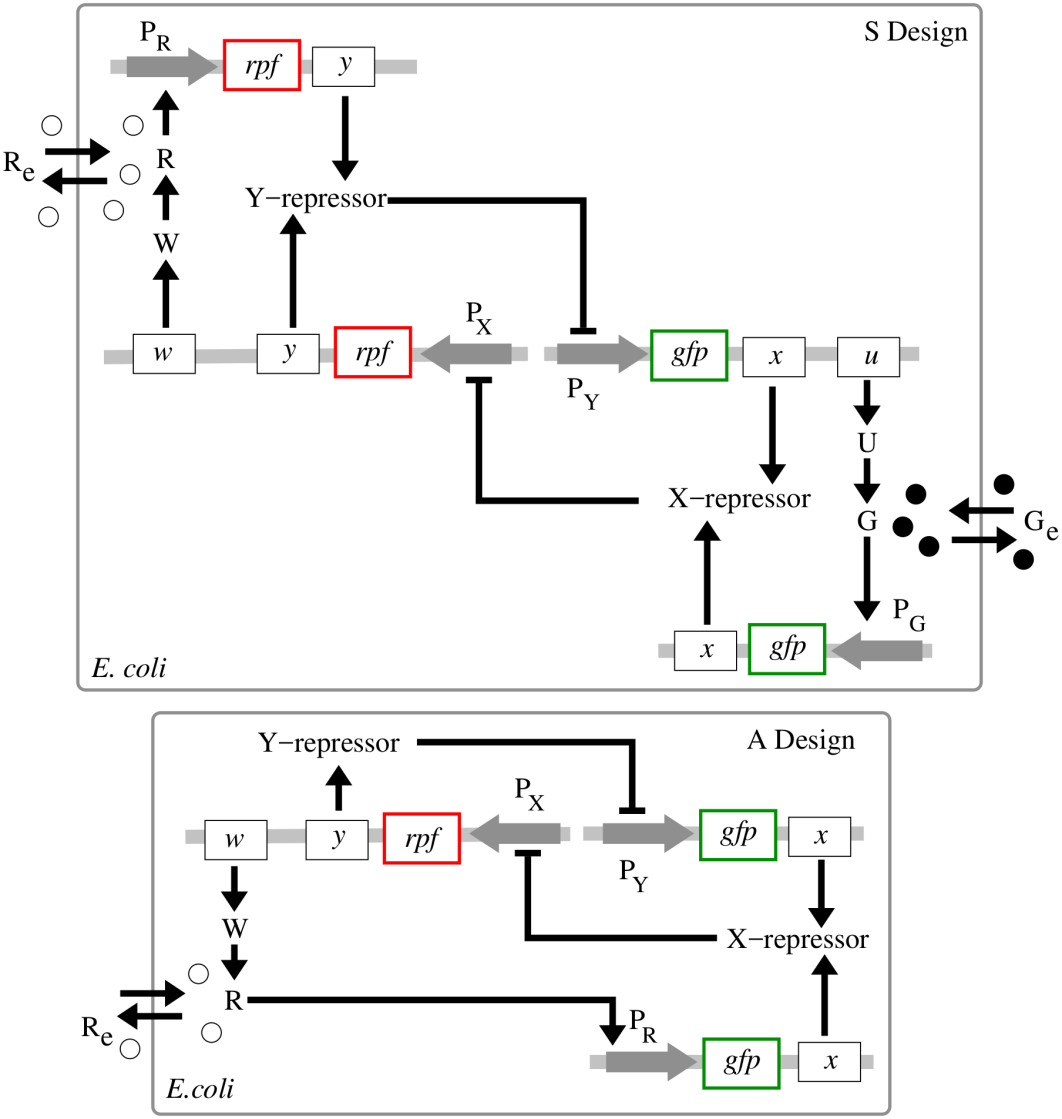
Monotone-symmetric and nonmonotone-asymmetric toggle designs. **S design** (*top panel*): Activation of the expression of gene *x* (*lacI*) occurs by binding of autoinducer G (C14-HSL) to promoter P_G_ (P_cin_). Inhibition of the expression of both genes *x* (*lacI*) and *u* (*cinI*) occurs by binding of the gene product *Y* (TetR) of gene *y* (*tetR*) to a single promoter P_Y_ (P_tet_). Symmetrically, activation of the expression of gene *y* (*tetR*) occurs by binding of autoinducer R (C4-HSL) to promoter P_R_ (P_rhl_), while inhibition of the transcription of both genes *y* (*tetR*) and *w* (*rhlI*) occurs by binding of X (LacI) to a single promoter P_X_ (P_lac_). **A design** (*bottom panel*): Activation of the expression of gene *x* (*lacI*) occurs by binding of autoinducer R (C4-HSL) to promoter P_R_ (P_rhl_). Expression of genes *y* (*tetR*) and *w* (*rhlI*) is driven by a common single promoter P_X_. Gene products *U* and *W* are synthases CinI and Rhil, respectively. Gray horizontal strips correspond to integration plasmids. Genes *gfp* and *rfp* correspond to green and red florescent proteins, GFP and RFP, respectively.

For the sake of completeness of our description, we begin our discussion of the S toggle and A toggle designs (Fig 1) with two classical orthogonal repressors (Table 1):

1. LacI from *E. coli* which inhibits the transcription of the second repressor gene, tetR from the tetracycline-resistance transposon Tn10;
2. TetR which represses the transcription of the first repressor gene *lacI*.

**Table 1:** A toggle molecular part catalog (explanations of variables are given in S model).

Name	variable	Function	Description	References
*lacI*	–	repressor gene	lactose-inducible transcriptional repressor from *E. coli*	[1, 2, 6, 33]
*tetR*	–	repressor gene	from the tetracycline-resistance transposon Tn10	[1, 2, 33, 36]
*cinI*	–	autoinducer gene	encodes protein CinI which synthesizes C14-HSL	[11, 13, 14]
*rhlI*	–	autoinducer gene	encodes protein RhlI which synthesizes C4-HSL	[11, 12]
LacI	*x*_*i*_	lactose inhibitor	a DNA-binding protein encoded by *lacI*	[1, 2, 33, 37]
TetR	*y*_*i*_	repressor protein	a basic element of tetracycline-controlled regulation	[1, 2, 34, 36]
CinI		synthase	the gene product of gene *cinI*	[11, 13, 14]
RhlI		synthase	the gene product of gene *rhlI*	[11, 12]
C14-HSL	*g*_*i*_, *g*_*e*_	autoinducer	*N*-(3-hydroxy-7-*cis*-tetradecenoyl)-L-Homoserine Lactone	[13]
C4-HSL	*r*_*i*_, *r*_*e*_	autoinducer	*N*-butyryl-L-Homoserine Lactone	[12, 24]

Next, the communication network among all toggles (Fig 1) is built upon two quorum-sensing (QS) signaling molecules (Table 1):

1. *N*-(3-hydroxy-7-*cis*-tetradecenoyl)-L-homoserine lactone (3-OH-C14-HSL);
2. *N*-butanoyl-l-homoserine lactone (C4-HSL).

For the sake of brevity, the QS signaling molecules are called autoinducers G (C14-HSL) and R (C4-HSL). Note that the G- and R-signals (acylated homoserine lactones) are natural biological signals secreted by Gram-negative bacteria, including *E. coli*, as a means of coordinating cellular activity [4, 11].

Finally, to drive the autoinducer concentrations, two synthases are used (Table 1):

1. CinI, the gene product of *cinI*, driving the concentration of C14-HSL;
2. RhlI, the gene product of *rhlI*, driving the concentration of C4-HSL.

Using the above molecular species, we implement and study two different toggle designs called *symmetric* (S) and *asymmetric* (A) designs, respectively, (Fig 1):

1. In the S design, each of the two autoinducers activates symmetrically the transcription of the corresponding repressor gene through a single promoter, that is, promoter P_cin_ (P_G_) for gene *lacI* (*x*) and promoter P_rhl_ (P_R_) for gene *tetR* (*y*);
2. In the A design, the same repressor genes (as used in the S design) antagonistically repress one another directly, while there is only one autoinducer that asymmetrically facilitates communication between all toggles.

The genetic circuit topology used in the A design (Fig 1) is taken from [16]. In order to keep making a fair comparison with the S design, we have replace the *luxR*-*luxI* system considered in [16] by the *lacI*-*tetR* system suggested in [1], see SI-1 Toggle B2 in S1 Text. Note that both CinI and RhiI are homologous to LuxI [38].

To host the S and A toggles, we use *E. coli*, a bacterial cell which has been well-studied in a huge number of relevant experimental and modeling works [32, 39–50], and which has been widely used to implement and test various synthetic circuits [1, 2, 4, 7–9, 15]. A practical modeling reason for this selection is narrowing-down our search for biologically-meaningful parameters to values known from the *E. coli* studies. However, our conclusions do not depend in any way on biological properties of the host.

As a readout of the toggle state in individual cells, we further assume that each *E. coli* cell contains a gene encoding a spectrally distinct fluorescent reporter, GFP for gene *lacI*, and RFP for gene *tetR*, driven by promoters that respond to the autoinducers C14-HSL and C4-HSL, respectively. We do not simulate the processes of bio-synthesis and degradation of the fluorescent proteins explicitly, using appropriate cascade models, for two reasons: *(i)* the “reporter” submodel does not affect the dynamics of the entire model, and *(ii)* the half-lives of the reporter proteins can be made similar to the half-lives of the repressor proteins [2].

Finally, because each toggle can either be in a state where *(a)* LacI protein is abundant, while TetR protein is scarce, or in a state where *(b)* TetR protein is abundant, while LacI protein is scarce, we call state *(a)* a green state or, simply, a G-state and state *(b)* a red state or, simply, an R-state, respectively.

#### S model

A mathematical model describing a population of identical S toggles is

$$\frac{dx_{i}}{dt}=\;\gamma_{x}\;+\;\frac{a_{1}}{1+y_{i}^{n_{Y}}}\;+\;\frac{a_{3}\;g_{i}^{n_{G}}}{1+g_{i}^{n_{G}}}\;-\;x_{i},$$ (1)

$$\frac{dy_{i}}{dt}=\;\gamma_{y}\;+\;\frac{a_{2}}{1+x_{i}^{n_{X}}}\;+\;\frac{a_{4}r_{i}^{n_{R}}}{1+r_{i}^{n_{R}}}\;-\;y_{i},$$ (2)

$$\frac{dg_{i}}{dt}=\;\gamma_{g}\;+\;\frac{a_{5}}{1+y_{i}^{n_{Y}}}\;+\;d\left(g_{e}-g_{i}\right)\;-\;\delta_{g}\;g_{i},$$ (3)

$$\frac{dr_{i}}{dt}=\;\gamma_{r}\;+\;\frac{a_{6}}{1+x_{i}^{n_{X}}}\;+\;d\left(r_{e}-r_{i}\right)\;-\;\delta_{r}\;r_{i},\;i=1,\ldots ,N,$$ (4)

$$\frac{dg_{e}}{dt}=\;\frac{\rho }{N}\sum_{i=1}^{N}d\left(g_{i}-g_{e}\right)\;-\;\delta_{e}\;g_{e},\;0\leq \rho \leq 1,$$ (5)

$$\frac{dr_{e}}{dt}=\;\frac{\rho }{N}\sum_{i=1}^{N}d\left(r_{i}-r_{e}\right)\;-\;\delta_{e}\;r_{e}.$$ (6)

Here, all state variables and parameters are dimensionless, and are obtained from the corresponding biologically meaningful state variables and parameters describing the *lac-tet* system (Table 1) after an appropriate nondimensionalization, see SI-2 Model Derivation in S1 Text.

In the S model Eqs (1)–(6), *t* is dimensionless time; *x*_*i*_ and *y*_*i*_ are the dimensionless concentrations (levels) of intracellular repressor proteins LacI and TetR, respectively; *g*_*i*_ and *r*_*i*_ are the dimensionless concentrations of intracellular autoinducers C14-HSL and C4-HSL, respectively; *g*_*e*_ and *r*_*e*_ are the dimensionless concentrations of extracellular autoinducers C14-HSL and C4-HSL, respectively.

The dimensionless rate constants *a*_*i*_, *i* = 1, …, 6, depend on the copy numbers of the plasmids that bear the corresponding genes, see relationships Eqs (18) and (19) given in Scaling; *n*_X_, *n*_Y_, *n*_G_, and *n*_R_ are the corresponding Hill coefficients; *d* is the dimensionless diffusion coefficient; *δ*_*g*_ and *δ*_*r*_ are the dimensionless lumped dilution-degradation rates due to the exponential cell growth and degradation of the corresponding species; *γ*_*x*_, *γ*_*y*_, *γ*_*g*_, and *γ*_*r*_ are the corresponding leakiness coefficients [16, 17].

The degradation rate constants for repressor species *x*_*i*_ and *y*_*i*_ are scaled out to unity, as it is done in [1, 2, 16, 17], see SI-3 Estimation of Parameter Values in S1 Text; *δ*_*e*_ is the dilution rate due to flow in the medium; *ρ* is a population density; and *N* is the number of cells in the given population.

#### A model

A dimensionless mathematical model describing a population of identical A toggles is

$$\frac{dx_{i}}{dt}=\;\gamma_{x}\;+\;\frac{a_{1}}{1+y_{i}^{n_{Y}}}\;+\;\frac{a_{4}\;r_{i}^{n_{R}}}{1+r_{i}^{n_{R}}}\;-\;x_{i},$$ (7)

$$\frac{dy_{i}}{dt}=\;\gamma_{y}\;+\;\frac{a_{2}}{1+x_{i}^{n_{X}}}\;-\;y_{i},$$ (8)

$$\frac{dr_{i}}{dt}=\;\gamma_{r}\;+\;\frac{a_{6}}{1+x_{i}^{n_{X}}}\;+\;d\left(r_{e}-r_{i}\right)\;-\;\delta_{r}\;r_{i},\;i=1,\ldots ,N,$$ (9)

$$\frac{dr_{e}}{dt}=\;\frac{\rho }{N}\sum_{i=1}^{N}d\left(r_{i}-r_{e}\right)\;-\;\delta_{e}\;r_{e}.$$ (10)

Here, all state variables and parameters are as defined for the S model Eqs (1)–(6). The A model is mathematically identical to the *minimal* (simplified) model developed in [16] for the *E.coli* strain (toggle) B2 [4], shown in Fig SI-1.1 in S1 Text.

#### Modeling assumptions

Since the intention of our work is to illustrate the application of monotone dynamical systems theory to the S design and, also, to compare the S design with other known designs, we have developed two *simplified minimal* models, described in S model and A model, respectively. However, because a practical implementation of synthetic toggles is still far from being a routine exercise [6], care should be taken to explain the assumptions used to construct the models.

First of all, we compare the new *monotone* S design with a well accepted *non-monotone* toggle design, the *E.coli* strain B2 [4], which we call the A design, and for which a substantial modeling work has been done [16, 17] (SI-1 Toggle B2 in S1 Text). Therefore, for a careful comparison of these two different designs, we have accepted the corresponding modeling simplifications and assumptions [16, 17]. We discuss them in SI-1 Toggle B2 and SI-2.1 Mass-Balance Equations in S1 Text.

Models always involve simplifications of reality. The impact of several such simplifications and assumptions, particularly those impacting monotonicity properties and bistabiliy regions, are: (*i*) a reduced promoter leakiness [2, 4, 51], (*ii*) unsaturated levels of AAA+ proteases ClpXP [9], and (*iii*) small values of Hill coefficients [7–9, 16]. Because the impact of the variation in the values of the Hill coefficients and leakiness parameters on the bistability region in the A model is carefully analyzed in [16], we validate variations in these parameters for the S model only.

Other assumptions refer to the cells growth conditions, *e.g.*, whether the cells are in solid culture, liquid culture, in a micro-chemostat, on plates, etc. To this end, the A model [16] corresponds to the strain B [4] for which a detailed description of the growth conditions can be found in the corresponding experimental protocol [4]. We can thus assume that both S and A models correspond to the same growth condition, that is, the aerobic growth in LB medium on plates [4].

In both S and A models, there is no time-dependence of dilution or degradation [16]. However, depending on the growth phase of the bacteria, the effects of dilution will not be constant [40, 47–49, 52]. We use the stationary exponential growth assumption for the sake of simplicity [16].

### Model Parameters

Uncertainty about the values of parameters characterizing molecular components of synthetic circuits always presents a significant difficulty in circuit design [2]. Here, we discuss reference values of dimensionless parameters obtained using an appropriate scaling procedure. We also explain how we select and interpret parameters for our bifurcation analysis.

#### Model parameters

Reference values of all parameters used in our modeling studies are estimated in SI-3 Estimation of Parameter Values in S1 Text, and these correspond to half-lives of all proteins in the range 4–10 min., which are close to a typical mRNA half-life in *E. coli* [2]. Also, to avoid competition for ribosomes [43], only a few plasmids bearing four promoters P_X_, P_Y_, P_G_, and P_R_ can be used, and we use 1–2 copies per cell, see SI-3 Estimation of Parameter Values in S1 Text. The *E. coli* replication period is assumed to be around 25 min.

Despite the fact that much is known about *E. coli* [39–41, 45, 47, 48, 50], it is not possible to model behavior in a quantitatively precise way, since not enough is yet known about molecular interactions between the toggle and the host cell to make such a description realistic [6]. Instead, we hope to identify classes of toggle designs and dynamic behaviors to determine which of the designs could lead to an improved self-synchronization reliability and an improved capability for self-correction of spontaneous synchronization errors, when a small fraction of cells flips to the opposite (undesirable) transcriptional signature state, see Spontaneous Synchronization Errors. We will also make some predictions that might help to facilitate engineering toggles with desired robust traits.

In our computational analysis, the following set of *reference* parameter values is used:

$$\gamma_{x}=\;\gamma_{y}\;=\;\gamma_{g}\;=\;\gamma_{r}\;=\;0,$$ (11)

$$a_{1}=\;a_{2}\;=\;20,\;a_{3}\;=\;a_{4}\;=\;10,\;a_{5}\;=\;a_{6}\;=\;3,$$ (12)

$$n_{X}=\;n_{Y}\;=\;n_{G}\;=\;n_{R}\;=\;3,$$ (13)

$$\delta_{g}=\;\delta_{r}\;=\;1.0,\;\;\delta_{e}\;=\;0.5,\;\rho \;=\;0.8.$$ (14)

Groups of parameters with identically the same values are used to introduce the toggle mirror (involutive) symmetry into the S model as discussed in SI-5 Symmetry in S1 Text. We find that the working values of parameters estimated in Eqs (11)–(14) are within the range of equivalent parameters (rate constants, Hill coefficients, *etc.*) used earlier for genetic circuits built from similar (*e.g.*, homologous) molecular entities [1, 2, 4, 7–9, 16, 17, 28–31, 53].

However, there is a variability in the estimation of values for some important parameters in the literature. Specifically, the values of the Hill coefficients (*n*_G_ and *n*_R_) for the binding of C4-HSL and C14-HSL to the corresponding promoters are equal to 4 (estimated *ad hoc*) in the model developed in [9]. On the other hand side, the values of the Hill coefficients for C6-HSL and C12-HSL are estimated in the range of values 1–2 in [7, 8]. Because it may be unlikely to have high values of the Hill coefficients, and because the analytical and computational analyses of the impact of the Hill coefficients on the bistability regions for the A model have been done in great detail in [16], we have selected a compromising *reference* values of all Hill coefficients equal to 3 [16].

Another important parameter (*γ*) is the promoter leakiness [2, 4, 51]. To this end, very small dimensionless values of the corresponding leakiness parameters are used in the relevant modeling studies [16, 17]. Because we compare our S model with the A model [16], and because the zero reference value for the leakiness parameters is used in [16], we also use the zero *reference* value for the leakiness parameters in both the S and A models. The tightness of the promoter control has been reported in the literature, *e.g.*, see [2, 4, 12, 51], and the reduction in the promoter leakiness is the subject of active ongoing research with the promise to reduce this leakiness even further dramatically [51].

To probe the robustness of the developed theory in the cases when: (*i*) the Hill coefficients can take on different values, (*ii*) the promoter leakiness can be allowed to take on nonzero values, and (*iii*) the S model can lose its “perfect” mirror symmetry property, we additionally analyze the S model with the following modified parameter values while all other parameter values are kept intact as in the reference set Eqs (11)–(14),

$$\gamma_{x}=\;\gamma_{y}\;=\;\gamma_{g}\;=\;\gamma_{r}\;=\;0.01,$$ (15)

$$a_{1}=\;a_{2}\;=\;100,$$ (16)

$$n_{X}=\;n_{Y}\;=\;2,\;n_{G}\;=\;1,\;\text{and}\;n_{R}\;=\;2.$$ (17)

Specifically, condition *n*_G_ ≠ *n*_R_ removes the mirror symmetry from the S model. We will also use the set of modified parameters in the case when the monotonicity property is violated by the sequestration of AAA+ proteases ClpXP (Monotone Parametric Dependencies in the S Design).

#### Scaling

One of the goals of a model nondimensionalization and scaling is to reduce the number of (correlated) parameters by lumping original parameters into a smaller parameter set. In this case, interpretation of changes in the values of dimensionless parameters should be done carefully, as the set of dimensionless parameters is usually not in one-to-one correspondence with the set of original parameters. For example, mathematical models used for synthetically engineered systems often contain parameters representing multiple biological parts and, so, tuning a dimensionless parameter in the corresponding mathematical model can be implemented experimentally in a number of different ways [6].

The dimensional and dimensionless parameters used in the S and A toggle models are related to one another by the following relationships (see SI-2 Model Derivation in S1 Text):

1. For the dimensionless rate parameters, we obtain:
  $$a_{1}=\;\frac{b_{x}\;k_{x}\;\left[P_{Y}\right]}{K_{X}\left(r_{d}+\mu \right)},\;a_{2}\;=\;\frac{b_{y}\;k_{y}\;\left[P_{X}\right]}{K_{Y}\left(r_{d}+\mu \right)},\;a_{3}\;=\;\frac{b_{x}\;k_{x}\;\left[P_{G}\right]}{K_{X}\left(r_{d}+\mu \right)},$$ (18)
  $$a_{4}=\;\frac{b_{y}\;k_{y}\;\left[P_{R}\right]}{K_{Y}\left(r_{d}+\mu \right)},\;a_{5}\;=\;\frac{b_{u}\;k_{u}\;k_{G}\;\left[P_{Y}\right]}{K_{G}{\left(r_{d}+\mu \right)}^{2}},\;a_{6}\;=\;\frac{b_{w}\;k_{w}\;k_{R}\left[P_{X}\right]}{K_{R}{\left(r_{d}+\mu \right)}^{2}}.$$ (19)
2. For the dimensionless diffusion and degradation parameters, we obtain:
  $$d_{g}=\;\frac{D_{G}}{r_{d}+\mu },\;d_{r}\;=\;\frac{D_{R}}{r_{d}+\mu },$$ (20)
  $$\delta_{g}\;=\;\frac{r_{G}+\mu }{r_{d}+\mu },\;\delta_{r}\;=\;\frac{r_{R}+\mu }{r_{d}+\mu },\;\delta_{e}\;=\;\frac{\mu_{e}}{r_{d}+\mu }.$$ (21)

Let us briefly discuss Eqs (18), (19), (20) and (21). Here, the burst parameter *b*_*x*_ for the protein X or, equivalently, LacI, depends on the efficiency of translation, controlled by strength of ribosome-binding sites (RBS) [1, 6], and the mRNA half-life time [54]; [P_X_] is the number of promoters per cell for gene *x*; *k*_*x*_ is an average transcription rate for gene *x* (*lacI*); *K*_X_ is the number of LacI proteins required to half-maximally repress P_lac_; *k*_*G*_ is the maximum production rate of C14-HSL by CinI, *D*_G_ is the export rate of C14-HSL; *μ* is the intracellular specific dilution rate due to the host cell growth, *μ* = ln2/*T*, *T* is the division period. Parameters for other proteins and QS signaling molecules are defined similarly, see SI-2 Model Derivation and SI-3 Estimation of Parameter Values in S1 Text. Based on the fact that N-Acyl Homoserine Lactone Lactonase (AHL-lactonase) hydrolyzes C4-HSL effectively [55], we also assume that specific degradation rate constants for the signaling molecules, C14-HSL and C4-HSL, can be set experimentally [6], corresponding to the parameter values used in our models. We pick these specific promoters and autoinducers in order to be concrete and to justify biologically meaningful values of the model parameters. However, we wish to emphasize that our results are generic for the architectures shown in Fig 1.

#### Bifurcation parameters

In our bifurcation analysis, we use almost all dimensionless parameters given in Eqs (11)–(14) as free parameters allowed to be varied to detect changes in stability of the corresponding solutions. Whenever a new bifurcation point is detected, we provide an appropriate interpretation in terms of interventions reviewed in [6], which can potentially lead to the corresponding effect.

For example, suppose that *d* is the free parameter used in bifurcation analysis. Due to the relationships Eqs (20)–(21), changes in the values of *d* may correspond to different and independent experimental interventions [6] leading to: (*a*) changes in the membrane permeability (*i.e.*, *D*_G_ and *D*_R_), or (*b*) changes in the half-lives of repressor proteins, or (*c*) changes in the specific growth rate of the host cell. As such, both higher values of protein half-lives and diffusion permeability as well as lower values of the specific growth rate (longer replication periods) correspond to higher values of the parameter *d*. Recall that the value of the parameter *d* characterizes the strength of the interaction between cells in the given population, which facilitates self-synchronization [15–17, 25, 56].

More broadly, we can rely upon the fact that all dimensionless parameters are defined via appropriate combinations of the original dimensional parameters Eqs (18), (19), (20) and (21) in our interpretation of results obtained from bifurcation analysis as follows.

The values of dimensionless rate parameters (*i.e.*, *a*-parameters) can be changed by decreasing or increasing translational efficiency, which depends on the nucleotide sequence of the ribosome binding sites (RBS) located within the upstream noncoding part of the mRNA [1, 50]. The values of dimensionless rate parameters can also be changed by decreasing or increasing the lifetime values of appropriate proteins. Indeed, a carboxy-terminal tag, based on ClpX, the ATP-dependent unfoldase/translocase of ClpXP recognizing specific protein substrates bearing *ssrA* tags [9, 57], can be inserted at the 3W end of each repressor gene [2]. Proteases in *E. coli* recognize this tag and target the attached protein for destruction. Such tags are used to reduce the half-life of the proteins from more than 60 min to around 4 min, which makes it possible and (also convenient) to set the half-life times for all toggle proteins (approximately) equal to one another and close to the half-lives of mRNAs [2, 15]. We assume that all *ssrA* tagged proteins do not compete for AAA+ protease ClpXP [9], in which case the sequestration of AAA+ protease ClpXP is negligible small and is not modeled. To this end, both RBS and carboxy-terminal tags are the principal tools by which the parameters of an engineered gene network can be adjusted experimentally [1, 2, 6].

### Stability and Bifurcation in Cellular Populations

A number of powerful concepts and software tools have been developed to efficiently analyze bifurcations of equilibrium solutions in small-scale and medium-sized dynamical models [58–61]. To this end, however, the analysis of bifurcations in the A and S models already becomes a formidable task in terms of CPU loads at *N* = 10. For example, the S model describing 10 coupled toggles includes 42 ODEs. Therefore, special conceptual and computational approaches need to be developed to interpret results of modeling with A and S models for cellular populations consisting of thousands or even millions of cells.

Fortunately, due to the special structure of the Jacobian matrices for the corresponding linearizations of the A and S models, the computation of the characteristic polynomials, which are used to evaluate stability and bifurcation [26, 62], can first be conceptually and, then, numerically simplified, by employing Schur’s formula [63]. As a result, (*i*) the stability and bifurcation analyses of homogeneous populations for any *N* ≥ 2 can be rigorously reduced to the case of a population consisting of only *two* toggles, (*ii*) the analysis of a (1:1)-mixed state for any even *N* ≥ 4 can be rigorously reduced to the case of only *three* toggles, and (*iii*) the analysis of a (*N*_1_: *N*_2_)-mixed state with any *N*_1_ ≠ *N*_2_ and *N*_1_ + *N*_2_ = *N* can be rigorously reduced to the case of only *four* toggles as described in SI-6 Exponential Stability of Cellular Populations in S1 Text.

Schur’s formula [63] also helps to solve another important nontrivial specificity of the A- and S-population models caused by multiplicity of eigenvalues due to the model’s symmetry discussed in SI-6 Exponential Stability of Cellular Populations in S1 Text. Computationally, in the case of multiple eigenvalues caused by symmetry, the standard tools [58–61] cannot be used in a straightforward way, when a special care should be taken. Our theoretic developments can aid in the analysis and interpretation of all such and similar cases arising in modeling of cellular populations, see SI-6 Exponential Stability of Cellular Populations in S1 Text for more rigorous definitions and results.

Indeed, the exact (very large) number of cells, *N*, in a cell culture is usually unknown, as cells can die or even be washed out. In such cases, the population density parameter *ρ* is used, and, therefore, stability of and bifurcation in populations with respect to the variability in their densities is done. The corresponding changes in the integer parameter *N* that reflect changes in *ρ* assume a formal study of stability with respect to changes in the number of differential equations in the corresponding models. This is an ill-defined perturbation in the number of equations, and we show how it can be avoided by using the stability approach developed in SI-6 Exponential Stability of Cellular Populations in S1 Text.

### Spontaneous Synchronization Errors

**Fig 2:**
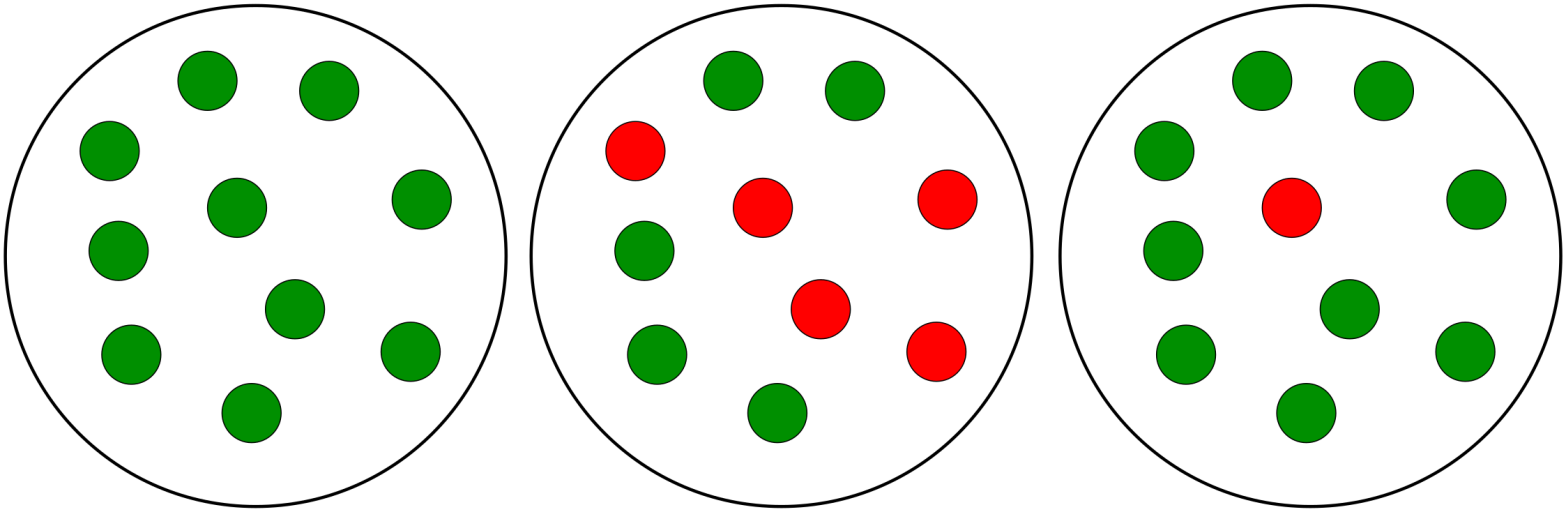
Homogeneous and heterogeneous (mixed) populations. An example of a population consisting of 10 cells is shown. The left panel demonstrates a homogeneous G-population. The center panel demonstrates a heterogeneous (1:1)-population, where the homogeneous G- and R-subpopulations have equal number of cells. The right panel demonstrates a heterogeneous (9:1)-population formed of two unequal subpopulations which represent a *spontaneous synchronization error*, when one or a few toggles spontaneously flip from green (G) to red (R) states.

Capabilities of toggles to fail and recover from spontaneous synchronization errors can be formalized in terms of a *multistability* concept, that is, as a co-existence of bistable *homogeneous* populations and various *heterogeneous* populations (Fig 2), also called *mixed* states, under the same conditions. Recall that mixed states are known to lead to bistable distributions [4].

Following [4], we call a population heterogeneous or, equivalently, mixed if it comprises toggles with *different* transcription signatures for the *same* genes: (*i*) the repressor gene *lacI* is active (G-state), while *tetR* is repressed, and (*ii*) *lacI* is repressed, while the repressor gene *tetR* is active (R-state), see Toggle Designs. In other words, a homogeneous population is fully characterized by either transcription signature (*i*) or (*ii*), while a heterogeneous population is characterized by mixed signatures (*i*) and (*ii*) simultaneously present in the population (Fig 2).

Different heterogeneous populations can be characterized by transcription signature “mixtures” with ratio (*p*:*q*), *p* + *q* = 1, describing the fraction of toggles in the G-state versus the fraction of toggles in the R-state within the same population. For homogeneous populations, we, therefore, have either (1:0) or (0:1) transcriptional signature (Fig 2).

With these concepts, we can formulate more precisely our objective: to find conditions under which heterogeneous (mixed) population equilibrium solutions can loose their stability or can even be eliminated completely.

As a proof of concept, an example of an (9:1)-heterogeneous population (Fig 2) will be used, where the number of toggles in the first, Green-subpopulation (G) (*tetR* is suppressed) is 9 times bigger that the number of toggles in the second, Red-subpopulation (R) (*lacI* is suppressed). In this simplest case, the G-subpopulation comprises 9 cells (*p* = 0.9 or 90%-fraction of all cells), while the R-subpopulation comprises one cell (*q* = 0.1 or 10%-fraction of all cells).

Note that our analysis of (9:1)-mixed states does not depend on the number of cells *N* in the entire population, which is usually unknown in experiments. In other words, our results hold for any integers *N*, *N*_1_, and *N*_2_, such that *N* = *N*_1_ + *N*_2_, and *N*_1_: *N*_2_ = 9: 1, where the fractions of cells with different transcription signatures are defined by the numbers *p* = *N*_1_/*N* and *q* = *N*_2_/*N*, respectively, see SI-6 Exponential Stability of Cellular Populations in S1 Text.

### Monotone Systems Formalism

The systems considered here are described by the evolution of states, which are time-dependent vectors *x*(*t*) = (*x*_1_(*t*), …, *x*_*n*_(*t*)). The components *x*_*i*_ represent concentrations of chemical species (such as proteins, mRNA, metabolites, and so forth), the dynamics of which are given by a system of ODE’s:

$$\begin{array}{ccc} \frac{dx_{1}}{dt}\left(t\right) & = & f_{1}\left(x_{1}\left(t\right),x_{2}\left(t\right),\ldots ,x_{n}\left(t\right)\right),\\ \frac{dx_{2}}{dt}\left(t\right) & = & f_{2}\left(x_{1}\left(t\right),x_{2}\left(t\right),\ldots ,x_{n}\left(t\right)\right),\\ & \vdots & \\ \frac{dx_{n}}{dt}\left(t\right) & = & f_{n}\left(x_{1}\left(t\right),x_{2}\left(t\right),\ldots ,x_{n}\left(t\right)\right)\;. \end{array}$$

We also write simply *dx*/*dt* = *f*(*x*), where *f* is a differentiable vector function with components *f*_*i*_. The coordinates *x*_*i*_(*t*) are non-negative numbers. We write *φ*(*t*, *x*_0_) for the solution of the initial value problem $\dot{x}\left(t\right)=f\left(x\left(t\right)\right)$ with *x*(0) = *x*_0_, or just *x*(*t*) if *x*_0_ is clear from the context, and assume that this solution *x*(*t*) exists and remains bounded for all *t* ≥ 0.

#### Definition of monotone systems

A system is said to be *monotone* if there exists a partition of the set of indices of state variables:

$$\left\{1,2,\ldots ,n\right\}\;=\;S_{+}⋃S_{-}\;\left(S_{+}⋂S_{-}=\emptyset \right)$$

with the following properties:

1. for each pair of indices {*i*, *j*} ∈ *S*_+_ (*i* ≠ *j*) and each pair of indices {*i*, *j*} ∈ *S*_−_ (*i* ≠ *j*),
  $$\frac{\partial f_{i}}{\partial x_{j}}\left(x\right)\geq 0\;\forall \;x$$
2. and for each pair of indices {*i*, *j*} such that *i* ∈ *S*_+_ and *j* ∈ *S*_−_ and each pair of indices {*i*, *j*} such that *i* ∈ *S*_−_ and *j* ∈ *S*_+_,
  $$\frac{\partial f_{i}}{\partial x_{j}}\left(x\right)\leq 0\;\forall \;x\;.$$

Observe that the definition does not impose any constrains on diagonal entries $\frac{\partial f_{i}}{\partial x_{i}}\left(x\right)$. These may have arbitrary signs, even depending on *x*.

Monotone systems [23, 64, 65] were introduced by Hirsch, and constitute a class of dynamical systems for which a rich theory exists. (To be precise, we have only defined the subclass of systems that are “monotone with respect to some orthant order” but the notion of monotone dynamics can be defined with respect to more general orders.)

We assume from now on that our system satisfies the following property: for each pair of distinct nodes *i* and *j*, one of these holds:

1. $\frac{\partial f_{i}}{\partial x_{j}}\left(x\right)>0$ for all states *x*
2. $\frac{\partial f_{i}}{\partial x_{j}}\left(x\right)<0$ for all states *x*
3. $\frac{\partial f_{i}}{\partial x_{j}}\left(x\right)=0$ for all states *x*.

Of course, there are many models for which partial derivatives may change sign depending on the particular point *x*. With assumptions (1–3), however, the main results that we need from monotone dynamical systems theory will be particularly easy to state.

Monotone systems cannot admit any stable oscillations [19, 21, 66]. Under a stronger property, described next, only convergence to steady states is generically possible.

#### Strong monotonicity

The directed *species influence graph* *G* associated to a system with *n* state variables is defined as follows. The graph *G* has *n* nodes (or “vertices”), which we denote by *v*_1_, …, *v*_*n*_, one node for each species. If

$$\frac{\partial f_{i}}{\partial x_{j}}>0\;\text{(activation),}$$

we introduce an edge labeled “1” from *v*_*j*_ into *v*_*i*_. If, instead,

$$\frac{\partial f_{i}}{\partial x_{j}}<0\;\text{(inhibition),}$$

we introduce an edge labeled “−1” (or just “−”) from *v*_*j*_ into *v*_*i*_. Finally, no edge is drawn from node *v*_*j*_ into node *v*_*i*_ if the partial derivative $\frac{\partial f_{i}}{\partial x_{j}}\left(x\right)$ vanishes identically (no direct effect of the *j*th species upon the *i*th species). An alternative is to write a normal arrow “→” or a blunted arrow “⊣” (or an arrow labeled “−”) respectively for the first two cases. The graph *G* is an example of a *signed graph* [67], meaning that its edges are labeled by signs.

No self-edges (edges from a node *v*_*i*_ to itself) are included in the graph *G*, whatever the sign of the diagonal entry ∂*f*_*i*_/∂*x*_*i*_ of the Jacobian. The sign of this derivative may be positive, negative, or even be state-dependent. Results will not depend on signs of diagonals of the Jacobian of *f*.

The graph *G* is said to be *strongly connected* if, given an arbitrary pair of different indices {*i*, *j*}, there is a some, possibly indirect, effect of *i* on *j*. Formally, we ask that there is a sequence of indices *i* = *k*_0_, *k*_1_, …, *k*_*r*_ = *j* such that

$$\frac{\partial f_{k_{s+1}}}{\partial x_{k_{s}}}\neq 0\;\text{for}\;s=0,\ldots ,r-1\;.$$

A system is said to be *strongly monotone* if it is monotone and, in addition, its species influence graph *G* is strongly connected. (As with the definition of monotonicity, one can extend strong monotonicity to far more general classes of systems, but we use a more restrictive notion that makes results less technical to state.) Even when there are multiple steady-states, the Hirsch Generic Convergence Theorem [21, 23, 64, 65] is a fundamental result.

#### Hirsch’s Theorem

Even though they may have arbitrarily large dimensionality, monotone systems behave in many ways like one-dimensional systems: Hirsch’s Theorem asserts that generic bounded solutions of strongly monotone differential equation systems must converge to the set of (stable) steady states. “Generic” means here “for every solution except for a measure-zero set of initial conditions.” In particular, no nontrivial attractors arise. The genericity qualifier is needed in order to exclude the unstable manifolds of saddles as well as behavior on lower-dimensional sets [18].

The general theory of monotone systems applies to a class of differential equations somewhat larger than the one considered here. What we defined as monotone systems are, to be more precise, “systems monotone with respect to an orthant order’’. It is possible to, more generally, define systems that are monotone with respect to orders induced by arbitrary convex proper cones. However, the generality that one obtains in that fashion comes at the cost of conditions which are typically very difficult to verify in examples and, in any event, this generality is not needed for the purpose of analyzing the systems that are the focus of this paper.

## Results and Discussion

To carry out computational bifurcation analysis, MatCont [59, 68] has been used. A technical description of bifurcation points can be found in [58, 59, 62, 68].

### Application of Monotone Systems Theory to the S Design

To apply monotone systems theory to the S toggle model Eqs (1)–(6), we first rewrite the model in the following convenient general form with 4*N* + 2 variables:

$$\begin{array}{ccc} \frac{dx_{i}}{dt} & = & h_{x}\left(x_{i},y_{i},g_{i}\right),\\ \frac{dy_{i}}{dt} & = & h_{y}\left(x_{i},y_{i},r_{i}\right),\\ \frac{dg_{i}}{dt} & = & h_{g}\left(y_{i},g_{i},g_{e}\right),\\ \frac{dr_{i}}{dt} & = & h_{r}\left(x_{i},r_{i},r_{e}\right),\\ \frac{dg_{e}}{dt} & = & H_{g}\left(g_{e},g_{1},\ldots ,g_{N}\right),\\ \frac{dr_{e}}{dt} & = & H_{r}\left(r_{e},r_{1},\ldots ,r_{N}\right). \end{array}$$

Here, *i* = 1, …, *N*, all the functions in the right-hand side are differentiable, and the following signs hold for partial derivatives, everywhere in the state space:

$$\frac{\partial h_{x}}{\partial x_{i}}<\;0,\;\frac{\partial h_{x}}{\partial y_{i}}\;<\;0,\;\frac{\partial h_{x}}{\partial g_{i}}\;>\;0,\;\frac{\partial h_{x}}{\partial a_{1}}\;>\;0,\;\frac{\partial h_{x}}{\partial a_{3}}\;>\;0,$$ (22)

$$\frac{\partial h_{y}}{\partial x_{i}}<\;0,\;\frac{\partial h_{y}}{\partial y_{i}}\;<\;0,\;\frac{\partial h_{y}}{\partial r_{i}}\;>\;0,\;\frac{\partial h_{y}}{\partial a_{2}}\;>\;0,\;\frac{\partial h_{y}}{\partial a_{6}}\;>\;0,$$ (23)

$$\frac{\partial h_{g}}{\partial y_{i}}<\;0,\;\frac{\partial h_{g}}{\partial g_{i}}\;<\;0,\;\frac{\partial h_{g}}{\partial g_{e}}\;>\;0,\;\frac{\partial h_{g}}{\partial a_{5}}\;>\;0,\;\frac{\partial h_{g}}{\partial \delta }\;<\;0,$$ (24)

$$\frac{\partial h_{r}}{\partial x_{i}}<\;0,\;\frac{\partial h_{r}}{\partial r_{i}}\;<\;0,\;\frac{\partial h_{r}}{\partial r_{e}}\;>\;0,\;\frac{\partial h_{g}}{\partial a_{6}}\;>\;0,\;\frac{\partial h_{r}}{\partial \delta }\;<\;0,$$ (25)

$$\frac{\partial H_{g}}{\partial g_{i}}>\;0,\;\frac{\partial H_{g}}{\partial g_{e}}\;<\;0,\;\frac{\partial H_{g}}{\partial \delta_{e}}\;<\;0,$$ (26)

$$\frac{\partial H_{r}}{\partial r_{i}}>\;0,\;\frac{\partial H_{r}}{\partial r_{e}}\;<\;0,\;\frac{\partial H_{r}}{\partial \delta_{e}}\;<\;0,\;i=1\ldots ,N.$$ (27)

Next we observe that the S system is monotone, because we may partition its state variables as follows. One set consists of

$$x_{i},\;g_{i},\;g_{e},\;i=1,...,N\;,$$ (28)

and another set consists of

$$y_{i},\;r_{i},\;r_{e},\;i=1,...,N\;.$$ (29)

Moreover, the corresponding graph is strongly connected, as we have the following paths, for each two indices *i*, *j*:

$$x_{j}⊣r_{j}\to r_{e}\to r_{i}\to y_{i}⊣g_{i}\to g_{e}\to g_{i}\to x_{i}$$ (30)

which shows that one can reach any node from any other node by means of a directed path. Thus, the S model Eqs (1)–(6) is strongly monotone. We conclude as follows.

**Theorem 1** *Typical solutions of the S model* Eqs (1)–(6) *converge to steady states*.

This fundamental result is robust to parameters as well as to the functional form of the equations. It ensures that our proposed design has theoretically guaranteed global stability properties. No stable oscillations [16] can exist, nor can other (for, example, “chaotic” [25]) solution regimes arise. In addition to these global properties, it is also possible to use the theory of monotone systems in order to make qualitative predictions about bifurcation diagrams as discussed in the next section.

The monotonicity property of the S system has important consequences regarding its transient as well as asymptotic behavior. We discuss in an appendix how Kamke’s Theorem characterizes order relations for monotone systems. We explain now what these mean, explicitly, for the S system. Let *z*_*i*_(*t*) characterize the state of the *i*-th S toggle at time *t* ≥ 0, that is, *z*_*i*_(*t*) = (*x*_*i*_(*t*), *y*_*i*_(*t*), *g*_*i*_(*t*), *r*_*i*_(*t*)), *i* = 1, …, *N*. Let *Z*(*t*) characterize the state of the population of cells, *Z*(*t*) = (*z*_1_(*t*), …, *z*_*N*_(*t*), *g*_*e*_(*t*), *r*_*e*_(*t*)). Suppose that we have two initial sets, *Z*(0) and $\tilde{Z}\left(0\right)$, of values for the various expression levels of the repressor proteins, LacI and TetR, and we consider the behavior of *Z*(*t*) and $\tilde{Z}\left(t\right)$ for *t* > 0.

Now suppose that we wish to understand what is the effect of a perturbation in one of the components of the initial state *z*_*i*_(0) for S toggle *i* with some fixed *i*, 1 ≤ *i* ≤ *N*. (A similar argument can be applied to perturbations in other components of the initial state, or even simultaneous perturbations in all the components.) Suppose, for example, that we are interested in understanding the behavior starting from a state in which ${\tilde{x}}_{3}\left(0\right)\geq x_{3}\left(0\right)$ in the 3rd toggle *z*_3_. This gives rise to a new population-wide solution $\tilde{Z}\left(t\right)$, and we use a tilde to denote its coordinates, that is, $\tilde{Z}\left(t\right)\;=\;({\tilde{z}}_{1}\left(t\right),\ldots ,{\tilde{z}}_{N}\left(t\right),{\tilde{g}}_{e}\left(t\right),{\tilde{r}}_{e}\left(t\right))$, where ${\tilde{z}}_{i}\left(t\right)=\left(x_{i}\left(t\right),y_{i}\left(t\right),g_{i}\left(t\right),r_{i}\left(t\right)\right)$, *i* = 1, …, *N*. Then, using the information provided by the partition shown in Eqs (28) and (29), we can predict that, for all *t* > 0: ${\tilde{x}}_{i}\left(t\right)\geq x_{i}\left(t\right)$, ${\tilde{y}}_{i}\left(t\right)\leq y_{i}\left(t\right)$, ${\tilde{g}}_{i}\left(t\right)\geq g_{i}\left(t\right)$, ${\tilde{r}}_{i}\left(t\right)\leq r_{i}\left(t\right)$, ${\tilde{g}}_{e}\left(t\right)\geq g_{e}\left(t\right)$, and ${\tilde{r}}_{e}\left(t\right)\leq r_{e}\left(t\right)$ for all *i* = 1, …, *N*. As we will see shortly below, a similar conclusion can also be made with respect to perturbations in parameters, not merely initial states.

### Monotone Parametric Dependencies in the S Design

As a first step, we can include the eight parameters, *a*_*i*_ (*i* = 1, …, 6), *δ*_*g*_, and *δ*_*r*_, as constant state variables by formally adding the corresponding equations *da*_*i*_/*dt* = 0 (*i* = 1, …, 6), and *dδ*_*g*_/*dt* = *dδ*_*r*_/*dt* = 0 to the S-model Eqs (1)–(6). The extended S-model is a monotone system. However, this extended model has no strong monotonicity property, because the nodes corresponding to the parameters cannot be reached from other nodes, as the parametric extension violates the strong connectivity relationships Eq (30). However, this is not of any consequence, as the global stability properties of the S system are determined by constant values of the parameters. We only introduced the extended model in the context of bifurcation analysis. One might add additional constant variables to represent other parameters, such as the *d*’s. These other parameters do not lead to monotonicity, and this lack of monotonicity will have important consequences in bifurcation analysis, as we discuss later.

**Fig 3:**
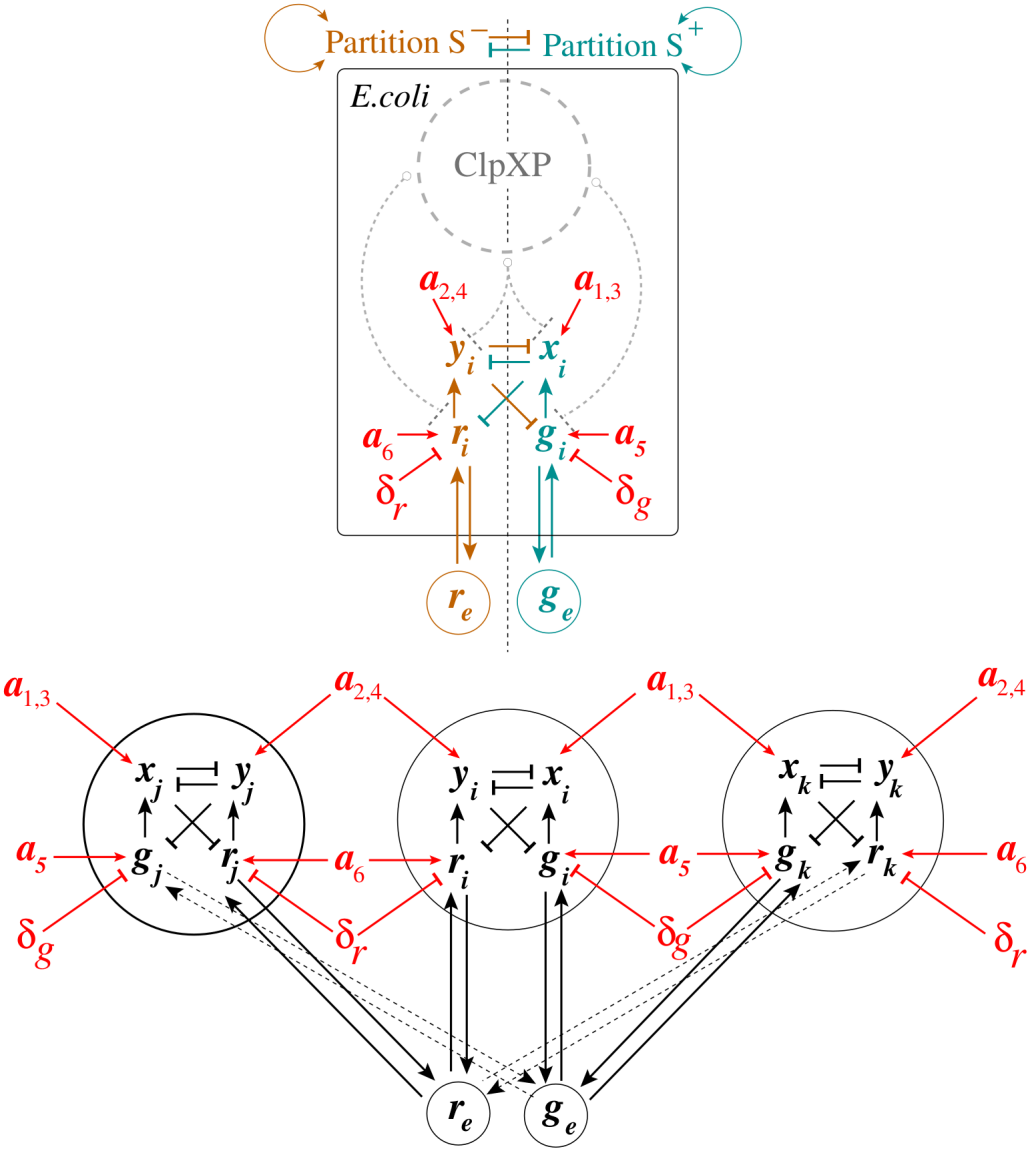
Application of Monotone Systems Theory to the S design. The top panel presents a monotonicity diagram for a single-cell S design, while the bottom panel represents an example of three identical S toggles interacting via common autoinducers, see the main text for details. In all cases, solid arrows and lines highlighted in red color correspond to monotone parameter dependencies. In the bifurcation analysis, the values of all monotone parameters are varied for all cells simultaneously.

Dependencies between the S-model state variables and parameters Eqs (22)–(27) are shown in Fig 3 (Top Panel). Here, the set of all molecular entities in the S design is partitioned into two “orthogonal” subsets, *S*^−^ and *S*^+^ (Definition of monotone systems). Solid arrows and lines highlighted in light brown color correspond to *S*^−^, while solid arrows and lines highlighted in cyan color correspond to *S*^+^. Although interactions within each subset contribute to its activate state, the orthogonal subsets repress one another. Here, ClpXP is a pool of AAA+ proteases ClpXP that use the energy of ATP binding and hydrolysis to perform mechanical work during targeted protein degradation within the cell. The corresponding inhibitory (degradation) interactions are shown, using dashed gray lines. If the circuit operates near the saturation condition for the pool of AAA+ proteases ClpXP, the S design may loss its monotone properties.

However, there is a substantial body of literature that gives theorems to the effect that “small” perturbations of monotone systems retain the properties of monotone systems, for example, a smooth regular perturbation of a strongly monotone system also has generic convergence properties [65]. A similar result as well holds for singular perturbations [69].

An example of three identical S toggles interacting via common autoinducers and operating far from the saturation of AAA+ proteases ClpXP is shown in Fig 3 (Bottom Panel).

The monotonicity of the extended model implies that stable loci in bifurcation diagrams depend monotonically on parameter variations. They will increase when the parameter being varied belongs to the component as the variable being analyzed, and will decrease if they are in different components. This property is a consequence of the general order preserving properties of monotone systems, as we explain now.

Suppose that ${\bar{x}}_{0}$ is a steady state corresponding to a parameter value *p*_0_, that is to say, $f({\bar{x}}_{0},p_{0})=0$. Suppose that we now consider *p*_1_ that is very close to *p*_0_ and larger than *p*_0_, *p*_1_ > *p*_0_. Suppose in addition that ${\bar{x}}_{1}$ is a steady state for the parameter value *p*_1_, $f({\bar{x}}_{1},p_{1})=0$, and that ${\bar{x}}_{1}$ is stable. Now pick the solution *x*_1_(*t*) of $\dot{x}=f\left(x,p_{1}\right)$ that has initial condition $x_{1}\left(0\right)={\bar{x}}_{0}$. Suppose that the extended system $\dot{x}=f\left(x,p\right)$ and $\dot{p}=0$ is monotone. Now, we may consider the following two initial states for the extended system: $\left({\bar{x}}_{0},p_{0}\right)$ and $\left({\bar{x}}_{0},p_{1}\right)$. Since the second state is larger (in the sense of Kamke’s Theorem as earlier explained) in the monotone order, it follows that the solutions satisfy $x_{1}\left(t\right)\geq {\bar{x}}_{0}$ for all *t* > 0, and therefore, taking limits, we conclude that ${\bar{x}}_{1}>{\bar{x}}_{0}$, as desired.

**Fig 4:**
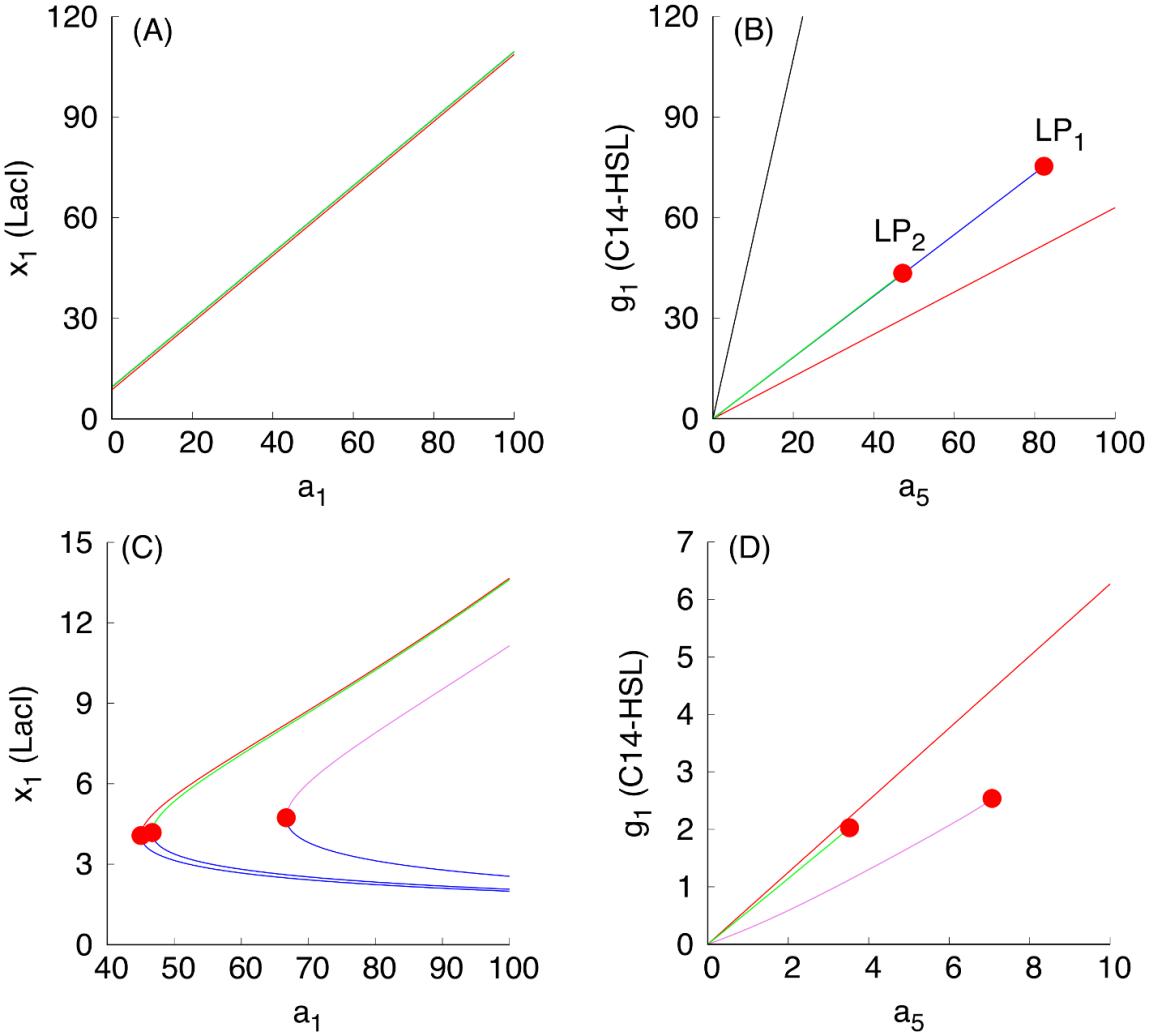
Examples of monotone parametric dependencies. Panels (A) and (B) correspond to the unsaturated S design, while Panels (C) and (D) correspond to the saturated S design. **Panels (A) and (B)**. The following color coding schema is used: (*i*) black plots are used for G-homogeneous solutions at *d* = 0.1; (*ii*) red plots are used for G-homogeneous solutions at *d* = 10; (*iii*) blue plots are used for (1:1)-mixed states at *d* = 0.1; and (*iv*) green plots are used for (9:1)-mixed states at *d* = 0.1. Red filled circles in panel (B), labeled with *LP*_1_ and *LP*_2_, correspond to Limit Point (LP) (or, equivalently, Saddle-Node) bifurcation points. Here, the blue curve connecting the origin (0, 0) and the *LP*_1_-point corresponds to the stable branch of the (1:1)-mixed state. The green curve connecting the origin (0, 0) with the *LP*_2_-point corresponds to the stable branch of the (9:1)-mixed state. Because the green curve was plotted after plotting the blue curve, a part of the blue curve is hidden beneath the green curve. Projections of the corresponding plots on 2D-planes often overlap, mixing different colors, which should not lead to any difficulty in recognizing similar monotone (“overlapping”) dependencies. **Panels (C) and (D)**. The following color coding schema is used: (*i*) red plots correspond to stable homogeneous G-states, (*ii*) violet plots correspond to stable (1:1)-mixed states, and (*iii*) green plots correspond to stable (9:1)-mixed states. In all cases, blue plots correspond to unstable states. All red filled circles correspond to the LP bifurcations. In the panel (D), the unstable branches for both (1:1) and (9:1)-mixed states are not shown because they overlap with the stable ones.

Using Fig 3 in conjunction with the dimension analysis in terms of the relationships Eqs (18), (19), (20) and (21), certain qualitative predictions can be made about the parametric dependencies based on monotone systems theory. To benchmark the approach, we have selected, as an example, a subset of dependencies shown in Fig 3, presented in Fig 4A and 4B. Fig 4C and 4D correspond to the case when the S toggle operates under the saturation condition for the pool of AAA+ proteases ClpXP (SI-8 Modification of the S and A Models to Describe Sequestration 617 of AAA+ protease ClpXP in S1 Text.)

In Fig 4, three different stable populations are chosen: (1) an G-homogeneous population; (2) an (1:1)-mixed population; here, the levels of LacI and C14-HSL from one subpopulation (within which LacI is over-expressed) are shown; and (3) a (9:1)-mixed population (a spontaneous synchronization error); here, again, the levels of LacI and C14-HSL from the largest subpopulation (within which LacI is over-expressed) are shown. Because the stable mixed populations do not exist for large values of the parameter *d* in the cases shown in Fig 4A and 4B, we use both *d* = 0.1 (weak coupling) for all populations and, additionally, we use *d* = 10 (strong coupling) for the G-homogeneous population only. In the cases shown in Fig 4C and 4D, the mixed populations turn out to be more robust and exist at *d* = 10.

Using the S-model Eqs (1)–(6) and its sequestration version (SI-8.1) (see SI-8.1 Modification of the S Model in S1 Text) with the values of fixed parameters given in Eqs (11)–(14) and (15)–(17), respectively, we find that Fig 3 predicts monotonically increasing dependencies.

The loss of stability and disappearance of the mixed states shown in Fig 4B and 4D as *a*_5_ increases can be interpreted intuitively by the fact that an increase in *a*_5_ leads to an increase in the intracellular levels of the corresponding QS signaling molecules, which, in turn, lead to an increase of extracellular levels of the QS molecules via diffusion, thereby facilitating self-synchronization of the given population of all toggles under conditions corresponding to a stronger interaction among all toggles. In particular, the strong interaction and coupling condition eliminates spontaneous synchronization errors in terms of suppressing the emergence of undesired (9:1)-mixed states.

This result is similar to a well-known fact for oscillators coupled via a common medium that a transition from an unsynchronized to a synchronized regime emerges as the strength of coupling increases [15–17, 25, 56]. Indeed, many microbial species accomplish this via quorum sensing, which entails the secretion and detection of diffusible molecules (autoinducers), whose concentration serves as a proxy for population density [10].

Using the expression for the dimensionless parameter *a*_5_ given in Eqs (18) and (19), see Scaling, we can conclude that the increase in the values of the parameter *a*_5_ leading to the bifurcation point LP_2_ (Fig 4) can be achieved by the following experimental interventions:

- stabilization of cell division with lower values of the specific growth rate *μ* (or, equivalently, higher division periods *T*);
- stabilization of relevant proteins, using lower values of *r*_*d*_ (or, equivalently, higher half-lives);
- an increase in the maximum production rate (*k*_*G*_) of C14-HSL by enzyme CinI, see SI-3 Estimation of Parameter Values in S1 Text;
- an increase in the sensitivity (*K*_*G*_) of promoter P_cin_ with respect to the number of molecules C14-HSL to half-activate P_cin_, see Table SI-3.2 given in SI-3 Estimation of Parameter Values in S1 Text.

We have used bifurcation analysis with respect to changes in the values of the parameter *a*_5_ as a way to illustrate predictions from monotone systems theory, and in the process we obtained conclusions regarding improvements of S toggle self-synchronization properties by eliminating the (9:1)-mixed state. To this end, we note that there is no need to further increase values of *a*_5_ to move the system to the bifurcation point LP_1_ at which the (1:1)-mixed state loses it stability and disappears, because we do not interpret the (1:1)-mixed state as a spontaneous synchronization error, see Spontaneous Synchronization Errors. Additional parametric dependencies with respect to changes in other parameters are shown in Figs SI-7.1 and SI-7.1 in S1 Text.

**Fig 5:**
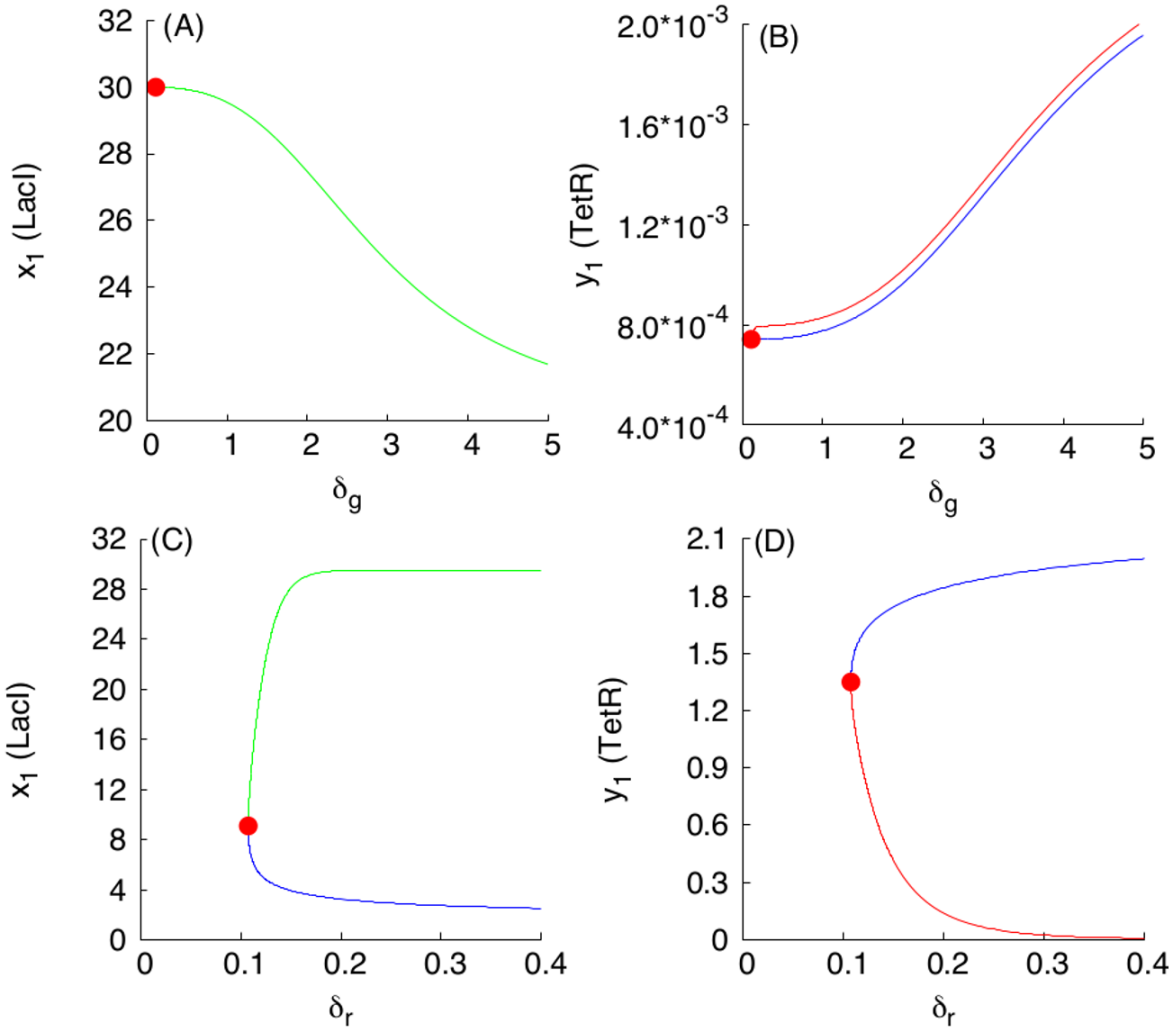
Examples of monotone parametric dependencies for a (1:1)-mixed state. Panels (A) and (B) correspond to dependencies of LacI and TetR levels on parameter *δ*_*g*_, respectively. Panels (C) and (D) correspond to dependencies of LacI and TetR levels on parameter *δ*_*r*_, respectively. The dependencies for the G-subpopulation are shown only, within which LacI is activated, while TetR is repressed. Green and red solid curves correspond to stable branches of (1:1)-equilibrium solutions, while all blue curves correspond to unstable branches of the solutions. Red filled circles correspond to an LP-bifurcation point. In panel (A), projections of stable and unstable branches coincide and, so, only the stable branch is shown.

We then repeat the analysis of the same parametric dependencies for a (1:1)-mixed state, illustrated in Fig 5 and Fig SI-7.3 in S1 Text. Like in the previous case, we observe that all dependencies are in line with the predictions suggested by Fig 3. To this end, we will not provide here reproduced similar results for the saturated S design (SI-8.1 Modification of the S Model in S1 Text), because in all computationally investigated cases, the parameter monotone dependencies are predicted by the theory and Fig 3.

The LP-bifurcation point (Fig 5) can be interpreted as follows. Decreasing values of both parameters *δ*_*g*_ and *δ*_*r*_ leads to an increase in the intracellular and extracellular levels of the corresponding QS signaling molecules, which, in turn, leads to stronger interactions among all toggles. Indeed, it follows from Eqs (20) and (21) (see Scaling) that the described changes in the values of dimensionless parameters *δ*_*g*_ and *δ*_*r*_ can be achieved by increasing half-lives of the corresponding QS signaling molecules.

To this end and similarly to the interpretation provided earlier, as the values of the parameters *δ*_*g*_ and *δ*_*r*_ decrease, the (1:1)-mixed state loses its stability and disappear via an LP-bifurcation (Fig 5), the effect which is similar to the well-known fact that oscillators coupled via common medium synchronize as the strength of coupling increases [15, 25, 56].

We note that the parametric dependencies for unstable solutions are not described by Fig 3. To explain this observation, we recall that our proof of monotone dependence on parameters applies to stable solutions only, see above.

Finally, the monotone parametric dependencies for (9:1)-mixed states corresponding to spontaneous synchronization errors are illustrated in Figs SI-7.4 and SI-7.5 in S1 Text. In this case, by increasing the strength of interactions between the toggles from the large subpopulation, the spontaneous error can also be eliminated, corresponding to the existence of the LP-points in panels (A) and (B) of Figs SI-7.4 and SI-7.5 in S1 Text. At the same time, increasing the strength of interactions between the toggles from the small population, the corresponding spontaneous error cannot be eliminated.

### Bistability in Single S and A Toggles

**Fig 6:**
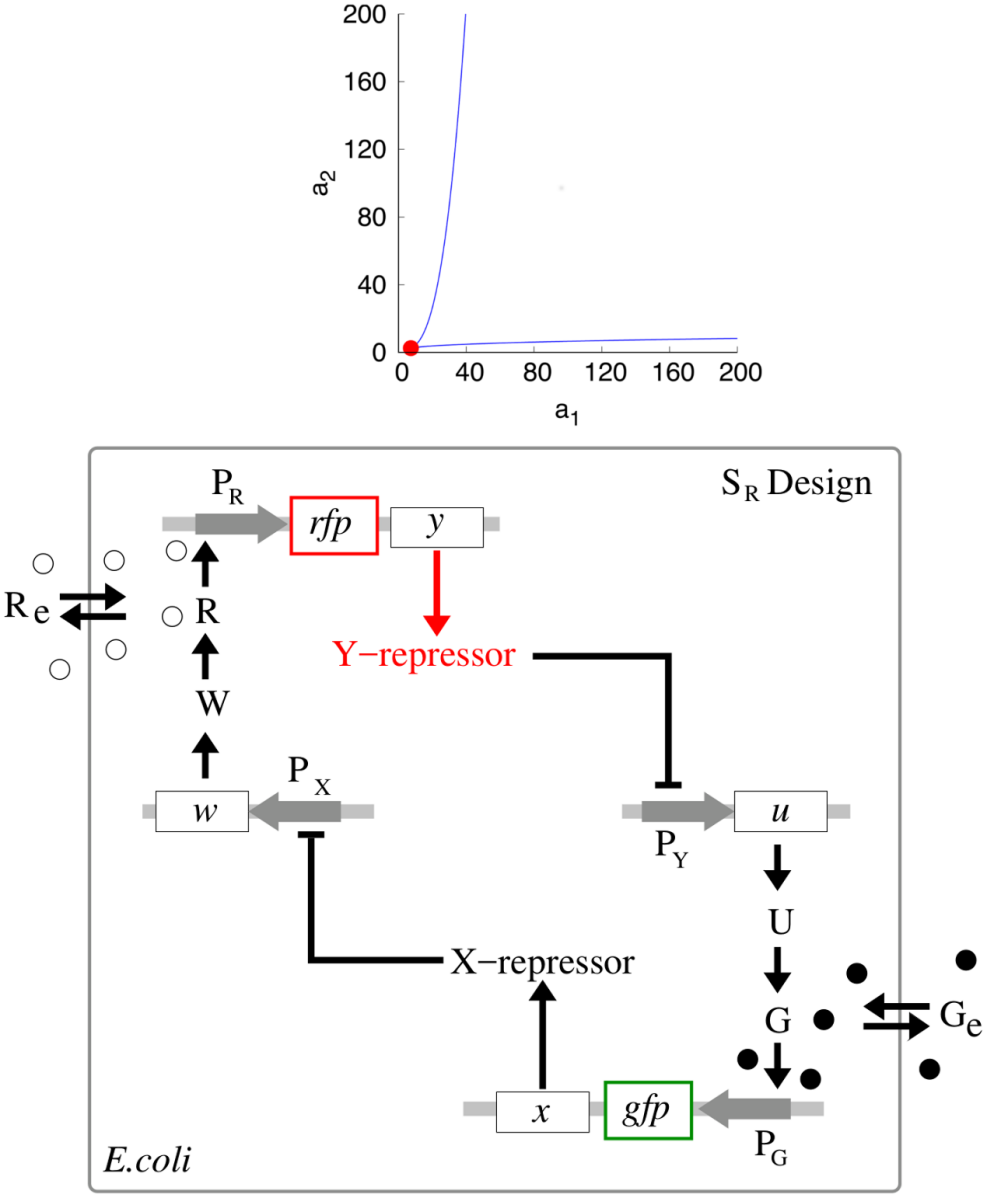
Bistability regions for S and A toggles (top), and a reduced S_R_ toggle design discovered from the bistability region (bottom). (Top panel). The region between two blue color coded LP-bifurcation loci corresponds to a bistability region for the A toggle model Eqs (9) and (10) at *d* = 0. A red filled circle corresponds to a cusp point (CP). For the S toggle model, bistability exists for all parameter values *a*_1_ ≥ 0 and *a*_2_ ≥ 0 at *d* = 0. Other fixed parameter values are given in Eqs (11)–(14). (Bottom panel). The reduced *S*_*R*_ toggle is obtained from the original S toggle (Fig 1) after removal of genes *lacI* and *tetR* from the corresponding plasmids bearing promoters P_Y_ and P_X_, respectively. This reduction procedure corresponds to setting zero values *a*_1_ = *a*_2_ = 0 as discussed in the main text.

Before comparing population properties of our S design to those of the A design, we remark that, even for isolated cells (when the diffusion constant *d* is zero), there is a larger range of bistability for the S design compared to the A design. Specifically, a bistability region for a single A toggle in the plane (*a*_1_, *a*_2_) at *d* = 0 is shown in Fig 6(top panel). Similar regions were found in [1, 16]. We also observe that the entire quadrant, *a*_1_ ≥ 0 and *a*_2_ ≥ 0, spans a bistability region for the S-model at the fixed parameter values given in Eqs (11)–(14). We have computed the bistablility regions for the S design for three different nonzero values of the promoter leakiness parameter *γ* = 0.01, 0.1, 1.0, respectively, while all other parameter values were kept fixed as in the reference set Eqs (11)–(14), and found that in all the three cases, the entire quadrant, *a*_1_ ≥ 0 and *a*_2_ ≥ 0, belongs to the computed regions. In contrast, the bistability region for the A design depends on the promoter leakiness parameter significantly [16], and we also observed computationally that the bistability region was leaving the domain shown in Fig 6 (top panel) as soon as *γ* was allowed to take on values larger than 0.5, that is, when *γ* > 0.5.

Another important observation that follows immediately from Fig 6 (top panel) is that in the case of the S toggle, bistability persists at the origin of the non-negative quadrant in the plane (*a*_1_, *a*_2_), that is, at *a*_1_ = *a*_2_ = 0. The observation remains true even for the nonzero values of the leakiness parameters as discussed earlier. Additionally, the property persists for the saturated S design (SI-8.1 Modification of the S Model in S1 Text) with the updated parameter set Eqs (15)–(17). This simply means that the genes *lacI* and *tetR* can be removed from the corresponding plasmids bearing promoters P_Y_ and P_X_, respectively (Fig 1). In this case (Fig 6) (bottom panel), it is enough to keep the genes on the plasmids bearing the corresponding promoters P_G_ and P_R_ (Fig 1). We view the reduced S toggle as a minimal design that could be implemented experimentally. The fuller construct S is interesting too, in so far as it is based on the well-characterized and studied Cantor-Collins switch, coupled to quorum-sensing components [4]. We find that the full and reduced designs do not differ much in performance, and, so, we do not consider the minimal design in the rest of the paper.

### Bistable Homogeneous Populations of S and A Toggles

Bistable homogeneous populations of S toggles persist within large ranges of the model parameters. For example, panels (A) and (B) in Fig SI-7.6 in S1 Text show scaled levels of LacI and C14-HSL, respectively, for a homogeneous population of S toggle in the G-state, depending on the values of the diffusion (membrane permeability) parameter *d*.

Panels (C)—(F) in Fig SI-7.6 in S1 Text show two stable homogeneous populations of A toggle which coexist while the parameter *d* is allowed to vary. Because the A toggle design does not have any intrinsic symmetry, the levels of the activated repressor proteins, LacI for the G-homogeneous population shown in panels (C) and (E), and TetR for the R-homogeneous population shown panels (D) and (F), differ significantly from one another. Recall that the levels of LacI and TetR in the corresponding G- and R-homogeneous populations consisting of S toggles are identically the same due to mirror symmetry.

Our intensive computational studies confirm that the discussed results on the stable homogeneous populations of S and A toggles, as well as their dependencies on the diffusion parameter *d*, are robust with respect to perturbations in the model parameters, including various combinations in the values of the Hill coefficients, promoter leakiness, and the saturation conditions (SI-8 Modification of the S and A Models to Describe Sequestration of AAA+ protease ClpXP in S1 Text).

The combination of the analyses discussed here can be summarized by saying that under each one of the two designs, S and A, including biological variability in the Hill coefficients, promoter leakiness, and the degradation sequestration conditions, bistable homogeneous stable populations are possible, in either “Red” or “Green” consensus states, and with the same order of magnitude of expression. The difference between these designs, including the sequestration effect for AAA+ proteases ClpXP, become evident, when we study heterogeneous (mixed) populations, as discussed next.

### Elimination of (1:1)-Mixed Populations of S Toggles

**Fig 7:**
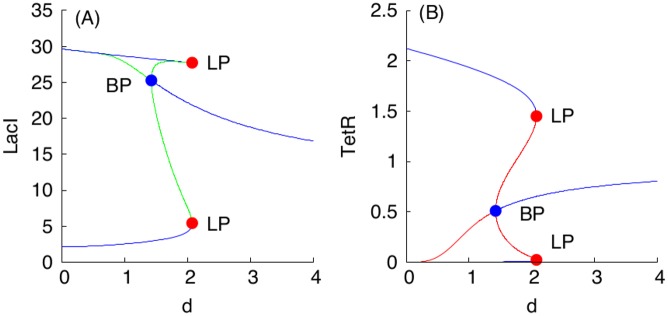
Symmetry breaking in a (1:1)-mixed population of S toggles. Panels (A) and (B) show the dependencies of LacI and TetR levels for the G-subpopulation of a (1:1)-population of S toggles, respectively. Blue color-coded plots correspond to all *unstable* equilibrium solution branches, while green and red color-coded plots correspond to all *stable* equilibrium solution branches. All blue filled BP-labeled points correspond to *d* ≈ 1.43. All red filled LP-labeled points correspond to *d* ≈ 2.07.

Fig 7 shows richness of dynamic effects (bifurcations) for a (1:1)-mixed population of S toggles. We see that as soon as the parameter *d* takes on larger values, the (1:1)-mixed state loses its stability via a Branch Point (BP) bifurcation [62] (alternatively called “pitchfork” or “symmetry-breaking” bifurcation [70, 71]), giving rise to two stable (1:1)-mixed non-symmetric states at *d* ≈ 1.43. The general symmetry-breaking phenomenon is rigorously studied in [72, 73].

**Fig 8:**
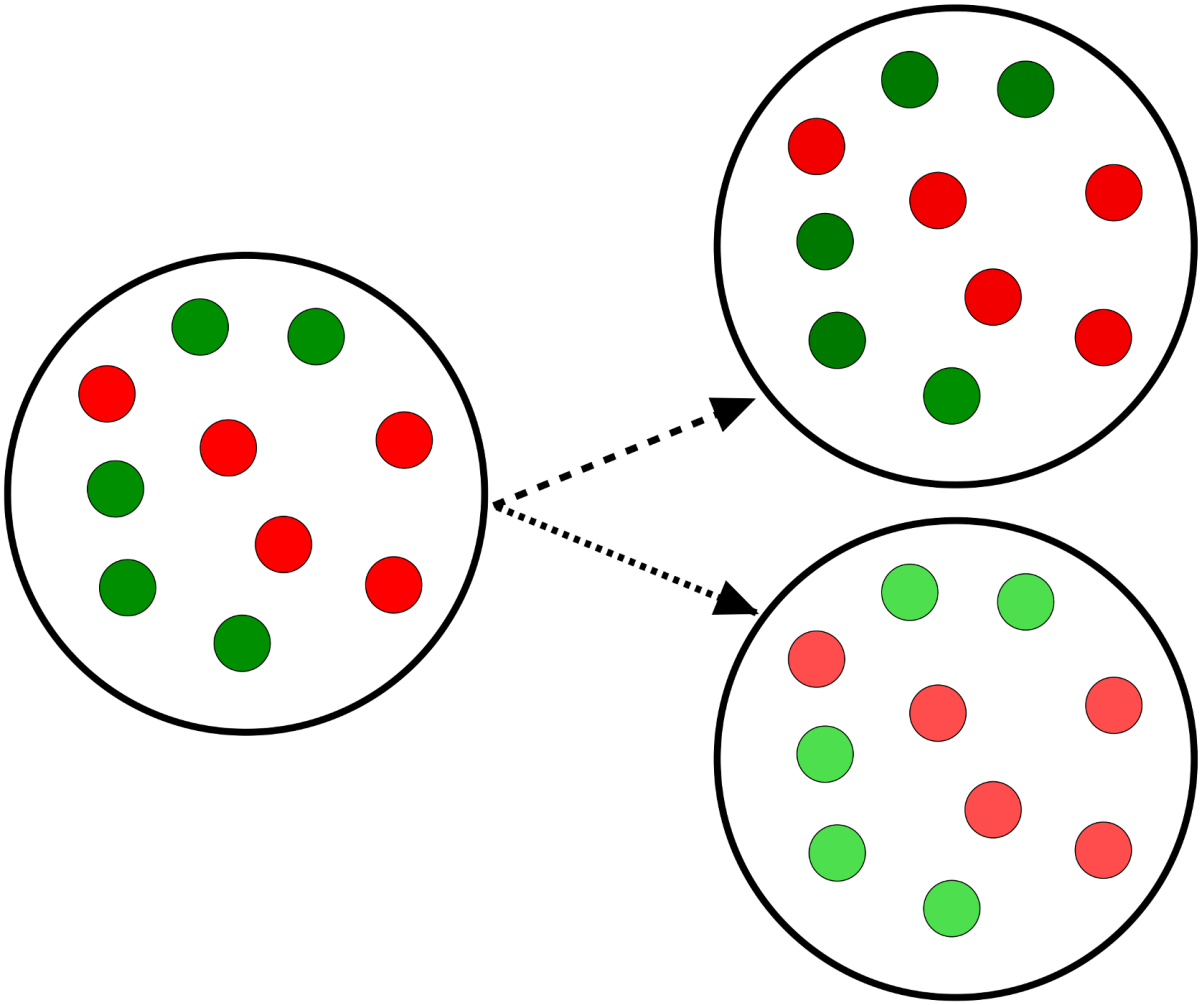
An interpretation of symmetry breaking in a (1:1)-mixed population of S toggles. A new (1:1)-asymmetric mixed state arises, in which there are two subpopulations, one in which *green* gene-expression *dominates* (but with different expression levels of LacI in each of them), and another one which *red* gene-expression *dominates* (also with different TetR levels).

The symmetry-breaking scenario can be described intuitively as follows. Suppose that we start with a mixed population in which 50% of the cells are in “green” state and 50% of the cells are in “red” state, and the nondimensional diffusion coefficient *d* (which, as we saw, in fact incorporates many of the kinetic parameters in the original system) has a low value. Suppose that we now slowly increase the value of *d*, and ask what happens to the (1:1)-mixed state. The first event that is observed, at *d* ≈ 1.43 corresponding to the BP points in all panels of Fig 7, is that this “pure 50–50 mixed state” loses its stability. A new mixed state arises (Fig 8), in which there are *two* subpopulations, one in which green gene-expression dominates (but with different expression levels of LacI in each of them), and another one which red gene-expression dominates (also with different TetR levels). These two mixed states correspond to the solution branches connecting points marked with labels BP and upper LP, and BP and lower LP, respectively, shown in all panels of Fig 7.

Furthermore, as *d* is increased a bit more (past *d* ≈ 2.07 corresponding to the two points labeled with LP in all panels of Fig 7, respectively), even these mixed states disappear (Fig 7). Thus, even with moderate diffusion, heterogeneous populations cannot be sustained, emphasizing the consensus-forming character of the S design. This is in marked contrast to the A design, as shown next. The loss of stability by the (1:1)-mixed state increases the robustness of the S toggle design towards its self-synchronization by reducing the number of alternative stable states to which the toggle state can settle.

### Robustness of (1:1)-Mixed Populations of A Toggles and Saturated S toggles

In contrast to (1:1)-mixed populations of (unsaturated) S toggles described by the S model Eqs (1)–(6), we observe from Fig SI-7.7 in S1 Text that the original (1:1)-mixed A-population cannot be eliminated (made unstable) by increasing the values of the parameter *d* within a very large parameter interval. In other words, increasing the strength of interactions between the cells does not help to establish synchronization across the given population of identical A toggles. This is in a total agreement with a similar observation reported in [16], where the A model is studied in great detail. Specifically, it is found that a strong interaction between A toggles (*e.g.*, high permeability of the membrane to the autoinducer similar to higher values of *d*) results in the suppression of synchronous oscillations, leading to a transition of the population to a stable heterogeneous state, where individual A toggles are locked in different equilibrium states.

Our computational experiments with (1:1)-mixed populations of (saturated) S_m_ toggles described by the S_m_ model (SI-8.1) (see SI-8.1 Modification of the S Model in S1 Text) led to dependencies qualitatively indistinguishable from those shown in Fig SI-7.7 in S1 Text. Therefore, we can conclude that the degradation saturation (sequestration) effect may prevent the elimination of the undesired mixed states and synchronization.

### (9:1)-Mixed Population of S Toggles

**Fig 9:**
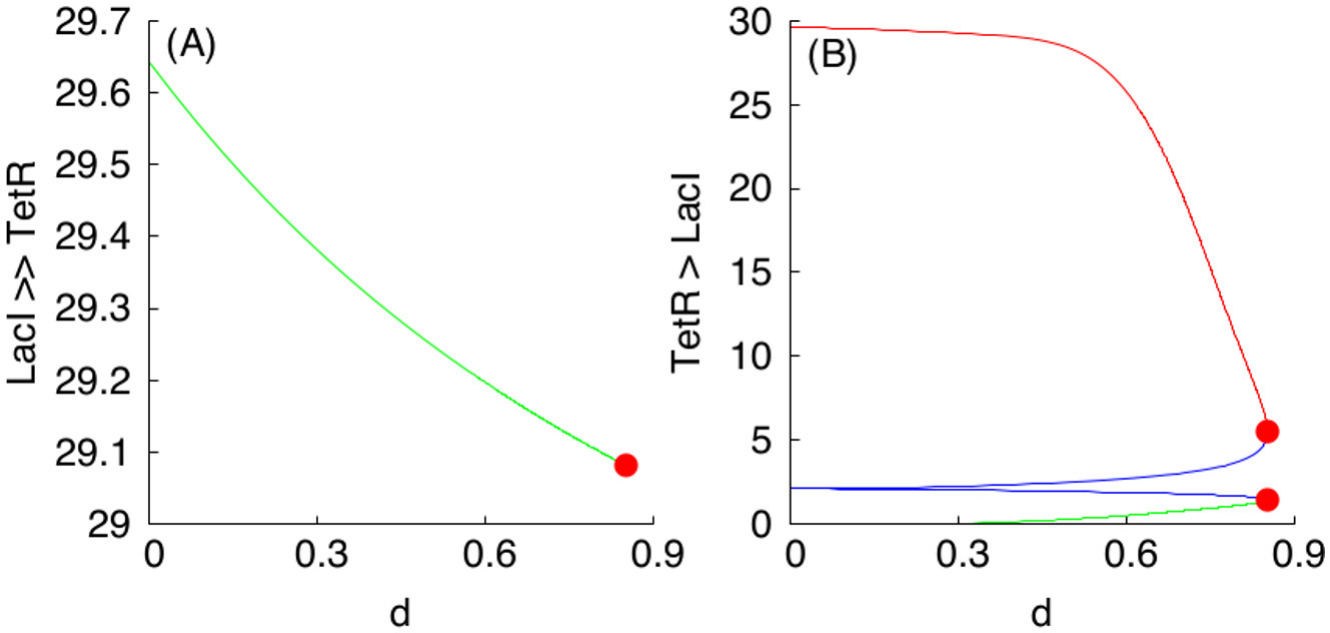
(9:1)-mixed population of S toggles. Panels (A) and (B) correspond to large and small subpopulations of a (9:1)-population of S toggles. All notations and color-coding schemes are as in Fig 7. Red filled circles correspond to the same LP-bifurcation point. In panel (A), projections of stable and unstable solution branches overlap. Because TetR is totally suppressed in the large (90%) subpopulation, the levels of TetR are not shown. Contrarily to panel (A), both TetR and LacI levels are plotted in panel (B) since LacI is only moderately suppressed in the small (10%) R-subpopulation.

Next, we consider bistable (9:1)-mixed populations of S Toggles, which as discussed in the introduction, we think of as arising from random synchronization errors. We observe that (9:1)-populations of S toggles become quickly extinct as soon as the values of the nondimensional diffusion parameter *d* are slightly increased (Fig 9).

### Robustness of (9:1)- and (1:9)-Mixed Populations of A Toggles and Saturated S Toggles

In contrast to the S design, in the A design, the mixed (9:1)- and (1:9)-heterogeneous populations that might arise from random state switching cannot be eliminated by changes in the values of the parameter *d* (Fig SI-7.8 in S1 Text). This is again in a total agreement with a similar observation reported in [16].

Using simulations carried out with the S_m_ model (SI-8.1 Modification of the S Model in S1 Text), we also observed that the sequestration effect results in stable (9:1)-mixed states for the S design existing for large ranges of the diffusion parameter *d*.

### Probing Capabilities of the S Toggle Design for Self-Correction of Spontaneous Synchronization Errors

To probe and compare capabilities of the S toggle and A toggle designs to correct “spontaneous synchronization errors” caused by a random flip of one toggle (or a small fraction of toggles) from a homogeneous population to the state opposite to the transcription signature adopted by the majority of the cells, we have performed simple random tests. In mathematical and computational terms, these random tests can be interpreted as an elementary numerical procedure to evaluate the size of the basin of attraction for the corresponding equilibrium solutions by sampling the corresponding small neighborhoods of the solutions, using random initial conditions, for each parameter value *d* ∈ {0.01, 10, 100} as follows, (1) find stable G- and R-homogeneous states (for any population size!), (2) flip 10% of population, and (3) explore initial conditions in neighborhood of this state value for the corresponding state variable (for the S design, since symmetric, only the G-homogeneous state needs to be explored).

**Fig 10:**
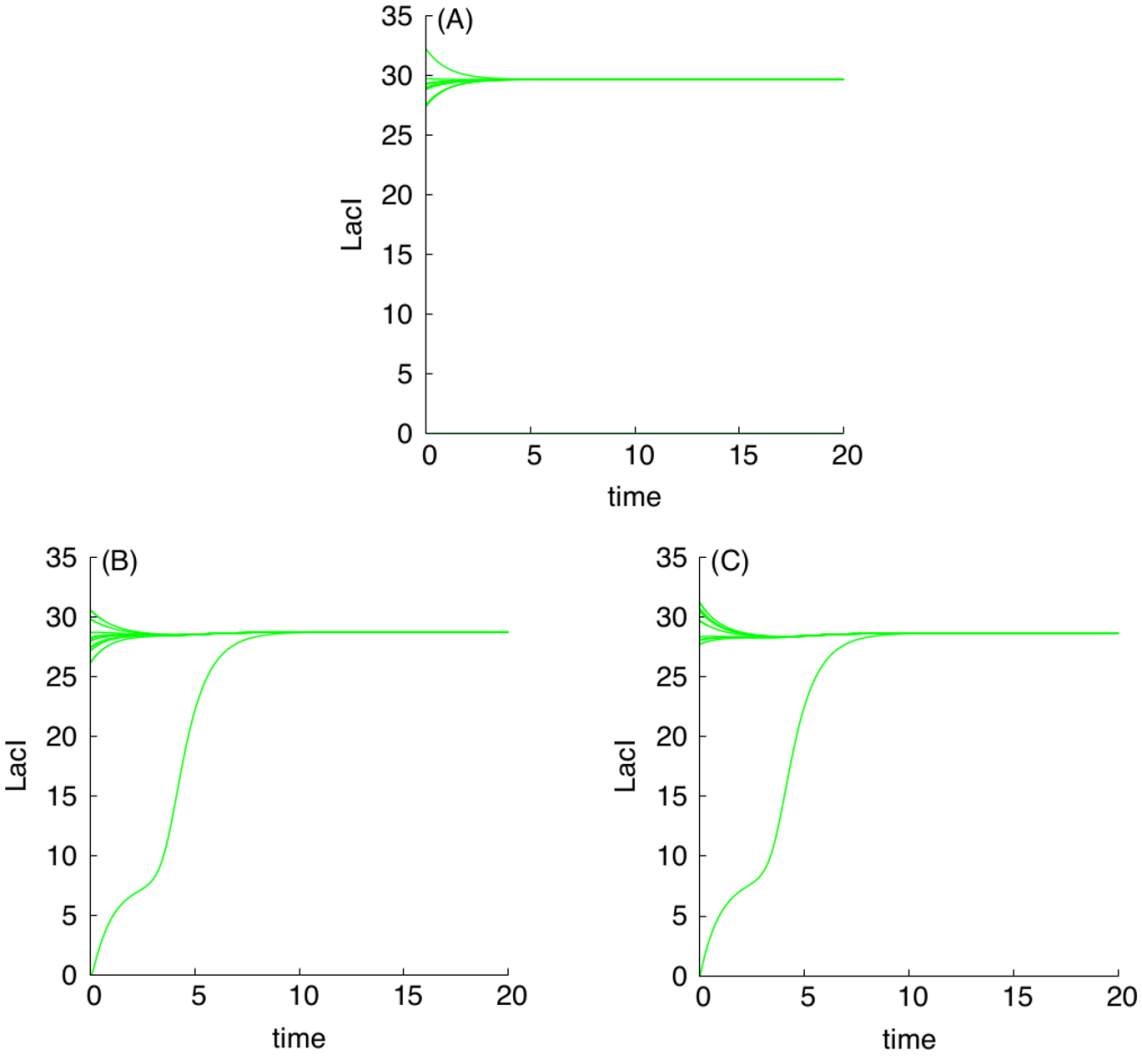
Self-correction of spontaneous errors by S toggles. **Panel (A)** shows the S toggle which cannot self-correct a (9:1)-spontaneous synchronization error for a small value of the diffusion parameter *d* = 0.01 (a weak coupling between all cells). **Panels (B) and (C)** show the S toggle which can self-correct a (9:1)-spontaneous synchronization error for a medium (*d* = 10) and large (*d* = 100) values of parameter *d* (a medium and strong coupling between all cells, respectively). For the values of parameter *d* used in Panels (B) and (C), the mixed states become unstable, see Fig 9.

We can conclude from Fig SI-7.9 in S1 Text that the A toggle does not have any capability for self-correction of spontaneous errors for all tested values of the parameter *d* (Fig SI-7.9 in S1 Text). The S toggle can self-correct spontaneous synchronization errors for the medium and large values of the parameter *d* (Fig 10) for all parameters values for which the mixed state becomes unstable, see Fig 9 ((9:1)-Mixed Population of S Toggles.) The rate of the error correction can be to some extend characterized by the observation that the error is corrected within the first 10 minutes counted from its onset (Fig 10). Unfortunately, theory does not preclude damped oscillations. Thus, all we can do is to make computational estimates in realistic parameter ranges.

To check how the limited availability of AAA+ proteases ClpXP may negatively impact the self-correctness property by the S design, we have developed an additional S_m_ model describing saturation (sequestration) of the AAA+ proteases ClpXP (SI-8.1 Modification of the S Model in S1 Text.) We then conducted additional computations to show that the sequestration, while preserving monotonicity and bistability properties, can lead to the loss of the self-correction of spontaneous errors by the S design. Thus, high levels of these proteases are required to implement successfully the S design.

Finally, we note that the reported results on the (9:1)-mixed states for both S and A designs are independent of the number *N* of cells in the given population with density *ρ* and can be applied to any population consisting of thousands or even millions of cells, split into two subpopulations comprising 90% and 10% fractions of all cells with different transcription signatures, respectively (Stability and Bifurcation in Cellular Populations.) Specifically, if the given (9:1)-mixed state is unstable for the S design in the model of 10 (identical) cells, it will be unstable in the model describing a larger population of (identical) cells because its stability is determined form an auxiliary system of *four* cells only (SI-6 Exponential Stability of Cellular Populations in S1 Text.) The same is true for the stable (9:1)-mixed state for the A design.

### Conclusion

In this study, we have shown how synthetic bistable circuits (toggles), and hosting them, programmable cellular populations, can be designed so as to solve a robust molecular task, the maintenance of a coordinated state, and a “majority-vote” auto-correction of deviations, of a binary switch. Our design was guided by concepts from monotone systems theory [18–23]. Specifically, we have shown how this concept can be used for the design of a new class of monotone synthetic biological toggles, including predictive capabilities describing both dynamic state variables and monotone parametric tendencies caused by parameter perturbations.

To benchmark the new toggle design, termed the S design, and the monotone systems approach, we have compared the S design with the known (and non-monotone) B2-strain from [4], termed the asymmetric or A design in this work. The B2-strain has been previously studied both experimentally [4] and theoretically [16, 17]. Despite a number of remarkable properties of the B2-strain (A design), the A toggle multifunctionality suggests that the design must be tightly controlled to avoid spontaneous switching not only between different expression states, but, as well, between different functions such as a bistable memory and an oscillatory phenotype.

In this respect, modern gene therapy interventions are currently limited to transfected genes to be either in an “on” or “off” state, when the expression of the transfected gene needs to be regulated tightly for the effective treatment of many diseases. To address this challenge, the monotone S toggle design completely excludes any unpredictable chaotic behaviors, as well as undesired stable oscillations. This conclusion is valid (of course, under certain experimentally controllable conditions pointed out in this work) for all parameter values, and provides a strong theoretical guarantee missing from other synthetic biology designs. Some of conditions include: (*i*) a reduced promoter leakiness [51], and (*ii*) unsaturated levels of AAA+ proteases ClpXP.

To achieve an in-depth understanding of dynamic properties of the S toggle design, we have developed biochemically-detailed and biologically-relevant mathematical models to test predictions of monotone systems theory by employing computational bifurcation analysis. To have all results biologically grounded, concrete molecular entities have been used, though the results are general and independent of any specific details.

To investigate the effect of a spontaneous toggle switching within cellular populations, leading to bimodal distributions, we have formalized a concept of spontaneous synchronization errors and tested the toggle design capabilities to self-correct spontaneous synchronization errors by sampling the basin of attraction of the corresponding equilibrium solutions. We found that the S toggle design was able to self-correct (or, auto-correct) synchronization errors, while the non-monotone A toggle design was not.

Because the number of cells in populations is *a priori* unknown, all the above results and conclusions can make sense only if they are made independently of the population size. To justify the above assertion, we have proved a few general theorems on the exponential stability of the equilibrium solutions corresponding to both homogeneous and mixed populations. The simple exponential stability results are independent of the number of cells in the populations and are based on basic first principles of stability analysis resulting from the Schur’s formula [63], allowing the characteristic polynomials for the corresponding model linearizations to be computed explicitly.

Using an additional model describing saturation (and sequestration) of AAA+ proteases ClpXP (SI-8 Modification of the S and A Models to Describe Sequestration of AAA+ protease ClpXP in S1 Text), we have observed computationally that even when the above-mentioned conditions (*i*) and (*ii*) are violated, the S toggle still demonstrates the monotonicity properties. If proteases are in limited supply, however, the conclusions break down, because of the non-monotonicity arising from resource competition. Thus, an important consideration when practically implementing our design is to express these proteases at a high enough level.

We remark that our design is based on a bistable design based on deterministic models. This approach is normally used in synthetic biology design of toggle switches, and our goal was to employ ready technology. On the other hand, in gene regulatory networks bistability, or, to be more precise, multi-modality of steady state distributions, may arise in deterministically monostable systems due to low molecule number effects. Intuitively, a slow switch between two promoter states (modeled in simplest terms by a two-state Markov chain) gives rise to a “bimodal” distribution of gene activation (gene is “on” or “off”); this process then may drive a large-molecule number mRNA and protein process, in effect creating a bimodal protein distribution, though this bimodality would be “averaged out” in a deterministic model that considers a large population. In this context, one may mention the work by Thomas et al. [74] which provides a system with two mutually repressing promoters using noncooperative transcriptional regulation but supplemented by a translational control component in which the protein product of one gene binds and degrades the mRNA of the other gene. Because we use cooperative binding (Hill coefficients 2 and larger), our design is specifically geared to bistability even in low noise situations, and the engineered consensus mechanism is designed to correct for noise-induced transitions. It would be interesting in further work to study consensus designs for toggles based on stochastic bimodality.

## Supporting Information

S1 Text Supplemental Information (SI) materials S1–S8. SI-1 Toggle B2. SI-2 Model Derivation. SI-3 Estimation of Parameter Values. SI-4 Alternative Definitions of Monotone Systems and Order Preservation. SI-5 Symmetry. SI-6 Exponential Stability of Cellular Populations. SI-7 Additional Figures. SI-8 Modification of the S and A Models to Describe Sequestration of AAA+ Protease ClpXP.
(PDF)

## References

[1] Gardner TS, Cantor CR, Collins JJ. Construction of a genetic toggle switch in *Escherichia coli*. Nature. 2000 1;403(6767):339–342. 10.1038/35002131 10659857

[2] Elowitz MB, Leibler S. A synthetic oscillatory network of transcriptional regulators. Nature. 2000 1;403(6767):335–338. 10.1038/35002125 10659856

[3] Hasty J, Pradines J, Dolnik M, Collins JJ. Noise-based switches and amplifiers for gene expression. Proc. Natl. Acad. Sci. U.S.A. 2000 2;97(5):2075–80. 10.1073/pnas.040411297 10681449 PMC15756

[4] Kobayashi H, Kærn M, Araki M, Chung K, Gardner TS, Cantor CR, Collins JJ. Programmable cells: interfacing natural and engineered gene networks. Proc. Natl. Acad. Sci. U.S.A. 2004 6;101(22):8414–8419. 10.1073/pnas.0402940101 15159530 PMC420408

[5] Purnick PE, Weiss R. The second wave of synthetic biology: from modules to systems. Nat. Rev. Mol. Cell Biol. 2009 6;10(6):410–422. 10.1038/nrm2698 19461664

[6] Arpino JA, Hancock EJ, Anderson J, Barahona M, Stan GB, Papachristodoulou A, Polizzi K. Tuning the dials of synthetic biology. Microbiology. 2013 7;159(7):1236–1253. 10.1099/mic.0.067975-0 23704788 PMC3749727

[7] Daniel R, Rubens JR, Sarpeshkar R, Lu TK. Synthetic analog computation in living cells. Nature. 2013 5;497(7451):619–623. 10.1038/nature12148 23676681

[8] Carbonell-Ballestero M, Duran-Nebreda S, Montañez R, Solé R, Macía J, Rodríguez-Caso C. A bottom-up characterization of transfer functions for synthetic biology designs: lessons from enzymology. Nucleic Acids Res. 2014 11:gku964. 10.1093/nar/gku964 PMC4267673 25404136

[9] Chen Y, Kim JK, Hirning AJ, Josić K, Bennett MR. Emergent genetic oscillations in a synthetic microbial consortium. Science. 2015 8;349(6251):986–989. 10.1126/science.aaa3794 26315440 PMC4597888

[10] Nadell CD, Bassler BL, Levin SA. Observing bacteria through the lens of social evolution. J. Biol. 2008 9;7(7):1–27. 10.1186/jbiol87 18831723 PMC2776406

[11] Miller MB, Bassler BL. Quorum sensing in bacteria. Annu. Rev. Microbiol. 2001 10;55(1):165–199. 10.1146/annurev.micro.55.1.165 11544353

[12] Pesci EC, Pearson JP, Seed PC, Iglewski BH. Regulation of *las* and *rhl* quorum sensing in *Pseudomonas aeruginosa*. J. Bacteriol. 1997 5;179(10):3127–3132. 9150205 10.1128/jb.179.10.3127-3132.1997 PMC179088

[13] Lithgow JK, Wilkinson A, Hardman A, Rodelas B, Wisniewski-Dyé F, Williams P, Downie JA. The regulatory locus cinRI in *Rhizobium leguminosarum* controls a network of quorum-sensing loci. Mol. Microbiol. 2000 7;37(1):81–97. 10.1046/j.1365-2958.2000.01960.x 10931307

[14] McAnulla C, Edwards A, Sanchez-Contreras M, Sawers RG, Downie JA. Quorum-sensing-regulated transcriptional initiation of plasmid transfer and replication genes in *Rhizobium leguminosarum biovar viciae*. Microbiol. 2007 7;153(7):2074–2082. 10.1099/mic.0.2007/007153-0 17600052

[15] Garcia-Ojalvo J, Elowitz MB, Strogatz SH. Modeling a synthetic multicellular clock: repressilators coupled by quorum sensing. Proc. Natl. Acad. Sci. U.S.A. 2004 7;101(30):10955–10960. 10.1073/pnas.0307095101 15256602 PMC503725

[16] Kuznetsov A, Kærn M, Kopell N. Synchrony in a population of hysteresis-based genetic oscillators. SIAM J. Appl. Math. 2004;65(2):392–425. 10.1137/S0036139903436029

[17] Wang J, Zhang J, Yuan Z, Zhou T. Noise-induced switches in network systems of the genetic toggle switch. BMC Syst. Biol. 2007 11;1(1):50 10.1186/1752-0509-1-50 18005421 PMC2214838

[18] Smale S. On the differential equations of species in competition. J Math. Biol. 1976 3;3(1):5–7. 10.1007/BF00307854 1022822

[19] Hirsch MW. The dynamical systems approach to differential equations. Bull. A.M.S. 1984;11(1):1–64. 10.1090/S0273-0979-1984-15236-4

[20] Angeli D, Sontag ED. Monotone control systems. IEEE Trans. Automat. Control. 2003;48(10):1684–1698. 10.1109/TAC.2003.817920

[21] Hirsch M, Smith H. Monotone dynamical systems Handbook of differential equations: Ordinary differential equations. Amsterdam: Elsevier BV 2005;2:239–357.

[22] Sontag ED. Monotone and near-monotone biochemical networks. Syst. and Synth. Biol. 2007;1:59–87. 10.1007/s11693-007-9005-9 19003437 PMC2533521

[23] Smith HL. Monotone dynamical systems: An introduction to the theory of competitive and cooperative systems Mathematical Surveys and Monographs. Vol. 41 Providence, RI: A.M.S 2008.

[24] Parsek MR, Greenberg EP. Acyl-homoserine lactone quorum sensing in gram-negative bacteria: a signaling mechanism involved in associations with higher organisms. Proc. Natl. Acad. Sci. U.S.A. 2000 8 1;97(16):8789–8793. 10.1073/pnas.97.16.8789 10922036 PMC34013

[25] Resmi V, Ambika G, Amritkar RE. Synchronized states in chaotic systems coupled indirectly through a dynamic environment. Phys. Rev. E. 2010 4;81(4):046216 10.1103/PhysRevE.81.046216 20481816

[26] Sontag ED. Mathematical control theory: deterministic finite dimensional systems. New York: Springer Science & Business Media 2013.

[27] Parsek MR, Val DL, Hanzelka BL, Cronan JE, Greenberg EP. Acyl homoserine-lactone quorum-sensing signal generation. Proc. Natl. Acad. Sci. U.S.A. 1999 4;96(8):4360–4365. 10.1073/pnas.96.8.4360 10200267 PMC16337

[28] Hooshangi S, Thiberge S, Weiss R. Ultrasensitivity and noise propagation in a synthetic transcriptional cascade. Proc. Natl. Acad. Sci. U.S.A. 2005 3;102(10):3581–3586. 10.1073/pnas.0408507102 15738412 PMC552778

[29] Tuttle LM, Salis H, Tomshine J, Kaznessis YN. Model-driven designs of an oscillating gene network. Biophys. J. 2005 12;89(6):3873–3883. 10.1529/biophysj.105.064204 16183880 PMC1366954

[30] Stricker J, Cookson S, Bennett MR, Mather WH, Tsimring LS, Hasty J. A fast, robust and tunable synthetic gene oscillator. Nature. 2008 11;456(7221):516–519. 10.1038/nature07389 18971928 PMC6791529

[31] Hussain F, Gupta C, Hirning AJ, Ott W, Matthews KS, Josić K, Bennett MR. Engineered temperature compensation in a synthetic genetic clock. Proc. Natl. Acad. Sci. U.S.A. 2014 1;111(3):972–977. 10.1073/pnas.1316298111 24395809 PMC3903251

[32] Laffend L, Shuler ML. Structured model of genetic control via the *lac* promoter in *Escherichia coli*. Biotechnol. Bioeng. 1994 3;43(5):399–410. 10.1002/bit.260430508 18615723

[33] Lutz R, Bujard H. Independent and tight regulation of transcriptional units in *Escherichia coli* via the LacR/O, the TetR/O and AraC/I1-I2 regulatory elements. Nucleic Acids Res. 1997 3;25(6):1203–1210. 10.1093/nar/25.6.1203 9092630 PMC146584

[34] Baumeister R, Flache P, Melefors Ö, von Gabain A, Hillen W. Lack of a 5’non-coding region in Tn 1721 encoded *tetR* mRNA is associated with a low efficiency of translation and a short half-life in *Escherichia coil*. Nucleic Acids Res. 1991 9;19(17):4595–4600. 10.1093/nar/19.17.4595 1653948 PMC328697

[35] Markiewicz P, Kleina LG, Cruz C, Ehret S, Miller JH. Genetic studies of the lac repressor. XIV. Analysis of 4000 altered Escherichia coli lac repressors reveals essential and non-essential residues, as well as “spacers” which do not require a specific sequence. J. Mol. Biol. 1994 7;240(5):421–33. 10.1006/jmbi.1994.1458 8046748

[36] Ramos JL, Martínez-Bueno M, Molina-Henares AJ, Terán W, Watanabe K, Zhang X, Gallegos MT, Brennan R, Tobes R. The TetR family of transcriptional repressors. Microbiol. Mol. Biol. Rev. 2005 6;69(2):326–56. 10.1128/MMBR.69.2.326-356.2005 15944459 PMC1197418

[37] Semsey S, Jauffred L, Csiszovszki Z, Erdőssy J, Stéger V, Hansen S, Krishna S. The effect of LacI autoregulation on the performance of the lactose utilization system in *Escherichia coli*. Nucleic Acids Res. 2013 5:gkt351. 10.1093/nar/gkt351 PMC3711431 23658223

[38] Gray KM, Garey JR. The evolution of bacterial LuxI and LuxR quorum sensing regulators. Microbiology. 2001 8;147(8):2379–2387. 10.1099/00221287-147-8-2379 11496014

[39] Shuler ML, Leung S, Dick CC. A mathematical model for the growth of a single bacterial cell. Ann. N Y Acad. Sci. 1979 5;326(1):35–52. 10.1111/j.1749-6632.1979.tb14150.x

[40] Neidhardt FC, Ingraham JL, Schaechter M. Physiology of the Bacterial Cell: A Molecular Approach. Massachusetts: Sinauer Associates, Inc., Publishers Sunderland 1990.

[41] Kim BG, Shuler ML. A structured, segregated model for genetically modified *Escherichia coli* cells and its use for prediction of plasmid stability. Biotechnol. Bioeng. 1990 9;36(6):581–92. 10.1002/bit.260360605 18595116

[42] Shu J, Shuler ML. Prediction of effects of amino acid supplementation on growth of *E. coli* B/r. Biotechnol. Bioeng. 1991 4;37(8):708–15. 10.1002/bit.260370804 18600666

[43] Laffend L, Shuler ML. Ribosomal protein limitations in *Escherichia coli* under conditions of high translational activity. Biotechnol. Bioeng. 1994 3;43(5):388–98. 10.1002/bit.260430507 18615722

[44] Bailey JE. Mathematical modeling and analysis in biochemical engineering: past accomplishments and future opportunities. Biotechnol. Prog. 1998 1;14(1):8–20. 10.1021/bp9701269 9496667

[45] Domach MM, Leung SK, Cahn RE, Cocks GG, Shuler ML. Computer model for glucose-limited growth of a single cell of *Escherichia coli* B/r-A. Biotechnol. Bioeng. 2000 3 20;67(6):827–40. 10.1002/(SICI)1097-0290(20000320)67:6%3C827::AID-BIT18%3E3.0.CO;2-N 10699861

[46] Nikolaev EV, Burgard AP, Maranas CD. Elucidation and structural analysis of conserved pools for genome-scale metabolic reconstructions. Biophys. J. 2005 1;88(1):37–49. 10.1529/biophysj.104.043489 15489308 PMC1305013

[47] Nikolaev EV, Atlas JC, Shuler ML. Computer models of bacterial cells: from generalized coarse-grained to genome-specific modular models. J. Phys.: Conf. Ser. 2006; 46(1):322–326.

[48] Atlas JC, Nikolaev EV, Browning ST, Shuler ML. Incorporating genome-wide DNA sequence information into a dynamic whole-cell model of *Escherichia coli*: application to DNA replication. IET Syst. Biol. 2008 9;2(5):369–382. 10.1049/iet-syb:20070079 19045832

[49] Nikolaev EV. The elucidation of metabolic pathways and their improvements using stable optimization of large-scale kinetic models of cellular systems. Metab. Eng. 2010 1;12(1):26–38. 10.1016/j.ymben.2009.08.010 19733253

[50] Lodish H, Berk A, Kaiser C, Krieger M., Bretscher A, Ploegh H., Amon A, Scott M. Molecular cell biology. New York: Macmillan 2012

[51] Penumetcha P, Lau K, Zhu X, Davis K, Eckdahl TT, Campbell AM. Improving the Lac system for synthetic biology. Bios. 2010 3;81(1):7–15. 10.1893/011.081.0104

[52] Stephanopoulos G, Aristidou AA, Nielsen J. Metabolic engineering: principles and methodologies. New York: Academic press 1998.

[53] Dockery JD, Keener JP. A mathematical model for quorum sensing in *Pseudomonas aeruginosa*. Bull. Math. Biol. 2001 1;63(1):95–116. 10.1006/bulm.2000.0205 11146885

[54] Bressloff PC. Stochastic processes in cell biology. New York: Springer 2014.

[55] Dong YH, Wang LH, Xu JL, Zhang HB, Zhang XF, Zhang LH. Quenching quorum-sensing-dependent bacterial infection by an N-acyl homoserine lactonase. Nature. 2001 6;411(6839):813–817. 10.1038/35081101 11459062

[56] Waldherr S, Allgower F. Network-level dynamics of diffusively coupled cells. Decision and Control (CDC), 2012 IEEE 51st Annual Conference 2012; 5517–5522.

[57] Farrell CM, Baker TA, Sauer RT. Altered specificity of a AAA+ protease. Mol. cell. 2007 1;25(1):161–166. 10.1016/j.molcel.2006.11.018 17218279 PMC1847774

[58] Khibnik AI, Kuznetsov YA, Levitin VV, Nikolaev EV. Continuation techniques and interactive software for bifurcation analysis of ODEs and iterated maps. Phys. D: Nonlinear Phenomena. 1993 1;62(1–4):360–71. 10.1016/0167-2789(93)90294-B

[59] Govaerts W, Kuznetsov YA, Dhooge A. Numerical continuation of bifurcations of limit cycles in MATLAB. SIAM J. Sci. Comput. 2005;27(1):231–252. 10.1137/030600746

[60] Doedel EJ. Numerical Continuation Methods for Dynamical Systems Lecture notes on numerical analysis of nonlinear equations. Netherlands:Springer 2007:1–49.

[61] Bindel D, Friedman M, Govaerts W, Hughes J, Kuznetsov YA. Numerical computation of bifurcations in large equilibrium systems in MATLAB. J. Comput. App. Math. 2014 5;261:232–48. 10.1016/j.cam.2013.10.034

[62] Kuznetsov YA. Elements of applied bifurcation theory. New York: Springer Science & Business Media 2013.

[63] Gantmakher FR. The theory of matrices. A.M.S.: Chelsea Publishing Company 1959.

[64] Hirsch MW. Differential equations and convergence almost everywhere in strongly monotone semiflows. Contemp. Math. 1983;17:267–285. 10.1090/conm/017/706104

[65] Hirsch MW. Systems of differential equations that are competitive or cooperative II: Convergence almost everywhere. SIAM J. Math. Anal. 1985 5;16(3):423–439. 10.1137/0516030

[66] Hadeler KP, Glas D. Quasimonotone systems and convergence to equilibrium in a population genetic model. J. Math. Anal. Applic. 1983 9;95(2):297–303. 10.1016/0022-247X(83)90108-7

[67] Zaslavsky T. A mathematical bibliography of signed and gain graphs and allied ares. Electronic Journal of Combinatorics 1998; DS8

[68] Dhooge A, Govaerts W, Kuznetsov YA. MATCONT: a MATLAB package for numerical bifurcation analysis of ODEs. ACM Transactions on Mathematical Software (TOMS). 2003 6;29(2):141–64. 10.1145/779359.779362

[69] Wang L, Sontag ED. Singularly perturbed monotone systems and an application to double phosphorylation cycles. J. Nonlin. Sci. 2008 10;18(5):527–50. 10.1007/s00332-008-9021-2

[70] Golubitsky M, Stewart I. Singularities and groups in bifurcation theory. New York: Springer Science & Business Media 2012.

[71] Nikolaev EV. Bifurcations of limit cycles of differential equations admitting an involutive symmetry. Sbornik: Mathematics. 1995;186(4):611 10.1070/SM1995v186n04ABEH000033

[72] Nikolaev E, Shnol E. Bifurcations of cycles in systems of differential equations with a finite symmetry group-I. J. Dynamic. Cont. Syst. 1998;4(3):315–341. 10.1023/A:1022832331959

[73] Nikolaev E, Shnol E. Bifurcations of cycles in systems of differential equations with a finite symmetry group-II. J. Dynamic. Cont. Syst. 1998;4(3):343–363. 10.1023/A:1022884316030

[74] Thomas P, Popović N, Grima R. Phenotypic switching in gene regulatory networks. Proc. Natl. Acad. Sci. U.S.A. 2014;111(19):6994–6999. 10.1073/pnas.1400049111 24782538 PMC4024914

