# Supplementary material for: Quorum-Sensing Synchronization of Synthetic Toggle Switches: A Design Based on Monotone Dynamical Systems Theory
Source: PLoS Comput Biol. 2016 Apr 29;12(4):e1004881. doi: 10.1371/journal.pcbi.1004881 (PMC4851387; doi:10.1371/journal.pcbi.1004881)
Supplement: S1 Text — SI-1 Toggle B2. SI-2 Model Derivation. SI-3 Estimation of Parameter Values. SI-4 Alternative Definitions of Monotone Systems and Order Preservation. SI-5 Symmetry. SI-6 Exponential Stability of Cellular Populations. SI-7 Additional Figures. SI-8 Modification of the S and A Models to Describe Sequestration of AAA+ Protease ClpXP. (PDF) [file pcbi.1004881.s001.pdf]

Quorum-Sensing Synchronization of Synthetic Toggle Switches: A Design based on Monotone Dynamical Systems Theory

Evgeni V. Nikolaev, Eduardo D. Sontag*

Department of Mathematics and Center for Quantitative Biology, Rutgers, The State University of New Jersey, Piscataway, NJ 08854-8019, The United States

*Corresponding Author: eduardo.sontag@rutgers.edu

Supporting Information

SI-1 Toggle B2

Kobayashi et al [1] consider a number of genetic toggle switches, interfaced with a QS signaling pathway. Specifically, their *E. coli* strain "B2" (Fig. SI-1.1) detects as well as produces (through the synthetase encoded by the expressed gene luxI, which converts common precursor metabolites) acyl-homoserine lactone (AHL) signaling molecules. AHL is a QS signaling pathway from *Vibrio fischeri*. Functionally, toggle B2 enables an *E. coli* population to measure population density through AHL, because AHL signaling can be reversibly transported to the medium via diffusion, contributing to the AHL density in the culture [1]. To achieve an in-depth understanding of dynamic properties of coupled QS and toggle constructs, Kuznetsov et al. [2] developed and studied a mechanistic mathematical model of a population (or, equivalently, an ensemble) comprising N toggles, see Fig. 1 (bottom panel), corresponding to Toggle B2. Their study revealed important multiple functions, namely bistability as well as stable oscillations, that an ensemble of Toggles B2 was capable of exhibiting. Analytical conditions for bistability were found, and a time separation was introduced to obtain a stable limit cycle for a population of interacting cells.

In bistable circuits (toggles), transitions such as those caused by fluctuations due to low copy numbers of species per cell, or due to local environmental "noise" can force individual cells to change expression state at random [1]. This noise effect can spontaneously lead to the emergence of heterogeneous (mixed) populations consisting of cells in different expression states, which appear as bimodal population distributions when the corresponding protein levels are measured [1]. To investigate the effect of a spontaneous toggle switching in single and coupled cellular systems, leading to bimodal population distributions, Wang et al. [3] developed models for a single cell and a multi-cellular toggle system comprising N cells, respectively. In their models, the dynamics of the repressor proteins LacI and $\lambda\ CI$ is described by the two ODE equations developed in [4]. The AI-interfacing employed in the population model [3] corresponds to a signaling pathway which is slightly different from the signaling pathway in Toggle B2

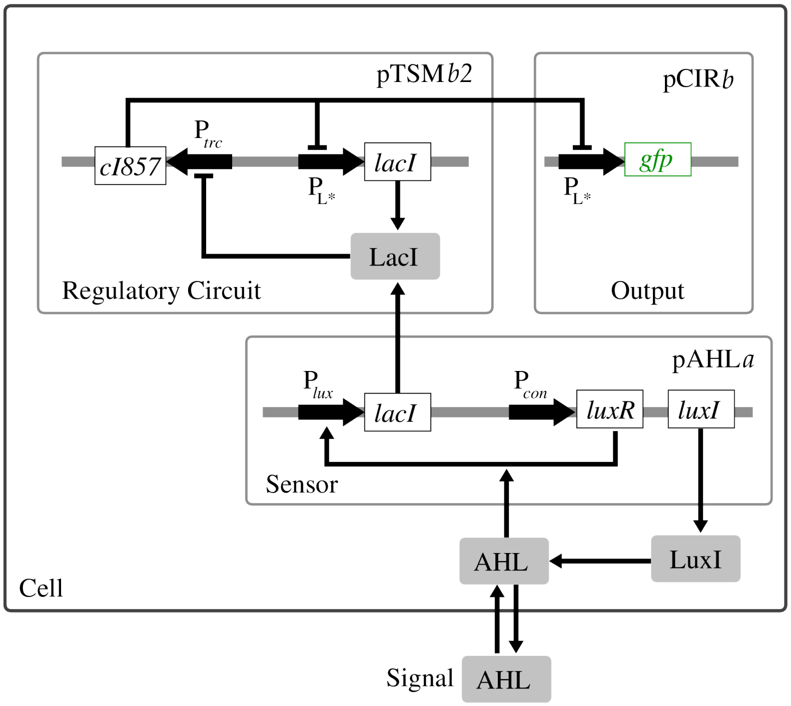

Figure SI-1.1. Toggle B2: Density-Dependent Gene Activation. *Notations and abbreviations.* Plasmids: pTSM*b2*, pCIR*b* and pAHL*a*; genes: *gfp*, *cI857*, *lacI*, *luxR*, and *luxI*; promoters: P_{trc}, P_{L^*}, and P_{lux}; AHL, acyl-homoserine lactone. Figure adapted from Fig. 6(A) in [1].

(Fig. SI-1.1) as suggested in [1] and described earlier. The main difference is in the description of the expression of the gene that encodes LuxI, see [3] for more details.

SI-2 Model Derivation

Here, we describe the main assumptions and steps used to derive mass-balance equations for the S- and A-models formulated in the main text.

SI-2.1 SI-2.1 Mass-Balance Equations

The derivation of the S- and A-models includes the development of two modules:

I. A transcription-translation module describing biosyntheis of repressor proteins.

II. A metabolic module describing biosynthesis of autoinducers.

A general and systematic discussion of both modules can be found in [5, 6]. The derivation of the first module for the A-model, relevant to our work, is given in [2]. Because one of our modeling objectives is to ultimately describe how the analysis of the mathematical models can be mechanistically interpreted in terms of tuning synthetic toggle "dials" by implementable experimental interventions as reviewed in [7], including modifications of ribosome-binding sites (RBS), carboxy-terminal tags, *etc.*, [4, 8, 9], we will derive mass-balance equations at the level of molecular detail sufficient to suggest plausible modeling predictions.

A transcription-translation module can be described by a basic two-stage model [2, 5, 6],

$$\frac{dn_x}{dt} = n_A k_x - r_x n_x, \tag{SI-2.1a}$$
$$\frac{dn_X}{dt} = k_X n_x - r_X n_X. \tag{SI-2.1b}$$

Here, n_x is the number of mRNA transcripts per cell for gene x, and n_X is the number of protein molecules per cell; n_A is the number of active promoters from which the mRNA of gene x is transcribed at an average rate k_x; k_X is the averaged translation rate; r_x and r_X are the effective first-order rate constants associated with degradation of the mRNA and proteins, respectively.

Since mRNA molecules are usually degraded rapidly compared to other cellular processes, a quasi-steady state for the equation (SI-2.1a) can often be assumed [2], yielding

$$n_x = \frac{n_A k_x}{r_x}. \tag{SI-2.2}$$

Using (SI-2.2) in the right-hand side of the equation (SI-2.1b), we obtain

$$\frac{dn_X}{dt} = k_X \frac{k_x\, n_A}{r_x} - r_X\, n_X. \tag{SI-2.3}$$

The ratio $b_x = k_X/r_x$ in equation (SI-2.3) is called a burst parameter of the protein X [2]. Using b_x in (SI-2.3) yields

$$\frac{dn_X}{dt} = b_x k_x n_A - r_X n_X. \tag{SI-2.4}$$

Assuming log-phase growth of *E. coli*, the volume $V(t)$ of the growing bacterium can be approximated by the expression $V(t) = V_0 \exp(\lambda t)$, and equation (SI-2.4) can be rewritten in a concentration form,

$$\frac{d[\mathrm{X}]}{dt} = b_x k_x [\mathrm{P_A}] - (r_X + \mu)\, [\mathrm{X}]. \tag{SI-2.5}$$

Here, $[\mathrm{X}](t) = n_X(t)/V(t)$ and $[\mathrm{P_A}](t) = n_A(t)/V(t)$.

The concentration of activate promoters, $[\mathrm{P_A}]$, can be computed, using an appropriate Hill function [6]. For example, we use

$$[\mathrm{P_{YA}}] = \frac{[\mathrm{P_Y}]}{1 + ([\mathrm{Y}]/K_\mathrm{Y})^{n_\mathrm{Y}}} \tag{SI-2.6}$$

for the repressor protein Y binding to the promoter $\mathrm{P_Y}$ with the dissociation constant $K_\mathrm{Y}^{n_\mathrm{Y}}$. In (SI-2.6), $[\mathrm{P_Y}]$ is the total concentration of all promoters $\mathrm{P_Y}$, while $[\mathrm{P_{YA}}]$ is the concentration of active promoters not bound with the repressor protein Y. Recall that the cooperativity described by the Hill exponent n_Y can arise from [4–6]:

(i). Multimerization of represssor proteins;

(ii). Cooperative binding of repressor multimers to multiple operator sites in the promoter.

Analogously, we use a Hill-function

$$[\mathrm{P_{GA}}] = [\mathrm{P_G}] \frac{([\mathrm{G}]/K_\mathrm{G})^{n_\mathrm{G}}}{1 + ([\mathrm{G}]/K_\mathrm{G})^{n_\mathrm{G}}} \tag{SI-2.7}$$

for the autoinducer G binding to the promoter $\mathrm{P_G}$ with the dissociation constant $K_{\mathrm{G}}^{n_{\mathrm{G}}}$ and the Hill exponent n_{G}. The concentration of all active promoters $\mathrm{P_A}$ can now be obtained from (SI-2.6) and (SI-2.7) as

$$\mathrm{P_A} = \mathrm{P_{YA}} + \mathrm{P_{GA}}. \tag{SI-2.8}$$

Using expression (SI-2.8), the equation (SI-2.5) can finally be updated as

$$\frac{d[\mathrm{X}]}{dt} = \frac{b_x k_x [\mathrm{P_Y}]}{1 + ([\mathrm{Y}]/K_{\mathrm{Y}})^{n_{\mathrm{Y}}}} + b_x k_x [\mathrm{P_G}] \frac{([\mathrm{G}]/K_{\mathrm{G}})^{n_{\mathrm{G}}}}{1 + ([\mathrm{G}]/K_{\mathrm{G}})^{n_{\mathrm{G}}}} - (r_{\mathrm{X}} + \mu)\,[\mathrm{X}]. \tag{SI-2.9}$$

Here, all parameters are described below in Table SI-3.2.

Similar mass balanced equations can be derived for the repressor protein Y, and synthases U and W. For example, the mass balance equation for the synthase U is

$$\frac{d[\mathrm{U}]}{dt} = \frac{b_u k_u [\mathrm{P_Y}]}{1 + ([\mathrm{Y}]/K_{\mathrm{Y}})^{n_{\mathrm{Y}}}} - (r_{\mathrm{U}} + \mu)\,[\mathrm{U}]. \tag{SI-2.10}$$

Analogously, we can write down a mass balance equation for the autoinducer concentration [G], that is, [C14-HSL], governed by the synthase U (CinI),

$$\frac{d[\mathrm{G}]}{dt} = k_{\mathrm{G}}\,[\mathrm{U}] + D_{\mathrm{G}}\,(\mathrm{G_e} - \mathrm{G}) - (r_{\mathrm{G}} + \mu)\,[\mathrm{G}]. \tag{SI-2.11}$$

Here, k_{G} is the maximum production rate of C14-HSL by CinI (Table SI-3.4), D_{G} is the export rate of C14-HSL (Table SI-3.4), and $[\mathrm{G_e}]$ is the extracellular concentration of C14-HSL.

Assuming that the concentration of the enzyme U reaches its quasi-steady state rapidly [10], one can obtain from (SI-2.10) that

$$[\mathrm{U}] = \frac{b_u k_u}{r_{\mathrm{U}} + \mu} \times \frac{[\mathrm{P_Y}]}{1 + ([\mathrm{Y}]/K_{\mathrm{Y}})^{n_{\mathrm{Y}}}}. \tag{SI-2.12}$$

Using (SI-2.12) in the equation (SI-2.11) yields

$$\frac{d[\mathrm{G}]}{dt} = \frac{b_u\, k_u\, k_{\mathrm{G}}}{r_{\mathrm{U}} + \mu} \times \frac{[\mathrm{P_Y}]}{1 + ([\mathrm{Y}]/K_{\mathrm{Y}})^{n_{\mathrm{Y}}}} - (r_{\mathrm{G}} + \mu)\,[\mathrm{G}]. \tag{SI-2.13}$$

Here, the definitions and the values of all parameters are given in Table SI-3.4. A similar mass balanced equation can be derived for the second autoinducer R (C4-HSL), and we omit the details.

SI-2.2 SI-2.2 Nondimensionalization

To nondimensionalize mass balance equations, as for example, the mass balance equations (SI-2.9) and (SI-2.13), we use the following dimensionless state variables, which are similar to those introduced in [2],

$$t' = (r_d + \mu)\,t, \quad x = \frac{[\mathrm{X}]}{K_{\mathrm{X}}}, \quad y = \frac{[\mathrm{Y}]}{K_{\mathrm{Y}}}, \quad g = \frac{[\mathrm{G}]}{K_{\mathrm{G}}}, \quad g_e = \frac{[\mathrm{G_e}]}{K_{\mathrm{G}}}, \quad r = \frac{[\mathrm{R}]}{K_{\mathrm{R}}}, \quad r_e = \frac{[\mathrm{R_e}]}{K_{\mathrm{R}}}. \tag{SI-2.14}$$

Here, we assume that all protein degradation rates can be set experimentally so that the following equalities can be obtained *approximately* [8],

$$r_{\mathrm{X}} = r_{\mathrm{X}} = r_{\mathrm{U}} = r_{\mathrm{W}} = r_d = \frac{\ln 2}{\tau_{1/2}}, \quad \tau_{1/2} = 4\,\mathrm{min}. \tag{SI-2.15}$$

The procedure of setting all protein degradation rates or, equivalently, all protein half-lives approximately equal to a prescribed value close to mRNA half-lives [8] is required to balance the toggle [4]. We discuss the balancing procedure and relevant experimental interventions in Sect. SI-2.2.

Using the dimensionless variables (SI-2.14), all original modeling mass balances can be nondimensionalized, yielding the S- and A-models formulated in the main text, where the prime is dropped from dimensionless time t'. In this case, dimensional and dimensionless parameters are related to one another as:

1. For the dimensionless rates, we obtain:

$$a_1 = \frac{b_x\, k_x\, [\mathrm{P_Y}]}{K_{\mathrm{X}}\,(r_d+\mu)}, \quad a_2 = \frac{b_y\, k_y\, [\mathrm{P_X}]}{K_{\mathrm{Y}}\,(r_d+\mu)}, \quad a_3 = \frac{b_x\, k_x\, [\mathrm{P_G}]}{K_{\mathrm{X}}\,(r_d+\mu)}, \tag{SI-2.16a}$$

$$a_4 = \frac{b_y\, k_y\, [\mathrm{P_R}]}{K_{\mathrm{Y}}\,(r_d+\mu)}, \quad a_5 = \frac{b_u\, k_u\, k_{\mathrm{G}}\, [\mathrm{P_Y}]}{K_{\mathrm{G}}\,(r_d+\mu)^2}, \quad a_6 = \frac{b_w\, k_w\, k_{\mathrm{R}} [\mathrm{P_X}]}{K_{\mathrm{R}}\,(r_d+\mu)^2}. \tag{SI-2.16b}$$

2. For dimensionless diffusion and degradation parameters, we obtain:

$$d_g = \frac{D_{\mathrm{G}}}{r_d+\mu}, \quad d_r = \frac{D_{\mathrm{R}}}{r_d+\mu}, \quad \delta_g = \frac{r_{\mathrm{G}}+\mu}{r_d+\mu}, \quad \delta_r = \frac{r_{\mathrm{R}}+\mu}{r_d+\mu}, \quad \delta_e = \frac{\mu_e}{r_d+\mu}, \tag{SI-2.17a}$$

Molecular and biophysical parameter values used in the expressions (5) - (6) will be estimated in Sect. SI-2.2, while dimensionless parameters will be estimated in Sect. SI-2.2 In this section, we only mention that due to [8], we can set

$$K_{\mathrm{X}} = K_{\mathrm{Y}} = 40\,\mathrm{monomers\,per\,cell}. \tag{SI-2.18}$$

We could not find any estimation of values for the two parameters K_{G} for C14-HSL and K_{R} for C4-HSL in the literature despite the fact that more and more precise measurements of kinetic parameters become available [11]. We estimate the order of magnitude of K_{G} and K_{R} as follows.

In the detailed experimental results on the C4-HSL-mediated quorum sensing regulatory system of the opportunistic Gram-negative bacterium *Aeromonas hydrophila*, the concentration of C4-HSL was found to be of order of magnitude equal to $10\,\mu$M [12]. In *E.coli* biology, it is convenient to use nM units [13], because relative to the effective *E. coli* volume [14], the value of 1 nM corresponds to one molecule per cell. This fact is widely used in the literature [8]. Therefore, the above estimate of $10\,\mu$M corresponds to 10^4 C4-HSL signaling molecules per cell.

Another *ad-hoc* rule of *E.coli* biology used in a number of studies with the Cornell *E.coli* computer model [15–18], resulting in a number of relevant predictions such as ribosomal-protein limitations, *lac*-control, plasmid stability, and *etc.* [14, 19–24], is that, the coarse-grained estimation for the dissociation equilibrium constant to be used in the Hill function can be calculated as 25% of the intracellular modifier (reference) concentration. In our case, this yields a coarse-grade estimate of 0.25×10^4 C4-HSL signaling molecules per cell,

$$K_{\mathrm{G}} = K_{\mathrm{R}} = 2.5 \times 10^3\,\mathrm{molecules\,per\,cell}. \tag{SI-2.19}$$

The values for other parameters will be estimated below.

SI-2.3 Toggle Balancing (Symmetrization)

As it was observed experimentally [4,8], synthetic circuits can operate and deliver the engineered traits only if special molecular constrains are fulfilled,

(i) repressor protein half-lives are close to mRNA halflifes [8];

(ii) repressor protein half-lives are approximately equal [4,8].

Constrain *(ii)* is required for "balancing" the given circuit [4]. Moreover, both works suggest experimental interventions to fulfill the above constraints [4,8]. Such and similar interventions are termed a "tuning dials" in the review [7].

It is mathematically convenient for us to generalize the above balancing procedure by the procedure of "symmetrization" of two antagonistic, mutually repressing toggle subsystems by selecting synthetic (tuned) parameter values that would make two antagonist subsystems symmetric to one another. In other words, we assume that an *ideal* S toggle has mirror symmetry corresponding to permutations between the two antagonistic subsystems. Symmetry usually helps with analytical analysis of nonlinear mathematical models.

Specifically, we "symmetrize" (balance) biosynthesis kinetic rates, using constraints

$$a_1 = a_2, \quad a_3 = a_4 \quad \text{and} \quad a_5 = a_6. \tag{SI-2.20}$$

Appropriate molecular interventions, which can be used to set the relationships (SI-2.20) approximately under certain experimental conditions, are reviewed in [7]. Similarly, we symmertize "diffusion" parameters,

$$d_g = d_r = d, \tag{SI-2.21}$$

and the autoinducer "degradation" or "utilization" ("load") parameters,

$$\delta_g = \delta_r = \delta_e = \delta. \tag{SI-2.22}$$

SI-3 Estimation of Parameter Values

SI-3.1 Toggle Parameters

In our estimations, we use general biochemical calculations [25, 26]. First, we collect generic prokaryotic and specific *E.coli* parameter values in Table SI-3.1.

Table SI-3.1. Generic prokaryotic and *E. coli* specific values of model parameters

Name	Description	Value	Unit	Reference
r_{mRNA}	Rate of transcription by RNA polymerase in prokaryotes	80	bp/sec	[27]
τ_{mRNA}	Typical half-life time for 80% of genes in *E. coli*	3 - 8	min	[28]
r_{aa}	Rate of translation by the ribosome in prokaryotes	20	aa/sec	[27][a]
k_{P}	Rate of translation by the ribosome in prokaryotes	1.71	$\sec^{-1}$	estimated in (SI-3.3)
T	*E. coli* replication period under specific nutrition conditions	25	min	[14]
μ	Intracellular specific dilution rate due to *E. coli* cell growth	$\ln 2/T$	$\min^{-1}$	[14]
μ_e	Extracellular dilution rate due to flow	0.1	$\min^{-1}$	[29]
ρ	Total volume fraction of cells in chamber	0.8	–	[30]
N	Number of *E. coli* cells in an overnight population culture	10^9 (OD600 = 1)	cells/ml	[14]

[a]This estimate is smaller than the estimate 33 aa/sec used in [11].

A general rate of translation of protein P in prokaryotes (k_{P})**.** Suppose that an mRNA transcript of protein P contains n_{aa} amino acids. Then, for one ribosome to transcribe P from its mRNA transcript, assuming a translation rate of 20 amino acids per second (Table SI-3.1), it will take time

$$t_{\mathrm{P}} = \frac{n_{\mathrm{aa}}}{20}. \tag{SI-3.1}$$

The above estimates yields the rate per ribosome which is

$$\frac{1 \text{ molecule}}{\mathrm{t_P}} = \frac{20}{\mathrm{n_{aa}}} \sec^{-1}. \tag{SI-3.2}$$

Given that the coding region of protein P is $n_{\mathrm{aa}} \times 3$ nucleotides long, and that a ribosome can attach every 35 nucleotides, we can estimate that $n_{\mathrm{aa}} \times 3/35$ ribosomes can be attached per mRNA molecule. We, thus, obtain

$$k_{\mathrm{P}} = \frac{20}{n_{\mathrm{aa}}} \times \frac{n_{\mathrm{aa}} \times 3}{35} = 1.71 \text{ protein molecules } \sec^{-1}. \tag{SI-3.3}$$

To illustrate our parameter estimation procedure, we derive parameter values for the Lac-repressor subsystem only. Parameter values for all other subsystems can be derived similarly.

k_x : **Fully induced strength of promoters** $\mathrm{P_Y}$ ($\mathrm{P_{tet}}$) **and** $\mathrm{P_G}$**.** One *lacI* mRNA transcript is 1204 bases long (Table SI-3.2). To transcribe one molecule of *lacI* mRNA from one gene with a rate of 80 bases per second (Table SI-3.2) takes

$$\frac{1204 \text{ bases}}{80 \text{ bases/sec}} = 15.05 \text{ sec}. \tag{SI-3.4}$$

Table SI-3.2. Parameter values of the LacI-repressor subsystem.

Name	Description	Value	Unit	Reference
Transcription:				
L_{lacI}	Size of *lacI* gene	1204	bp	[31]
Γ_x	Repressed strength of promoter P_{tet}	5.0×10^{-4}	molecules/cell/sec	[8]
k_x	Fully induced transcription rate[a]	6.65×10^{-2}	species/cell/sec	estimated in (SI-3.5)
K_Y	The number of TetR to repress[b] P_{tet}	40	monomers/cell	estimated in (SI-2.18)
K_G	The number of C14-HSL to activate[c] P_{cin}	2.5×10^3	molecules/cell	estimated in (SI-2.19)
$\tau_{1/2,lacI}$	Half-life of *lacI* mRNA	3.8	min	[32]
r_x	Rate of *lacI* mRNA degradation	3.04×10^{-3}	sec^{-1}	estimated in (SI-3.7)
n_Y	The number of subunits in TetR	2		[33]
n_G	Hill coefficient of C14-HSL	3		a reference value[d]
Translation:				
L_{LacI}	Size of one subunit in tetrameric LacI	360	aa/subunit	[34]
k_X	Rate of LacI translation	1.71	molecules/cell/sec	estimated in (SI-3.3)
$\tau_{1/2,LacI}$	Half-life of LacI protein	4	min	[8]
r_X	Rate of LacI degradation	1.16×10^{-3}	sec^{-1}	estimated in (SI-3.7)

[a]Fully induced strength of promoters P_{tet} and P_{cin},
[b]For the sake of brevity, "to repress" means "to half-maximally repress."
[c]For the sake of brevity, "to activate" means "to half-maximally activate."
[d] The Hill coefficients for C6-HSL and C12-HSL are estimated in the range of values 1 - 2 in [35,36], while these are estimated to be equal to 4 for C4-HSL and C14-HSL in [37]. We use a compromising *reference* value equal to 3 and also explore other values in our computational studies.

Then, per gene the estimate (SI-3.4) yields

$$k_x = \frac{1}{15.05 \text{ sec}} = 6.65 \times 10^{-2} \text{ lacI mRNA (molecules/cell/sec)}. \quad \text{(SI-3.5)}$$

The estimate (SI-3.4) is one order of magnitude less than the estimate 0.5 lacI mRNA (molecules/cell/sec) provided in [8].

r_x : **Rate of *lacI* mRNA degradation.** The calculation of degradation rates for proteins is based on the known protein half-lives,

$$r_x = \frac{\ln 2}{t_{1/2}}. \quad \text{(SI-3.6)}$$

We obtain (Table SI-3.2),

$$r_x = \frac{\ln 2}{3.8 \times 60 \text{ sec}} = 3.04 \times 10^{-3} \text{ sec}^{-1}. \quad \text{(SI-3.7)}$$

r_X : **Rate of LacI (X) degradation.** We obtain (Table SI-3.2),

$$r_d = r_X = \frac{\ln 2}{4 \times 60 \text{ sec}} = 2.89 \times 10^{-3} \text{ sec}^{-1}. \quad \text{(SI-3.8)}$$

We use the estimate (SI-3.8) for all proteins in the model.

Table SI-3.3. Parameter values of the TetA-repressor subsystem.

Name	Description	Value	Unit	Reference
Transcription:				
L_{tetR}	Size of *tetR* gene	905	bp	[38]
Γ_y	Repressed strength of promoter P_{lac}	5×10^{-4}	molecules/cell/sec	estimated in Table SI-3.2
k_y	Fully induced transcription rate[a]	8.87×10^{-2}	molecules/cell/sec	estimated
K_{X}	The number of LacI to repress[b] P_{lac}	40	monomers/cell	estimated in Table SI-3.2[d]
K_{R}	The number of C4-HSL to activate[c] P_{rhl}	5×10^{4}	monomers/cell	estimated in Table SI-3.2
$\tau_{1/2,tetR}$	Half-life of *tetR* mRNA	0.5	min	[39]
r_y	Rate of *tetR* mRNA degradation	2.31×10^{-2}	sec^{-1}	estimated
n_{X}	The number of subunits in LacI	2		[33]
n_{R}	Hill coefficient of C4-HSL	4		estimated in Table SI-3.2
Translation:				
L_{TetR}	Size of one subunit in tetrameric TetR	207	aa/subunit	[38]
k_{Y}	Rate of TetR translation	1.71	molecules/cell/sec	estimated in (SI-3.3)
$\tau_{1/2,\mathrm{TetR}}$	Half-life of TetR protein	4	min	estimated Table SI-3.2
r_{Y}	Rate of TetR degradation	1.16×10^{-3}	sec^{-1}	estimated

[a]Fully induced strength of promoters P_{lac} and P_{tet},
[b]For the sake of brevity, "to repress" means "to half-maximally repress."
[c]For the sake of brevity, "to activate" means "to half-maximally activate."
[d] Equilibrium dissociation constant for LacI is 7.7×10^{-8} M [40].

Table SI-3.4. Parameter values of the 3-OH-C14-HSL/CinI-signaling subsystem.

Name	Description	Value	Unit	Reference
Transcription:				
L_{cinI}	Size of *cinI* gene[a]	663	bp	estimated
Γ_u	Repressed strength of promoter P_{lac}	5×10^{-4}	molecules/cell/sec	estimated in Table SI-3.2
k_u	Fully induced strength of promoter P_{tet}	13.27×10^{-2}	molecules/cell/sec	estimated
$\tau_{1/2,cinI}$	Half-life of *cinI* mRNA	6.6	min	arbitrary[b]
r_u	Rate of *cinI* mRNA degradation	1.75×10^{-3}	sec^{-1}	estimated
Translation:				
L_{CinI}	Size of CinI aa-sequence	221	aa	http://string-db.org
k_{U}	Rate of CinI translation	1.71	molecules/cell/sec	estimated in Table SI-3.2
$\tau_{1/2,\mathrm{CinI}}$	Half-life[a] of CinI protein	4	min	[8]
r_{U}	Rate of protein CinI degradation	1.16×10^{-3}	sec^{-1}	estimated in Table SI-3.2
Signaling:				
k_{G}	Maximal production rate of CinI	2	min^{-1}	[41]
r_{G}	Degradation rate of C14-HSL	0.002	hr^{-1}	[42][c]
D_{G}	Export rate of C14-HSL	2.1	min^{-1}	[43]

[a]The coding region of the gene has been estimated from its protein sequence size provided in the same table as $221 \times 3 = 663$.
[b]This estimate corresponds to a general (or typical) pattern for mRNA half-lives in *E. coli* [44]. Note that [8] use a generic half-life parameter value of 2 min.
[c]Data for 3-OH-C12-HSL is used.

Table SI-3.5. Parameter values of the C4-HSL/RhlI-signaling subsystem.

Name	Description	Value	Unit	Reference
Transcription:				
L_{rhlI}	Length of *rhlI* gene	603	bp	[45]
Γ_w	Repressed strength of promoter P_{lac}	5×10^{-4}	molecules/cell/sec	estimated in Table SI-3.2
k_w	Fully induced strength of promoter P_{lac}	13.27×10^{-2}	molecules/cell/sec	estimated
$\tau_{1/2,rhII}$	Half-life of *rhlI* mRNA	6.6	min	[46][a]
r_w	Rate of *lacI* mRNA degradation	1.75×10^{-3}	sec^{-1}	estimated
Translation:				
L_{LacI}	Length of RhlI protein aa-sequence	196	aa	[47]
k_{W}	Rate of RhlI translation	1.71	molecules/cell/sec	estimated in Table SI-3.2
$\tau_{1/2,\text{RhlI}}$	Half-life of RhlI protein	4	min	estimated in Table SI-3.2
r_X	Rate of LacI degradation	1.16×10^{-3}	sec^{-1}	estimated
Signaling:				
k_{R}	Maximal production rate of C4-HSL by RhlI	16	min^{-1}	[41]
r_{R}	Degradation rate of C4-HSL	0.02	hr^{-1}	[42]
D_{R}	Export rate of C4-HSL	3.0	min^{-1}	[43]

[a]The half-life data for *lasI* mRNA is used because the degradation of *rhlI* is positively regulated by *LasI* [46] and, so, could have a longer half-life. This estimate is in line with a general (or typical) pattern for mRNA half-lives in *E. coli* [44]. Note that [8] use a genetic half-life parameter value of 2 min (Table SI-3.4).

SI-3.2 Dimensionless parameter values

Using data from Table SI-3.1 and the estimate (SI-3.8), we obtain

$$\mu = \frac{\ln 2}{25 \times 60} = 0.46 \times 10^{-3}\,\text{sec}^{-1},\ r_d + \mu = 3.35 \times 10^{-3}\,\text{sec}^{-1}. \tag{SI-3.9}$$

To estimate rates a_i, $i = 1 \dots 6$, defined in (5), we assume that the equalities $b_x = b_y = b_u = b_w = 10$ can be approximately set by using RBS-related interventions [7]. Also, to avoid competition for ribosomes, only a few plasmids bearing promoters P_X, P_Y, P_G, and P_R can be used. By selecting $[P_X] = [P_Y] = [P_G] = [P_R] = 1$ copies per cell, we obtain

$$a_1 = a_3 = \frac{10 \times \left(6.65 \times 10^{-2}\right) \times 1}{40 \times (3.35 \times 10^{-3})} = 4.96 \approx 5, \tag{SI-3.10a}$$

$$a_2 = a_4 = \frac{10 \times \left(8.87 \times 10^{-2}\right) \times 2}{40 \times (3.35 \times 10^{-3})} = 6.61 \approx 7, \tag{SI-3.10b}$$

$$a_5 = a_6 = \frac{10 \times \left(13.27 \times 10^{-2}\right) \times (2/60) \times 2}{(2.5 \times 10^3) \times (3.35 \times 10^{-3})^2} = 3.15 \approx 3. \tag{SI-3.10c}$$

Next, from (6), we obtain

$$d_g = \frac{2.1/60}{3.35 \times 10^{-3}} = 10.44, \tag{SI-3.11a}$$

$$d_r = \frac{3/60}{3.35 \times 10^{-3}} = 14.40, \tag{SI-3.11b}$$

$$\delta_g = \delta_r \approx \frac{\mu}{r_d + \mu} = \frac{0.46 \times 10^{-3}}{3.35 \times 10^{-3}} = 0.14, \tag{SI-3.11c}$$

$$\delta_e = \frac{0.1/60}{3.35 \times 10^{-3}} = 0.50. \tag{SI-3.11d}$$

We find the estimated values of the parameters to be of the same order of magnitude as the corresponding parameter values estimated and used in [2–4, 8, 35–37]. Not enough is yet known about molecular interactions inside host cells to obtain highly precise descriptions [7]; it is common to computationally evaluate the effect of different values for rate parameters and even for Hill exponents [2–4, 8]. Following [2, 4, 8], where genetic circuits built from similar elements have been studied, we have explored sets of parameter values which are close to the estimates given in (SI-3.10) and (SI-3.11), which ensure bistability in both S- and A-models, see the main text.

SI-4 Alternative Definitions of Monotone Systems and Order Preservation

We defined monotonicity using graph partitions because this is the easiest way to present the concept. However, the usual definition found in textbooks is not phrased in that form. We explain here how our definition is equivalent to the usual one as well as to another property. For further remarks on these equivalences, see [48]. A signed graph (such as the species influence graph obtained by looking at signs of Jacobain entries) G is said to be *balanced* (see Harary [49]) if every undirected closed loop in the graph G has a net positive sign, that is to say, an even number, possibly zero, of negative arrows. Equivalently, any two (undirected) paths between two nodes must have the same net sign. By undirected loops or paths, we mean that one is allowed to transverse an edge either forward or backward. A *spin assignment* Σ for the graph G is an assignment, to each node v_i, of a number σ_i equal to "+1" or "−1" (a "spin," to borrow from statistical mechanics terminology). If there is an edge from node v_j to node v_i, with label $J_{ij} \in \{\pm 1\}$, we say that this edge is *consistent with the spin assignment* Σ provided that:

$$J_{ij}\sigma_i\sigma_j = 1$$

which is the same as saying that $J_{ij} = \sigma_i\sigma_j$, or that $\sigma_i = J_{ij}\sigma_j$. An equivalent formalism is that in which edges are labeled by "0" or "1," instead of 1 and −1 respectively, and edge labels J_{ij} belong to the set $\{0, 1\}$, in which case consistency is the property that $J_{ij} \oplus \sigma_i \oplus \sigma_j = 0$ (sum modulo two). One says that Σ *is a consistent spin assignment for the graph* G (or simply that G is consistent) if every edge of G is consistent with Σ. In other words, for any pair of vertices v_i and v_j, if there is a positive edge from node v_j to node v_i, then v_j and v_i must have the same spin, and if there is a negative edge connecting v_j to v_i, then v_j and v_i must have opposite spins. (If there is no edge from v_j to v_i, this requirement imposes no restriction on their spins.) It is easy to see that if there is a consistent spin assignment for G, then the graph is balanced. Conversely, if G is balanced then there is a consistent spin assignment for G: to see this, simply label one node arbitrarily, and follow paths to label other nodes consistently. (If the graph is not connected, repeat the procedure in each connected component.)

For any spin assignment Σ, let A_1 be the subset of nodes labeled +1, and let A_{-1} be the subset of nodes labeled −1. The set of all nodes is partitioned into A_1 and A_{-1}. Conversely, any partition of the set of nodes into two subsets can be thought of as a spin assignment. With this interpretation, a consistent spin assignment is the same as a partition of the node set into two subsets A_1 and A_{-1} in such a manner that all edges between elements of A_1 are positive, all edges between elements of A_{-1} are positive, and all edges between a node in A_1 and a node in A_{-1} are negative. In summary, our definition of monotonicity, given in terms of partitions of state variables, amounts to the same as the requirement that there exist at least one consistent spin assignment for its associated graph G, or equivalently, that its graph G is balanced.

Supposing that a system is monotone, with a consistent spin assignment $\Sigma = \{\sigma_i, i = 1, \ldots, n\}$, we introduce following the relation among vectors $x \in \mathbb{R}^n_{\geq 0}$:

$$x \preceq y$$

means that

$$\sigma_i x_i \leq \sigma_i y_i \;\; i = 1, \ldots, n\,.$$

This is a componentwise inequality that requires $x_i \leq y_i$ if node i has been assigned a positive spin, and $x_i \geq y_i$ if instead node i has been assigned a negative spin. Let $y(t)$ and $z(t)$ be any two solutions of the system $dx/dt = f(x)$, and suppose that $\sigma_i y_i(0) \leq \sigma_i z_i(0)$ for each $i = 1, \ldots, n$. Then, *Kamke's Theorem* states that $\sigma_i y_i(t) \leq \sigma_i z_i(t)$ for all $t \geq 0$ and coordinate $i = 1, \ldots, n$. This is the usual definition of monotonicity: if states start at time zero in a certain order, then they must remain forever in the same order. Conversely, a flow that preserves an order of this type must be monotone in the sense that we have defined the concept. See the textbook [50] for a proof, and [51] for extensions with systems with external inputs.

The order preservation property has a variety of important implications for our model. For parameters viewed as constant states, it allows us to conclude the monotonicity of stable branches in bifurcation diagrams, as illustrated by the results described in Monotone Parametric Dependencies in the S design. A different implication concerns the domain of attraction of equilibria. Suppose that we consider an initial state $x(0)$ that is coordinate-wise less, in the monotone order, than a given equilibrium E (in a possibly multistable system). Comparing to E the solution $x(t)$ starting from this initial state $x(0)$, we know that $x(t)$ must remain less than E for all times. Thus, an equilibrium to which $x(t)$ converges must be upper bounded by E. In particular, if the equilibrium E is minimal (with respect to the coordinate-wise order), it follows that this trajectory converges to E. Similar conclusions apply to maximal equilibria E and initial states $x(0)$ that are coordinate-wise larger than E. One obtains in this manner a rich amount of information about the basin of attraction of equilibria in monotone systems.

SI-5 Symmetry

In this section, we formalize the symmetry of

- a single S toggle embedded into an environment ($N = 1$),
- a population of N-identical S toggles interacting via a common environment ($N \geq 2$).

SI-5.1 Symmetry of the S Model

As is routine in physics and engineering, symmetry-based simplifications often lead to important insights into complex phenomena [52], and we also use symmetry to discuss bifurcations in S toggle populations. We observe that under a special condition imposed on the parameter values,

$$a_1 = a_2,\ a_3 = a_4,\ a_5 = a_6,\ d_g = d_r,\ \delta_g = \delta_r, \tag{SI-5.1}$$

a single S toggle embedded into an external environment is described by the S model (1) with $N = 1$ which has a $\mathbb{Z}_2$-symmetry group generated by involution $\mathcal{I}$ [52–54],

$$\mathcal{I} : (x, y, g, r, g_e, r_e) \longrightarrow (y, x, r, g, r_e, g_e). \tag{SI-5.2}$$

Consider the fixed-point subspace $\mathrm{Fix}\,(\mathbb{Z}_2) \in \mathbb{R}^6$ of the group $\mathbb{Z}_2$, see [52],

$$\mathrm{Fix}\,(\mathbb{Z}_2) = \{z = (x, y, g, r, g_e, r_e) \in \mathbb{R}^6\,|\,\mathcal{I}z \equiv z\}. \tag{SI-5.3}$$

We ignore the trivial equilibria that belong to $\mathrm{Fix}\,(\mathbb{Z}_2)$, that is, equilibria of the S model (1) for which the following equalities hold, $x = y$, $g = r$, and $g_e = r_e$, corresponding to identically the same levels of LacI and TetR, and C14-HSL and C4-HSL, respectively. Let us denote the equilibrium of the S model (1) by z_0, and let us assume that $z_0 \notin \mathrm{Fix}\,(\mathbb{Z}_2)$. Now, because the S model is invariant with respect to the involution (SI-5.2), and because $z_0 \notin \mathrm{Fix}\,(\mathbb{Z}_2)$, we obtain that both z_0 and $\mathcal{I}\,z_0$, $\mathcal{I}\,z_0 \neq z_0$, are different equilibria of the S model (1), see [52, 53]. The equilibria z_0 and $\mathcal{I}\,z_0$ are called relative equilibria [55]. All bifurcations for the relative equilibria occur simultaneously at the same values of free parameters. We generalize G- and R-homogeneous populations states as relative equilibria, which means that as soon as the S toggle has a G-state, it will also have the corresponding R-state, implying bistability.

The general case of $N \geq 2$ is slightly more complicated as a population of identical S toggles has a symmetry group obtained after combinations of permutations among all cells in the given population and the toggle involution (SI-5.2), which we denote $\mathcal{G} = \mathbb{Z}_2 \times \mathbf{S}_N$ for brevity. Here, $\mathbf{S}_N$ is a symmetric group of order $N \geq 1$, and $\mathbb{Z}_2$ is the toggle involution (SI-5.2) applied to all toggles simultaneously. For example, for $N = 2$, we will have one permutation,

$$\mathcal{P} : (x_1, y_1, g_1, r_1, x_2, y_2, g_2, r_2, g_e, r_e) \longrightarrow (x_2, y_2, g_2, r_2, x_1, y_1, g_1, r_1, g_e, r_e), \tag{SI-5.4}$$

and the involution,

$$\mathcal{I} : (x_1, y_1, g_1, r_1, x_2, y_2, g_2, r_2, g_e, r_e) \longrightarrow (y_1, x_1, r_1, g_1, y_2, x_2, r_2, g_2, r_e, g_e). \tag{SI-5.5}$$

SI-5.2 Symmetry Breaking

The symmetry-breaking (pitchfork) bifurcation discussed around Fig. 7 in the main text has co-dimension one for all typical systems with $\mathbb{Z}_2$-symmetry [52,53]. To understand the symmetry-breaking occurring at the BP-point shown in Fig. 7, we need first to define the symmetry of the original symmetric (1:1)-mixed state. We observe that the original symmetric (1:1)-mixed state is invariant with respect to transformation g,

$$g = \mathcal{P} \circ \mathcal{I} = \mathcal{I} \circ \mathcal{P}, \quad g \circ g = \text{id} \tag{SI-5.6}$$

where id is the identity. Here, $\mathcal{P}$ and $\mathcal{I}$ are as defined in (SI-5.4) and (SI-5.5), respectively. The transformation (SI-5.6) forms subgroup $\Sigma_{(1:1)}$ of the group $\mathbb{Z}_2 \times \mathbf{S}_2$, see SI-5.1 Symmetry of the S Model, which consists of two elements, that is, $\Sigma_{(1:1)} = \{\text{id}, g\}$. The subgroup $\Sigma_{(1:1)}$ is called the *isotropy* subgroup [52] of the original (1:1)-mixed state. We further observe that the two (1:1)-states bifurcating from the original $\Sigma_{(1:1)}$-symmetric (1:1)-state at the BP-point (Fig. 7) are not invariant with respect to the isotropy subgroup $\Sigma_{(1:1)}$. Indeed, they are mapped one to one another by the transformation (SI-5.6). This observation motivates using the "symmetry-breaking" terminology [52] with respect to the loss of the isotropy subgroup symmetry by the (1:1)-mixed state at the BP-point. Due to the isotropy subgroup $\Sigma_{(1:1)}$ of the original (1:1)-mixed state, involution g defined in (SI-5.6) maps panel (A) to panel (D), and panel (B) to panel (C) within Fig. 7 of the main text. That is, $g : \text{LP}_1 \to \text{LP}_2$, $g : \text{LP}_2 \to \text{LP}_1$, and $g : \text{BP} \to \text{BP}$, see the coordinates of the three critical points, BP, LP_1, and LP_2, at the end of the caption of Fig. 7.

SI-5.3 A Remark on Bifurcations in Symmetric vs. Non-Symmetric Models

Mathematical models are idealizations of complex phenomena, based on certain assumptions, and there is a long established tradition to use symmetries in mathematical physics to clarify and explain complex phenomena. We use symmetry as another mathematical simplification alternative to simplifications arising from biological assumptions.

For our modeling studies with the S model, it may be difficult and even impossible to construct identical promoters which would correspond to identical values of parameters, that is, for example, $a_3 \neq a_4$. Therefore, it is required to discuss an appropriate interpretation of bifurcation diagrams computed for the S model.

First of all, we note that all LP-points will typically persist under small non-symmetric perturbations. However, all BP-points corresponding to pitchfork bifurcations will typically disappear under non-symmetric perturbations. They will typically be replaced by LP-bifurcation points. In such cases, in the small vicinity of the original BP point after a non-symmetric perturbation, we will typically have three branches of solutions, one branch of solutions which do not change their stability, and other two branches of solutions, stable and unstable, which will emanate from or collide with one another at the LP bifurcation point. Outside of the small vicinity of the perturbed BP-point, the bifurcation diagrams for both symmetric and non-symmetric models will be typically qualitatively the same. Such situations are mathematically very well studied and are described in the corresponding literature [52, 53].

SI-6 Exponential Stability of Cellular Populations

A systematic analysis of dynamical mathematical models begins with finding equilibrium solutions followed by the analysis of their exponential stability [56]. The next step is often to carry out (local) bifurcation analysis of the equilibrium solutions, allowing for the exploration of "stability boundaries" in the parameter space [53]. Both stability and bifurcation analyses rely on the computation of the eigenvalues from the corresponding model linearizations [53].

A nontrivial specificity of the computation of eigenvalues for the stability and bifurcation analyses of the A- and S-population models is that both models with $N > 1$ are invariant with respect to the action or the given linear representation of the *symmetric* group $\mathbf{S}_N$ of permutations among N-cells [52]. It is known that irreducible representations of groups enforce *multiple* eigenvalues of matrices that commute with their linear representations, a well-known fact following from Schur's Lemma in the representation theory of Lie groups [52].

To take into account the necessity to deal with multiple eigenvalues in the situations when the value of N is *a priori* unknown, we have developed a general approach to the analysis of exponential stability [56] in arbitrary populations of identical cells, independently of N, as described below.

A conceptually similar reduction approach (without any discussion of the multiplicity problem) on the exponential orbital stability of periodic solutions in systems of identical and slightly different oscillators coupled via a medium was developed by E. E. Shnol [57]. In his work, an averaging technique over the entire cellular population was used in both cases of homogeneous and mixed populations. Later, G. Katriel [58] has rediscovered the reduction result for homogeneous populations only, using Floquet Theory [59]. We note that the Schur's formula [60] can also be used to compute multipliers of periodic solutions in systems of coupled oscillators, using the linearizations of the corresponding Poincaré maps, in the very similar way as it is done for the case of equilibrium solutions in this work.

SI-6.1 A General Population Model of Identical Cells

In this SI, we use Schur's formula [60] to compute explicitly the characteristic polynomials for the corresponding model linearizations. The most important implication of Schur's formula is that it can be easily seen that the values of the eigenvalues are independent of $N \geq 2$.

To describe the general exponential stability analysis, we first introduce an appropriate notation as follows. Let S and z be "generalized" global (extracellular) and local (intracellular) state variables, respectively, $\dim S = m \geq 1$ and $\dim z = k \geq 2$. Using the generalized variables, both the S-model (1) and the A-model (2) can then be rewritten in the following general form, which we call a G-model,

$$\frac{dS}{dt} = H_0(S) + \frac{\rho}{N}\sum_{i=1}^{N} H(S, z_i), \qquad 0 \leq \rho \leq 1, \tag{SI-6.1a}$$

$$\frac{dz_i}{dt} = h(S, z_i), \quad i = 1, \dots N. \tag{SI-6.1b}$$

The G-model (SI-6.1) includes $m + Nk$ equations.

SI-6.2 A Homogeneous Population

In the case of a homogeneous population of identical cells, we have $z_i(t) \equiv z(t)$. As a result, the G-model (SI-6.1) reduces to a system of $(m+k)$-differential equations,

$$\frac{dS}{dt} = H_0(S) + \rho H(S,z), \qquad 0 \le \rho \le 1, \tag{SI-6.2a}$$

$$\frac{dz}{dt} = h(S,z). \tag{SI-6.2b}$$

Observe that the model (SI-6.2) describes a *single* cell placed in a "free", non-constant medium.

Definition 1. *Let* (S_0, z_0) *be an equilibrium solution of the model* (SI-6.2). *Then,* (S_0, z_0) *corresponds to a homogeneous population equilibrium solution,*

$$(S_0, z_0, \dots, z_0) = (S_0, N{\times}z_0)\,, \tag{SI-6.3}$$

of the full G-model (SI-6.1) *for any* $N \ge 2$*. Notation* $N{\times}z_0$ *means that* z_0 *is repeated* N*-times in* $(S_0, z_0, \dots, z_0)$*.*

Although the model (SI-6.2) is sufficient to study the *existence* of homogeneous population equilibrium solutions (SI-6.3), it is not enough to establish the exponential *stability* of the corresponding solutions (SI-6.3). Let (S_0, Nz_0) be a homogeneous population equilibrium solution of the G-model (SI-6.1) with any fixed $N \ge 2$. To analyze the exponential stability of $(S_0, N{\times}z_0)$ in the "full" G-model (SI-6.1), we need to compute the eigenvalues of the corresponding Jacobian matrix $\mathbf{J}_N$,

$$\mathbf{J}_N = \begin{pmatrix} \mathbf{A} & \frac{\rho}{N}\mathbf{B} & \frac{\rho}{N}\mathbf{B} & \dots & \frac{\rho}{N}\mathbf{B} \\ \mathbf{C} & \mathbf{D} & \mathbf{O} & \dots & \mathbf{O} \\ \mathbf{C} & \mathbf{O} & \mathbf{D} & \dots & \mathbf{O} \\ \vdots & \vdots & \vdots & \ddots & \vdots \\ \mathbf{C} & \mathbf{O} & \mathbf{O} & \dots & \mathbf{D} \end{pmatrix}. \tag{SI-6.4}$$

In (SI-6.4), each of three matrices, **B**, **C**, and **D**, is repeated N-times; **A** and **D** are square matrices of sizes m and k, respectively; **B** and **C** are rectangular matrices of sizes $m \times k$ and $k \times m$, respectively,

$$\mathbf{A} = \frac{\partial H_0}{\partial S} + \rho\frac{\partial H}{\partial S}, \quad \mathbf{B} = \frac{\partial H}{\partial z}, \quad \mathbf{C} = \frac{\partial h}{\partial S}, \quad \mathbf{D} = \frac{\partial h}{\partial z}. \tag{SI-6.5}$$

All partial derivatives in the expressions (SI-6.5) are evaluated at (S_0, z_0) which depends on all G-model parameters with the one important exception that they are *independent* of N because (S_0, z_0) is obtained using (SI-6.2). Notation **O** corresponds to zero submatrices of appropriate sizes.

We call a square matrix *stable* if all its eigenvalues have strictly negative real parts. The following theorem holds for J_N.

Theorem 1. (I). *Statements* (a), (b)*, and* (c) *are equivalent.*

(a). *The matrix* $\mathbf{J}_N$ *is stable for all* $N \ge 2$*.*

(b). *The matrix* $\mathbf{J}_1$ *and its submatrix* $\mathbf{D}$ *are both stable.*

(c). *The matrix* $\mathbf{J}_2$ *is stable.*

(II). *The matrix* $\mathbf{J}_N$ *has typically* k *different eigenvalues, each of multiplicity* $N-1$ *in the following sense. Let* $\{\lambda_1, \ldots, \lambda_{m+k}\}$ *be the set of eigenvalues of matrix* $\mathbf{J}_1$*, and let* $\{\mu_1, \ldots, \mu_k\}$ *be the set of eigenvalues of its submatrix* $\mathbf{D}$*, Then,*

$$\{\lambda_1, \ldots, \lambda_{m+k}, (N-1)(\mu_1, \ldots, \mu_k)\} \tag{SI-6.6}$$

is the set of all eigenvalues of matrix $\mathbf{J}_N$ *for any* $N \geq 2$*, where* $\{\mu_1, \ldots, \mu_k\}$ *is repeated* $(N-1)$*-times.*

Proof. Let λ be a complex number, $\lambda \in \mathbb{C}$. Consider a new matrix $\mathbf{M}_\lambda = \mathbf{J}_N - \lambda \mathbf{I}_{m+Nk}$, where $\mathbf{I}_{m+Nk}$ is the identity matrix of size $m + nk$. To find eigenvalues of $\mathbf{J}_N$, we need to write down the corresponding characteristic equation $P(\lambda) = 0$, $P(\lambda) = \det \mathbf{M}_\lambda$. Let us represent matrix $\mathbf{M}_\lambda$ in the form

$$\mathbf{M}_\lambda = \begin{pmatrix} \mathbf{A}_\lambda & \mathbb{B} \\ \mathbb{C} & \mathbb{D}_\lambda \end{pmatrix}. \tag{SI-6.7}$$

Here, matrices $\mathbf{A}_\lambda = \mathbf{A} - \lambda \mathbf{I}_m$, $\mathbb{B} = \frac{1}{N}(\mathbf{B}, \ldots, \mathbf{B})$, $\mathbb{C} = (\mathbf{C}, \ldots, \mathbf{C})^{\mathrm{T}}$, and $\mathbb{D}_\lambda = \operatorname{diag}(\mathbf{D}_\lambda, \ldots, \mathbf{D}_\lambda)$ with $\mathbf{D}_\lambda = \mathbf{D} - \lambda \mathbf{I}_k$. Next, assume for a moment that $\mathbb{D}_\lambda^{-1}$ exists. Then, Schur's formula can be used to compute $\det \mathbf{M}_\lambda$ [60],

$$\det \mathbf{M}_\lambda = \det \mathbb{D}_\lambda \cdot \det\left(\mathbf{A}_\lambda - \mathbb{B}\mathbb{D}_\lambda^{-1}\mathbb{C}\right). \tag{SI-6.8}$$

Next, we compute

$$\mathbb{B}\mathbb{D}_\lambda^{-1}\mathbb{C} = \mathbb{B}\left(\mathbb{D}_\lambda^{-1}\mathbb{C}\right) = \frac{1}{N}(\mathbf{B}, \ldots, \mathbf{B}) \begin{pmatrix} \mathbf{D}_\lambda^{-1}\mathbf{C} \\ \mathbf{D}_\lambda^{-1}\mathbf{C} \\ \cdots \\ \mathbf{D}_\lambda^{-1}\mathbf{C} \end{pmatrix} = \mathbf{B}\mathbf{D}_\lambda{}^{-\mathbf{1}}\mathbf{C}. \tag{SI-6.9}$$

For the determinant of the block diagonal $\mathbb{D}_\lambda$, we obtain $\det \mathbb{D}_\lambda = (\det \mathbf{D}_\lambda)^N$. Substituting (SI-6.9) into (SI-6.8) yields

$$\det \mathbf{M}_\lambda = (\det \mathbf{D})^n \cdot \det\left(\mathbf{A}_\lambda - \mathbf{B}\mathbf{D}_\lambda^{-1}\mathbf{C}\right). \tag{SI-6.10}$$

Using the Schur's formula for the product $\det \mathbf{D} \cdot \det\left(\mathbf{A}_\lambda - \mathbf{B}\mathbf{D}_\lambda^{-1}\mathbf{C}\right)$ in the "backward" direction, we can rewrite (SI-6.10) in the following equivalent form

$$\det \mathbf{M}_\lambda \ = \ (\det \mathbf{D}_\lambda)^{N-1} \cdot \det \begin{pmatrix} \mathbf{A}_\lambda & \mathbf{B} \\ \mathbf{C} & \mathbf{D}_\lambda \end{pmatrix}. \tag{SI-6.11}$$

The expression (SI-6.11) can now be rewritten simply as

$$P(\lambda) \ = \ (\det \mathbf{D} - \lambda \mathbf{I}_k)^{N-1} \cdot \det(\mathbf{J}_1 - \lambda \mathbf{I}_{m+k}). \tag{SI-6.12}$$

Recall that the expression (SI-6.12) has been proven under a restrictive condition $\det \mathbb{D}_\lambda \neq 0$, see above, which means that λ is not an eigenvalue of the matrix $\mathbf{D}$. This restriction can be

removed, for example, as follows. Let λ_0 be an eigenvalue of the matrix $\mathbf{D}$. Then, we obtain for the polynomial $P(\lambda)$ by continuity

$$P(\lambda_0) \;=\; \lim_{\lambda\to\lambda_0} P(\lambda) \;=\; \lim_{\lambda\to\lambda_0} (\det \mathbf{D} - \lambda \mathbf{I}_k)^{N-1} \cdot \det(\mathbf{J}_1 - \lambda \mathbf{I}_{m+k}) \;=\; 0. \qquad \text{(SI-6.13)}$$

It follows from (SI-6.13) that (SI-6.12) holds for all $\lambda \in \mathbb{C}$.

Finally, we observe from (SI-6.12) that to compute all eigenvalues of the Jacobian matrix $\mathbf{J}_N$ for any $N \geq 2$, it is sufficient to compute the eigenvalues of either two smaller matrices, $\mathbf{D}$ and $\mathbf{J}_1$, or one matrix $\mathbf{J}_2$. The latter may be practically slightly easier than computing the eigenvalues for $\mathbf{D}$ and $\mathbf{J}_1$ separately. The proof of the theorem follows. □

Consider a differential equation

$$\frac{dz}{dt} \;=\; h(S_0, z), \qquad \text{(SI-6.14)}$$

where S_0 is a fixed parameter corresponding to the equilibrium $(S_0, N \times z_0)$ of the full G-model (SI-6.1). In contrast to equation (SI-6.2), equation (SI-6.14) describes a single cell placed into a constant environment, which can be interpreted as an environment shaped by the large population of cells and which does not "sense" any changes in a single cell. Additionally, consider a cascade model

$$\frac{dS}{dt} \;=\; H_0(S) + \rho H(S, z_1), \quad 0 \leq \rho \leq 1, \qquad \text{(SI-6.15a)}$$

$$\frac{dz_j}{dt} \;=\; h(S, z_j), \quad j = 1, 2. \qquad \text{(SI-6.15b)}$$

Observe that the variable z_2 is absent from the first equation (SI-6.15a) and, hence, (SI-6.15) cannot be obtained from (SI-6.1) by simply setting $N = 2$.

Then, using the definition of exponential stability [56], the first statement of Theorem 1 can be reformulated as the following corollary which admits an intuitive interpretation of the fact why the case of $N = 2$ is sufficient to study the exponential stability of homogeneous population solutions.

Corollary 1. *Let* $(S_0, N \times z_0)$ *be an equilibrium solution of the G-model* (SI-6.1). *Then, statements* (a) - (*d*) *are equivalent.*

(a). $(S_0, N \times z_0)$ *is exponentially stable in the G-model* (SI-6.1) *for any* $N \geq 2$.

(b). (S_0, z_0) *is exponentially stable in the reduced model* (SI-6.2), *and* z_0 *is exponentially stable in the single-cell model* (SI-6.14).

(c). (S_0, z_0, z_0) *is exponentially stable in the G-model* (SI-6.1) *at* $N = 2$.

(*d*). (S_0, z_0, z_0) *is exponentially stable in the cascade model* (SI-6.15).

A comparison of Statements *(a)* and *(b)* of Corollary 1 leads to a conclusion that the given population consisting of identical cells is stable with respect to any small perturbation if and only if *(i)* the population is stable with respect to any small *uniform* perturbation of the entire population described by system (SI-6.1) and, simultaneously, *(ii)* a majority of *unperturbed* cells forces a single *slightly perturbed* cell to re-join back the unperturbed majority.

Indeed, system (SI-6.14) used in Statement *(b)* means that the entire population does not sense small perturbations in a single cell because S_0 is fixed in (SI-6.14).

Note that both conditions in Statement *(b)* can be reformulated, using the cascade model (SI-6.15) from statement (d). Finally, because the stability property is independent of the number N of identical cells in the population, the simple case of $N = 2$ can be used as given by statement *(c)*.

SI-6.3 A Mixed Population Split into Two Subpopulations

Suppose now that the given population consisting of N, $N \geq 4$, identical cells is split into two different subpopulations of sizes $N_1 \geq 2$ and $N_2 \geq 2$, respectively, where $N = N_1 + N_2$. We always assume that each subpopulation consists of at least two cells. Then, the two different homogeneous subpopulations can be described by two state variables z_1 and z_2, respectively, where $z_1 \neq z_2$, that is, $z_{i_p}(t) \equiv z_1(t)$ for some subset of indexes i_p, $p = 1, \dots, N_1$, and $z_{i_q}(t) \equiv z_2(t)$, for another subset of indexes i_q, $q = 1, \dots, N_2$. It follows that the equation (SI-6.1a) from the G-model (SI-6.1) simplifies as follows

$$\frac{dS}{dt} = H_0(S) + \frac{\rho}{N}\sum_{i=1}^{N} H(S, z_i) = \rho\Big(\beta_1 H(S, z_1) + \beta_2 H(S, z_2)\Big). \qquad \text{(SI-6.16)}$$

In (SI-6.16), β_j is the fraction of the j-th subpopulation, $\beta_j = N_j/N$, $j = 1, 2$, $\beta_1 + \beta_2 = 1$. In this case, the entire G-model (SI-6.1) reduces to the following three equations

$$\dot{S} = H_0(S) + \rho\Big(\beta_1 H(S, z_1) + \beta_2 H(S, z_2)\Big), \quad \beta_j \in \mathbb{Q}, \quad \beta_1 + \beta_2 = 1, \qquad \text{(SI-6.17a)}$$

$$\dot{z}_j = h(S, z_j), \quad j = 1, 2. \qquad \text{(SI-6.17b)}$$

Definition 2. *Let* (S_0, z_{10}, z_{20}), $z_{10} \neq z_{20}$, *be a non-uniform equilibrium solution of the reduced system* (SI-6.17). *Then,* (S_0, z_{10}, z_{20}), $z_{10} \neq z_{20}$ *corresponds to a mixed population equilibrium solution,*

$$(S_0, z_{10}, \dots, z_{10}, z_{20}, \dots, z_{20}) = (S_0, N_1 z_{10}, N_2 z_{20}), \qquad \text{(SI-6.18)}$$

of the full G-model (SI-6.1). *The solution* (SI-6.18) *describes a mixed population of* N *identical cells, split into two (non-identical) subpopulations of sizes* $N_1 > 0$ *and* $N_2 > 0$, *respectively,* $N_1 + N_2 = N$. *Notation* $N_j z_{j0}$ *means that* z_{j0} *is repeated* N_j*-times in* $(S_0, z_{10}, \dots, z_{10}, z_{20}, \dots, z_{20})$, $j = 1, 2$.

Due to the condition $\beta_1 + \beta_2 = 1$ used in (SI-6.17a), there formally exists a *continuum* of different fractions $\beta_1 : \beta_2$, $\beta_1 \in \mathbb{R}$ and $\beta_2 \in \mathbb{R}$. Of course, in the *biological* sense, only rational values $\beta_1 \in \mathbb{Q}$ and $\beta_2 \in \mathbb{Q}$ are allowable, leading to infinitely many fractional $(\beta_1 : \beta_2)$-configurations in the subdivision of the original population into two different subpopulations. Simple examples of such situations can be easily presented (Fig. SI-6.1).

For the sake simplicity of the exponential stability analysis, we will always assume that both β_1 and β_2 are real numbers, that is, $\beta_j \in \mathbb{R}$, $j = 1, 2$.

Let $(S_0, N_1 z_{10}, N_2 z_{20})$ be a mixed population equilibrium solution of the G-model (SI-6.1) with any fixed $N \geq 4$, see (SI-6.3). To analyze the exponential stability of $(S_0, N_1 z_{10}, N_2 z_{20})$, we

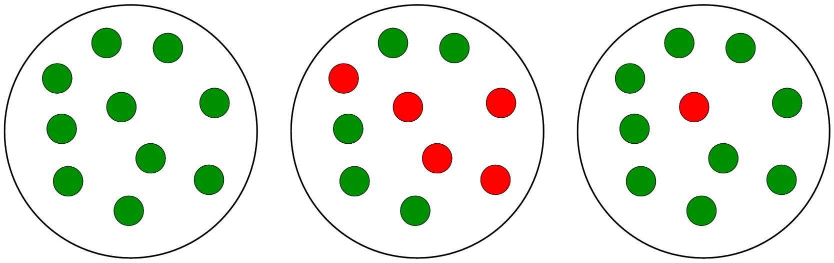

Figure SI-6.1. Examples of $(p:q)$-populations. The left panel corresponds to the case of a (10:0)-homogeneous population; the middle panel corresponds to the case of a (5:5)- or, equivalently, (1:1)-mixed population, and the right panel corresponds to a (9:1)-mixed population.

need to compute the eigenvalues of the corresponding Jacobian matrix $\mathbf{J}_N$ obtained from the G-model (SI-6.1),

$$\mathbf{J}_N = \begin{pmatrix} \mathbf{A} & \frac{\rho}{N}\mathbf{B}_1 & \dots & \frac{\rho}{N}\mathbf{B}_1 & \frac{\rho}{N}\mathbf{B}_2 & \dots & \frac{\rho}{N}\mathbf{B}_2 \\ \mathbf{C_1} & \mathbf{D}_1 & \dots & \mathbf{D}_1 & \mathbf{O} & \dots & \mathbf{O} \\ \vdots & \dots & \ddots & \dots & \vdots & \ddots & \vdots \\ \mathbf{C_1} & \mathbf{O} & \dots & \mathbf{D}_1 & \mathbf{O} & \dots & \mathbf{O} \\ \mathbf{C}_2 & \mathbf{O} & \dots & \mathbf{O} & \mathbf{D}_2 & \dots & \mathbf{O} \\ \vdots & \vdots & \vdots & \vdots & \vdots & \ddots & \vdots \\ \mathbf{C}_2 & \mathbf{O} & \dots & \mathbf{O} & \mathbf{O} & & \mathbf{D}_2 \end{pmatrix}. \tag{SI-6.19}$$

In the matrix (SI-6.19), submatrices $\mathbf{B}_i$, $\mathbf{C}_i$, and $\mathbf{D}_i$, are repeated N_j-times; $\mathbf{A}$ and $\mathbf{D}_i$ are square matrices of sizes m, and k, respectively; $\mathbf{B}_i$ and $\mathbf{C}_i$ are rectangular matrices of sizes $m \times k$ and $k \times m$, respectively, and

$$\mathbf{A} = \frac{\partial H_0}{\partial S} + \sum_{j=1}^{2} \rho_j \frac{\partial H(S_0, z_{j0})}{\partial S}, \tag{SI-6.20a}$$

$$\mathbf{B}_j = \frac{\partial H(S_0, z_{j0})}{\partial z}, \quad \mathbf{C}_j = \frac{\partial h(S_0, z_{j0})}{\partial S}, \quad \mathbf{D}_j = \frac{\partial h(S_0, z_{j0})}{\partial z}, \quad j = 1, 2. \tag{SI-6.20b}$$

Consider the Jacobian matrix $\mathbf{Q}_2$ of size $m + 2k$ for the system (SI-6.17), computed at (S_0, z_{10}, z_{20}),

$$\mathbf{Q}_2 = \begin{pmatrix} \mathbf{A} & \rho_1\mathbf{B}_1 & \rho_2\mathbf{B}_2 \\ \mathbf{C}_1 & \mathbf{D}_1 & \mathbf{O} \\ \mathbf{C}_2 & \mathbf{O} & \mathbf{D}_2 \end{pmatrix} \tag{SI-6.21}$$

Theorem 2. (I). *Statements* (a) *and* (b) *are equivalent.*

(a). *The matrix $\mathbf{J}_N$ is stable for all $N \geq 4$, and with any $N_1 \geq 2$ and $N_2 \geq 2$ such that $N_1 + N_2 = N$.*

(b). *Matrix $\mathbf{Q}_2$, and its two submatrices, $\mathbf{D}_1$ and $\mathbf{D}_2$, are stable.*

(II). *Matrix $\mathbf{J}_N$ has typically $2k$ different multiple eigenvalues in the following sense. Let $\{\lambda_1, \dots, \lambda_{m+2k}\}$ be the set of eigenvalues of $\mathbf{Q}_2$, let $\{\mu_1, \dots, \mu_k\}$ be the set of eigenvalues of $\mathbf{D}_1$, and let $\{\sigma_1, \dots, \sigma_k\}$ be the set of eigenvalues of $\mathbf{D}_2$. Then,*

$$\{\lambda_1, \dots, \lambda_{m+k},\ (N_1 - 1)\,(\mu_1, \dots, \mu_k)\,,\ (N_2 - 1)\,(\sigma_1, \dots, \sigma_k)\}\,, \tag{SI-6.22}$$

is the set of all eigenvalues of the matrix $\mathbf{J}_N$ *for any* $N \geq 4$. *In* (SI-6.22)*, the set* $\{\mu_1, \ldots, \mu_k\}$ *is repeated* $(N_1 - 1)$*-times, and the set* $\{\sigma_1, \ldots, \sigma_k\}$ *is repeated* $(N_2 - 1)$*-times. To have a nonzero value of multiplicity* $N_j - 1$ *in* (SI-6.22)*, condition* $N_j \geq 2$ *and, hence,* $N \geq 4$*, are natural requirements,* $j = 1, 2$*. In other words, the latter two conditions guarantee that both matrices* D_1 *and* D_2 *exist. Otherwise, Theorem 2 does not make any sense.*

Proof. The proof Theorem 2 can be carried out, using a simple modification of the proof of Theorem 1. For this reason, we only provide a brief sketch of the proof for Theorem 2. Similarly to the proof of Theorem 1, we need to write down a characteristic equation $P(\lambda) = 0$ Here $P(\lambda) = \det \mathbf{M}_\lambda$, and matrix $\mathbf{M}_\lambda$ can be defined as in (SI-6.7), using appropriate submatrices,

$$\mathbf{A}_\lambda = \mathbf{A} - \lambda \mathbf{I}_m, \tag{SI-6.23a}$$

$$\mathbb{B} = \frac{1}{N}\left(\mathbf{B}_1, \ldots, \mathbf{B}_1, \mathbf{B}_2, \ldots, \mathbf{B}_2\right), \tag{SI-6.23b}$$

$$\mathbb{C} = \left(\mathbf{C}_1, \ldots, \mathbf{C}_1, \mathbf{C}_2, \ldots, \mathbf{C}_2\right)^{\mathrm{T}}, \tag{SI-6.23c}$$

$$\mathbb{D}_\lambda = \operatorname{diag}\left(\mathbf{D}_{\lambda 1}, \ldots, \mathbf{D}_{1\lambda}, \mathbf{D}_{2\lambda}, \ldots, \mathbf{D}_{2\lambda}\right), \quad \mathbf{D}_{i\lambda} = \mathbf{D}_i - \lambda \mathbf{I}_k. \tag{SI-6.23d}$$

In the above submatrix definitions, the matrices with index j are repeated N_j-times, $i, j = 1, 2$.

In this case, $\det \mathbf{M}_\lambda$ can also be computed using Schur's formula, see (SI-6.8). However, (SI-6.9) should be replaced by

$$\mathbb{B}\mathbb{D}_\lambda^{-1}\mathbb{C} = \rho_1 \mathbf{B}_1 \mathbf{D}_{1\lambda}^{-1} \mathbf{C}_1 + \rho_2 \mathbf{B}_2 \mathbf{D}_{2\lambda}^{-1} \mathbf{C}_2. \tag{SI-6.24}$$

For the block diagonal matrix $\mathbb{D}_\lambda$, we obtain $\det \mathbb{D}_\lambda = \left(\det \mathbf{D}_{1\lambda}\right)^{N_1} \cdot \left(\det \mathbf{D}_{2\lambda}\right)^{N_2}$. Now, similarly to (SI-6.10), we will have

$$\det \mathbf{M}_\lambda = \left(\det \mathbf{D}_1\right)^{N_1} \cdot \left(\det \mathbf{D}_2\right)^{N_2} \cdot \det\Big(\mathbf{A}_\lambda - \rho_1 \mathbf{B}_1 \mathbf{D}_{1\lambda}^{-1} \mathbf{C}_1 - \rho_2 \mathbf{B}_2 \mathbf{D}_{2\lambda}^{-1} \mathbf{C}_2\Big). \tag{SI-6.25}$$

Using the Schur's formula in the "backward" direction, we will then have

$$P(\lambda) = \left(\det \mathbf{D}_1 - \lambda \mathbf{I}_k\right)^{N_1 - 1} \cdot \left(\det \mathbf{D}_2 - \lambda \mathbf{I}_k\right)^{N_2 - 1} \cdot \det \mathbf{Q}_2. \tag{SI-6.26}$$

The rest can be proved as in the proof for Theorem 1. The proof of Theorem 2 follows. □

Consider the following cascade model

$$\frac{dS}{dt} = H_0(S) + \rho\Big(\beta_1 H(S, z_1) + \beta_2 H(S, z_3)\Big), \quad \beta_1 + \beta_2 = 1, \tag{SI-6.27a}$$

$$\frac{dz_j}{dt} = h(S, z_j), \quad j = 1, \ldots, 4. \tag{SI-6.27b}$$

Variables z_2 and z_4 are absent from the first equation (SI-6.27a) and, hence, the cascade system (SI-6.27) cannot be obtained from the G-model (SI-6.1) by simply setting $N = 4$. Now, Theorem 2 can be reformulated in terms its Corollary 2 as follows.

Corollary 2. *Let* $(S_0, N_1 \times z_{10}, N_2 \times z_{20})$ *be a mixed population equilibrium solution of the G-model* (SI-6.1)*. Then, Statements* (a) - (c) *are equivalent.*

(a). $(S_0, N_1 \times z_{10}, N_2 \times z_{20})$ *is exponentially stable in the G-model* (SI-6.1) *for any* $N \geq 4$, *and with any* $N_1 \geq 2$ *and* $N_2 \geq 2$ *such that* $N_1 + N_2 = N$.

(b). (S_0, z_{10}, z_{20}) *is exponentially stable in the reduced model* (SI-6.17)*, and each* z_{j0} *is exponentially stable in the single-cell model* (SI-6.14)*,* $j = 1, 2$.

(c). $(S_0, z_{10}, z_{10}, z_{20}, z_{20})$ *is exponentially stable in the cascade model* (SI-6.27).

SI-6.4 A Mixed Population Split into Several Subpopulations

The case of a mixed population split into two subpopulations with densities $\rho_1 = \beta_1\rho$ and $\rho_2 = \beta_2\rho$ can be generalized to the case of a mixed population split into L-different subpopulations with densities $\rho_1, \dots, \rho_L$, where $\rho_1 + \dots + \rho_L = \rho$, $L \geq 3$ as follows.

Let subpopulation j consist of N_j cells, and let subpopulation j correspond to variable z_j, that is, we have $z_{j'}(t) \equiv z_j(t)$, where $j' \in \{i_1, i_2, \dots, i_{N_j}\} \subset \{1, 2, \dots N\}$, $j = 1, \dots, L$. In this case, the G-model (SI-6.1) reduces to the following equations

$$\dot{S} = H_0(S) \; + \; \sum_{j=1}^{L} \rho_j H(S, z_j), \quad \sum_{j=1}^{L} \rho_j = \rho, \tag{SI-6.28a}$$

$$\dot{z}_j = h(S, z_j), \quad \rho_j = \beta_j \rho, \quad \beta_j = \frac{N_j}{N}, \quad j = 1, \dots, L. \tag{SI-6.28b}$$

Definition 3. *Let* $(S_0, z_{10}, \dots, z_{L0})$ *be a non-uniform equilibrium solution of the system* (SI-6.28), *where* $z_{j0} \neq z_{j'0}$ *for all* $j \neq j'$. *Then,* $(S_0, z_{10}, \dots, z_{L0})$, *corresponds to a mixed population equilibrium solution,*

$$(S_0, N_1 z_{10}, \dots, N_L z_{L0}), \tag{SI-6.29}$$

of the full G-model (SI-6.1). *The solution* (SI-6.29) *describes a mixed population of* N *identical cells, which is split into* L *subpopulations of the corresponding sizes* $N_j \geq 2$, $N_1 + \dots + N_L = N$. *Notation* $N_j z_{j0}$ *means that* z_{j0} *is repeated* N_j*-times in the vector-form solution of the the full G-model* (SI-6.1), $N_j \geq 2$, $j = 1, \dots, L$.

Consider the Jacobian matrix $\mathbf{Q}_L$ for the reduced system (SI-6.28), computed at $(S_0, z_{10}, \dots, z_{L0})$,

$$\mathbf{Q}_L = \begin{pmatrix} \mathbf{A} & \rho_1 \mathbf{B}_1 & \dots & \rho_L \mathbf{B}_L \\ \mathbf{C}_1 & \mathbf{D}_1 & \dots & \mathbf{O} \\ \vdots & \vdots & \ddots & \vdots \\ \mathbf{C}_L & \mathbf{O} & \dots & \mathbf{D}_L \end{pmatrix}. \tag{SI-6.30}$$

In (SI-6.30), all submatrices are defined as in (SI-6.20), where $j = 1, 2$ should be replaced by $j = 1, \dots, L$. Below, we formulate Theorem 3 and Corollary 3 without any proof because they are similar to Theorem 2 and Corollary 1, respectively.

Theorem 3. (I). *Statements* (a) *and* (b) *are equivalent.*

(a). *The Jacobian* $\mathbf{J}_N$ *computed for the G-model at the given equilibrium* (SI-6.29) *is stable for all* $N \geq 2L$, *and with any* $N_j \geq 2$, $j = 1, \dots, L$, *such that* $N_1 + \dots N_L = N$.

(b). *Matrix* $\mathbf{Q}_L$ *and its submatrices* $\mathbf{D}_j$, $j = 1, \dots, L$, *are stable.*

(II). *Matrix* $\mathbf{J}_N$ *has typically* kL *different multiple eigenvalues in the following sense. Let* $\{\lambda_1, \dots, \lambda_{m+kL}\}$ *be the set of eigenvalues of* $\mathbf{Q}_L$, *and let* $\{\mu_1^{(j)}, \dots, \mu_k^{(j)}\}$ *be the set of eigenvalues of* $\mathbf{D}_j$, $j = 1, \dots, L$. *Then,*

$$\left\{\lambda_1, \dots, \lambda_{m+k}, \; (N_1 - 1)\left(\mu_1^{(1)}, \dots, \mu_k^{(1)}\right), \dots, (N_L - 1)\left(\mu_1^{(N_L)}, \dots, \mu_k^{(N_L)}\right)\right\} \tag{SI-6.31}$$

is the set of all eigenvalues of the matrix $\mathbf{J}_N$ *for any* $N \geq 2L$. *In* (SI-6.31), *each set* $\{\mu_1^{(j)}, \dots, \mu_k^{(j)}\}$ *is repeated* $(N_j - 1)$*-times with all* $N_j \geq 2$, $j = 1, \dots, K$.

Consider the following cascade model

$$\frac{dS}{dt} = H_0(S) + \sum_{j=1}^{L} \rho_j H(S, z_{2j-1}), \quad \sum_{j=1}^{L} \rho_j = \rho, \tag{SI-6.32a}$$

$$\frac{dz_j}{dt} = h(S, z_j), \quad j = 1, \ldots, 2L. \tag{SI-6.32b}$$

State variables z_{2j} with even indeces are absent from the first equation (SI-6.32a) of the cascade model (SI-6.32).

Corollary 3. *Let* $(S_0, N_1 z_{10}, \ldots, N_L z_{L0})$ *be a mixed equilibrium solution of the G-model* (SI-6.1)*, where* $N_{j0} \neq N_{j'0}$ *for all* $j \neq j'$*. Then, Statements* (a) - (c) *are equivalent.*

(a). $(S_0, N_1 z_{10}, \ldots, N_L z_{L0})$ *is exponentially stable in the G-model* (SI-6.1) *for any* $N \geq 2L$*, and with any* $N_j \geq 2$*,* $j = 1, \ldots, L$*, such that* $N_1 + \ldots N_L = N$*.*

(b). $(S_0, z_{10}, \ldots, z_{L0})$ *is exponentially stable in the reduced model* (SI-6.28)*, and each* z_{j0} *is exponentially stable in the single-cell model* (SI-6.14)*,* $j = 1, \ldots, L$*.*

(c). $(S_0, z_{10}, z_{10}, z_{20}, z_{20}, \ldots, z_{L0}, z_{L0})$ *is exponentially stable in the cascade model* (SI-6.32)*.*

SI-7 Additional Figures

Figure SI-7.1

In accordance with predictions from Fig. 3, we observe that an increase in the values of δ_g lead to suppressed levels in x_1 (LacI) as well as to elevated levels in y_1 (TetR). This is illustrated in Fig. SI-7.1. The almost constant dependencies in Fig. SI-7.1 (C) and (D) can be explained by suppressed levels of TetR and C4-HSL in the G-population.

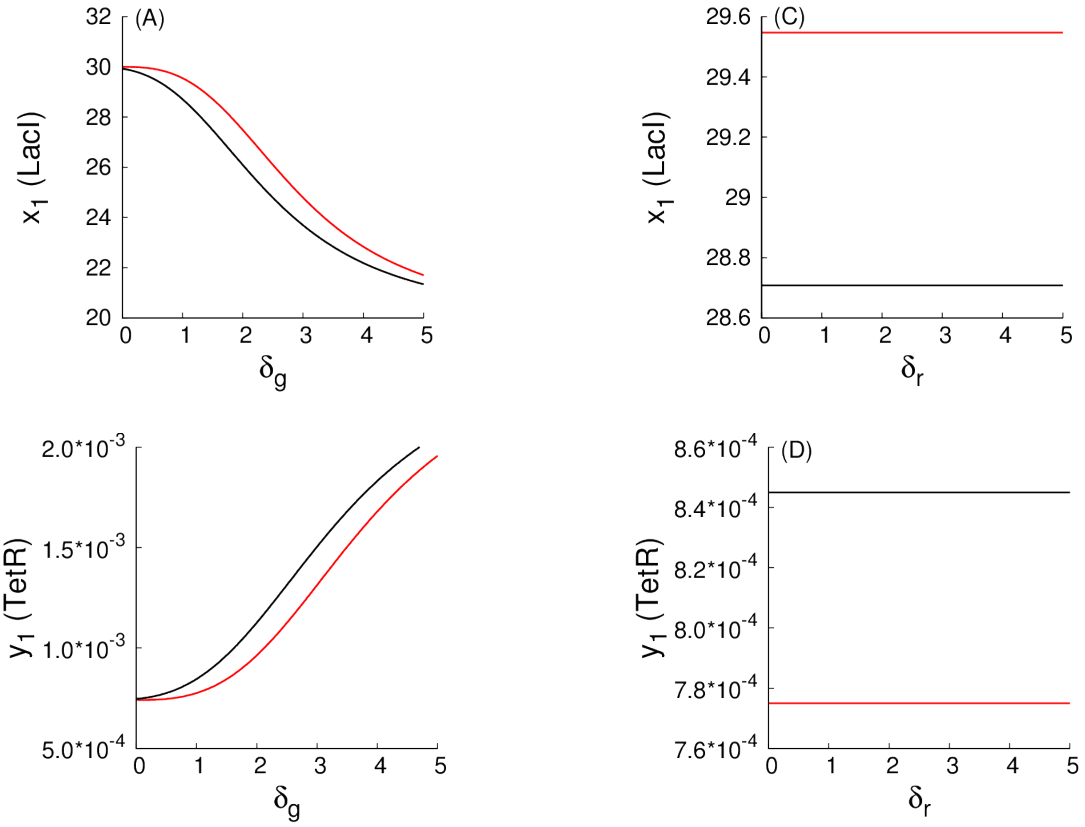

Figure SI-7.1. Examples of monotone parametric dependencies for the repressor-protein levels in the G-homogeneous state. Red solid curves correspond to a weak coupling among all toggles ($d = 0.1$), while black solid curves correspond to a strong coupling among all toggles ($d = 10$).

Analogously (Fig. SI-7.1), an increase in the values of δ_g should also lead to suppressed levels in g_1 (C14-HSL) as well as to elevated levels in r_1 (C4-HSL), while an increase in the values of δ_r should lead to elevated levels in g_1 (C14-HSL) and, simultaneously, to decreased levels in r_1 (C4-HSL). This is illustrated in Fig. SI-7.2. Constant dependencies in Fig. SI-7.2 (C) can be explained by suppressed levels of TetR and C4-HSL in the G-population.

Figure SI-7.2

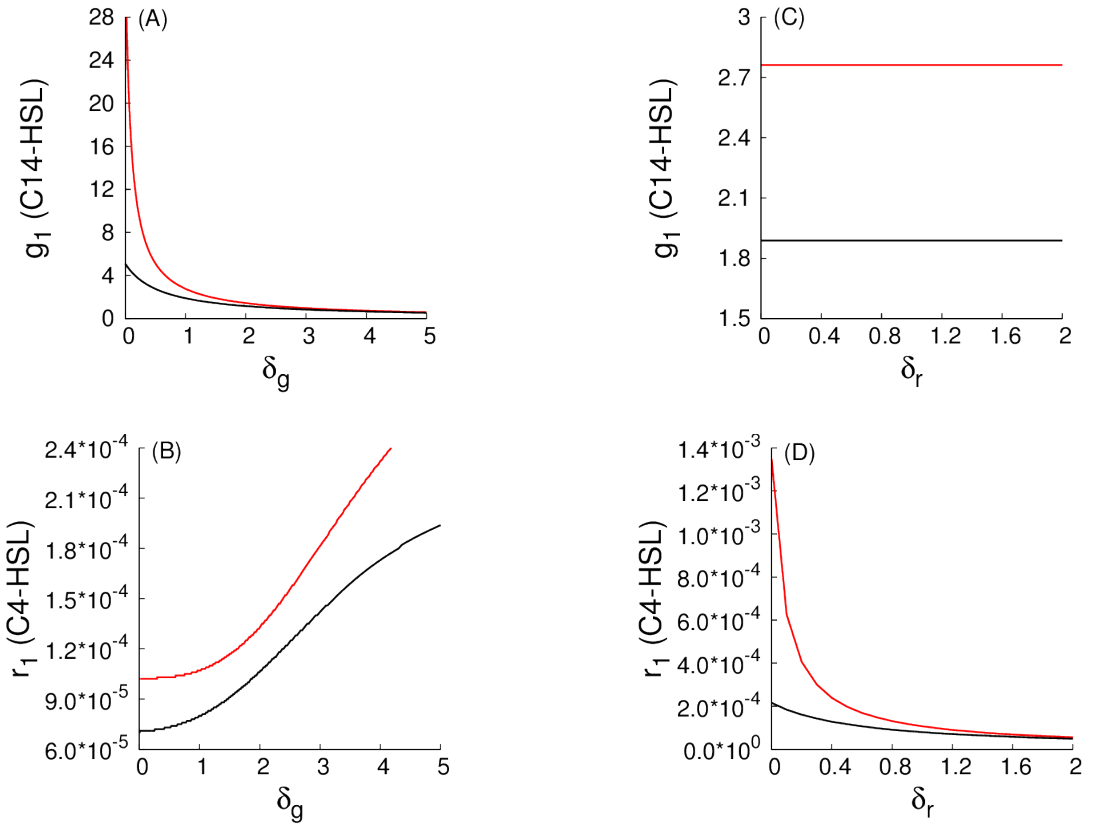

Figure SI-7.2. Examples of monotone parametric dependencies for the signaling species levels in the G-homogeneous state. Red solid curves correspond to a weak coupling among all toggles ($d = 0.1$), while black solid curves correspond to a strong coupling among all toggles ($d = 10$).

Figure SI-7.3

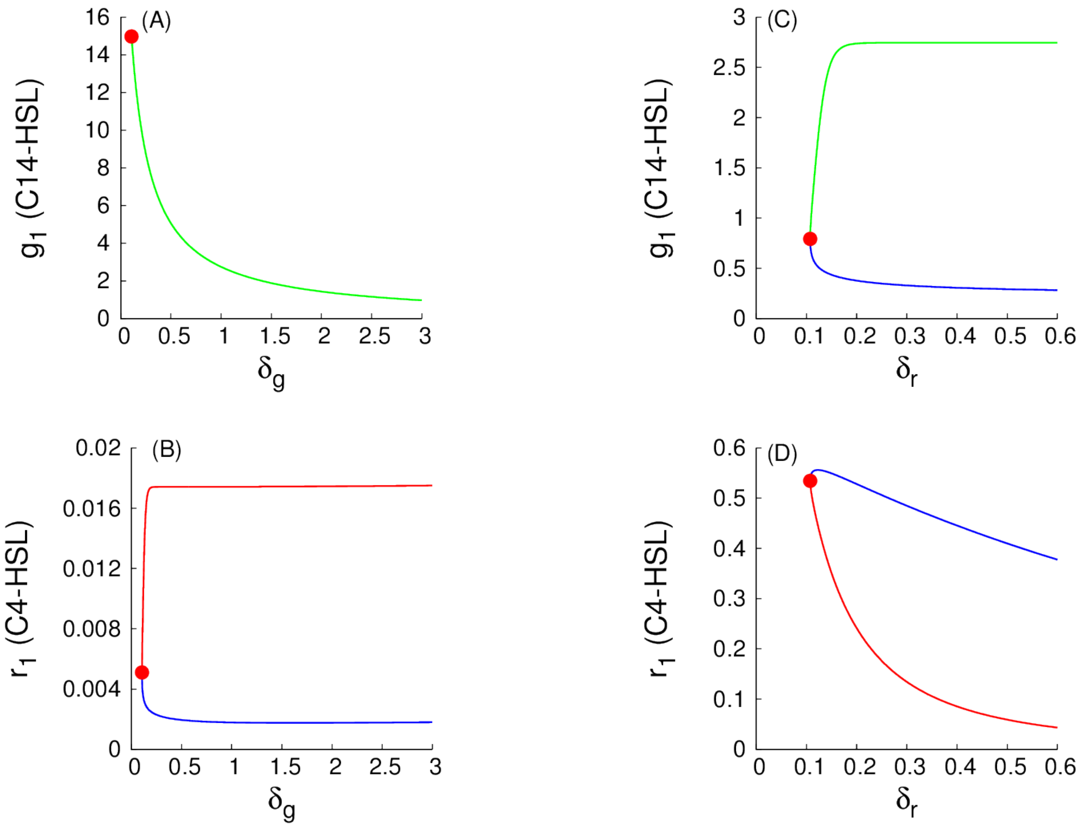

Figure SI-7.3. Examples of monotone parametric dependencies for the signaling species levels in the (1:1)-mixed state. All explanations are as in Fig. 5.

Figure SI-7.4

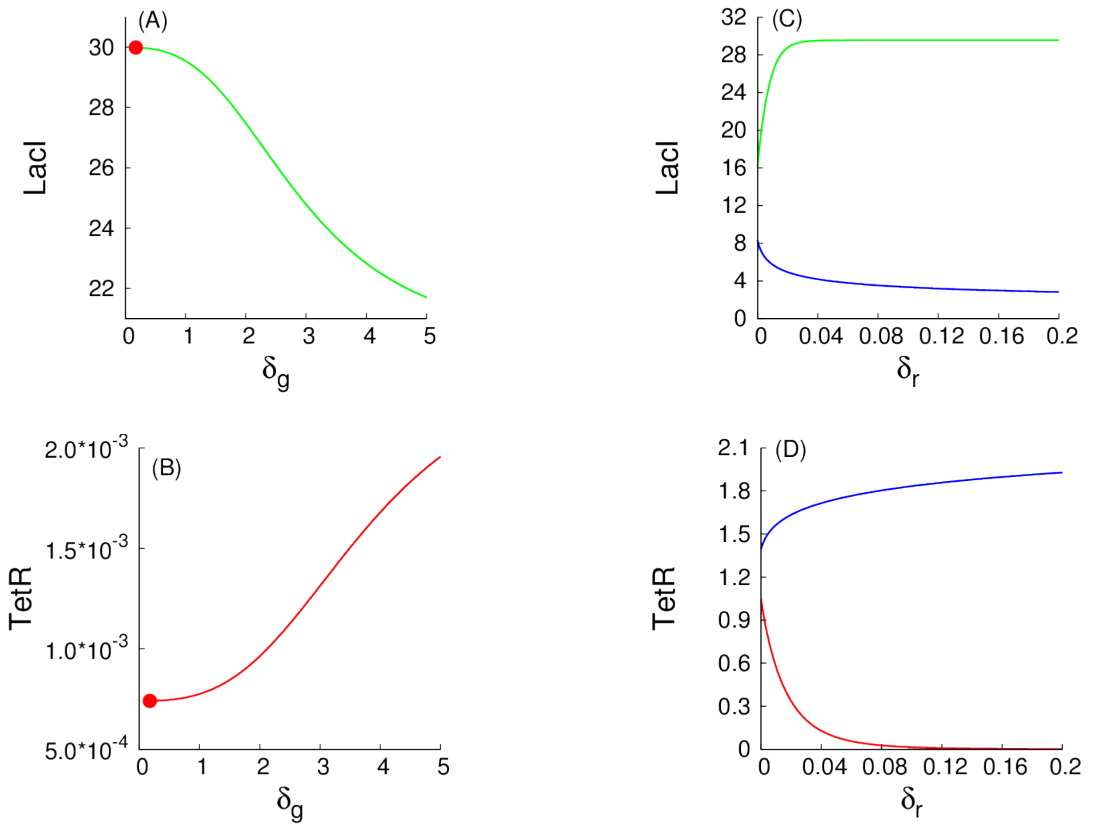

Figure SI-7.4. Examples of monotone parametric dependencies for the repressor-protein levels in the (9:1)-mixed state (a 90% large G-subpopulation.) Green and red solid curves correspond to stable solution branches, while all blue curves correspond to unstable solution branches. Red filled circles correspond to an LP-bifurcation point. In panels (A) and (B), projections of stable and unstable solution branches coincide and, so, only the stable solution branches are shown.

The monotone parametric dependencies for a (9:1)-mixed state corresponding to a spontaneous synchronization error are illustrated in Fig. SI-7.4 for a large G-subpopulation comprising 90% of all cells, and in Fig. SI-7.5 for a small R-subpopulation comprising 10% of all cells in the given (9:1)-mixed state.

We observe that LP-bifurcation points are present in both panels (A) and (B), and are absent from both panels (C) and (D) in Fig. SI-7.4 and Fig. SI-7.5. To explain this observation we have to recall the difference between parameters δ_g and δ_r. As discussed earlier, a decrease in the values of δ_g can be interpreted in terms of the improved communication between the toggles within the large subpopulation, while a decrease in the values of δ_r can be interpreted in terms of the improved communication between the toggles within the small subpopulation.

Figure SI-7.5

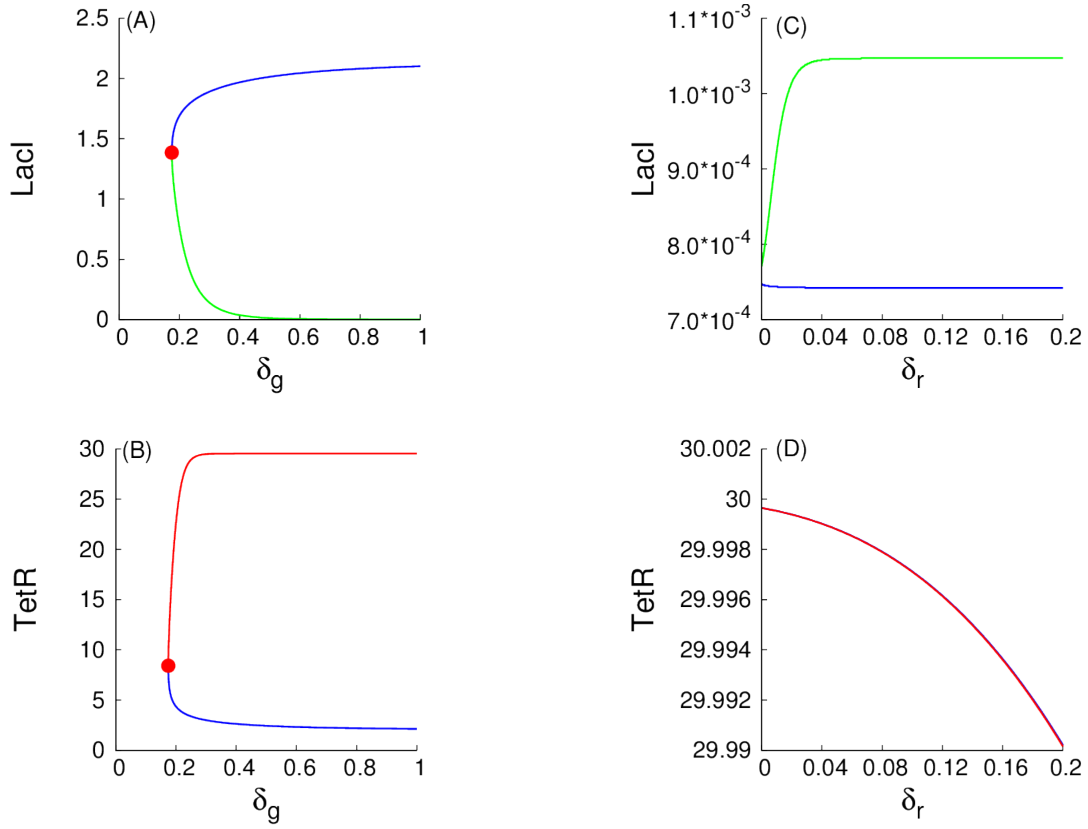

Figure SI-7.5. Examples of monotone parametric dependencies for the repressor-protein levels in the (9:1)-mixed state (a 10% small R-subpopulation.) Green and red solid curves correspond to stable solution branches, while all blue curves correspond to unstable solution branches. In panel (D), projections of stable and unstable solution branches coincide. Red filled circles in panels (A) and (B) correspond to an LP-bifurcation point.

Figure SI-7.6

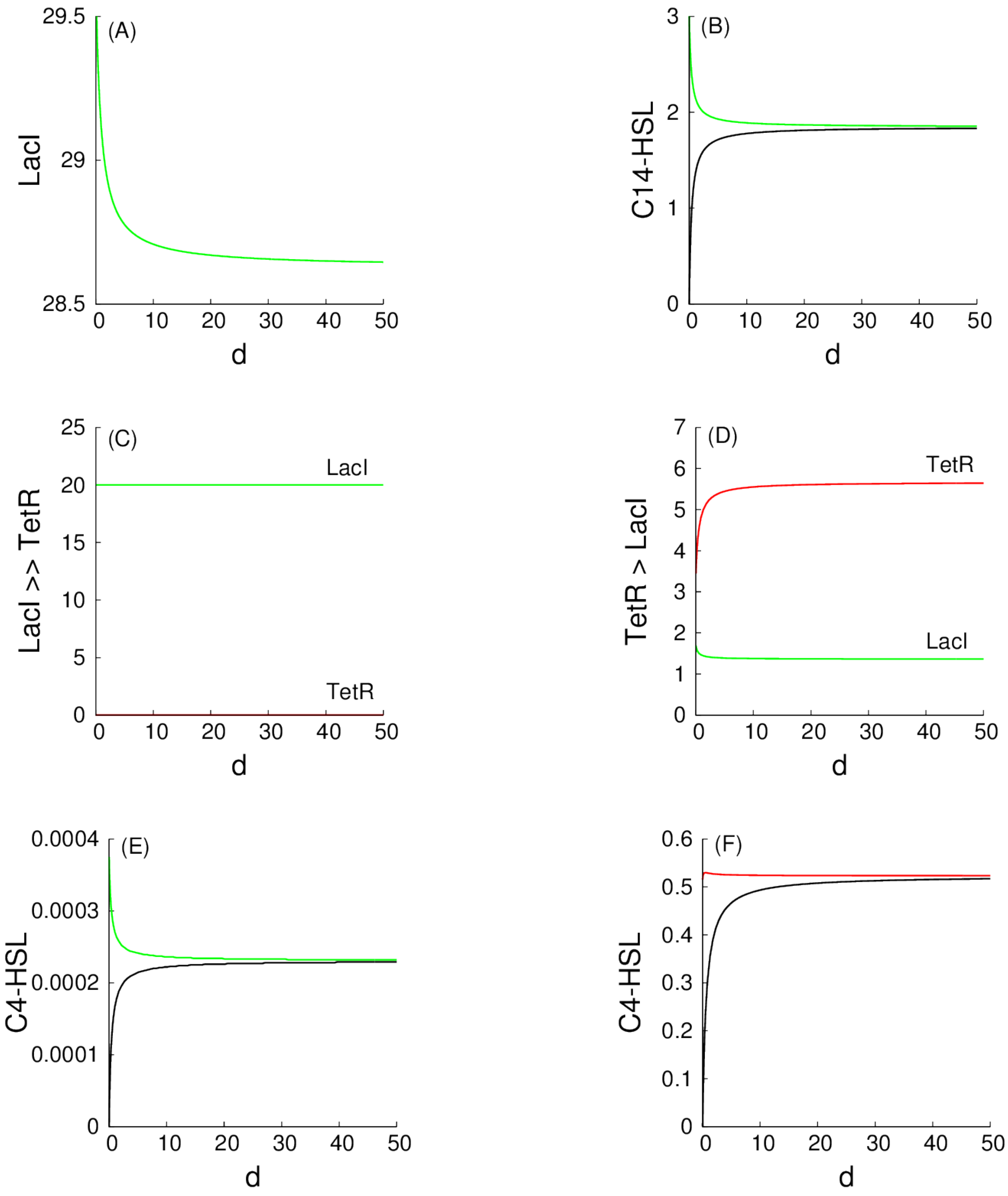

Figure SI-7.6. Homogeneous populations of S and A toggles. Dependencies of G-homogeneous populations on the values of the parameter d are shown. Top panels (A) and (B) correspond to a G-homogeneous population of S toggles. Panel (A) presents (dimensionless) levels of the activated LacI, while levels of the repressed TetR are of order of magnitude about 10^{-3} and are not shown. Panel (B) presents levels of C14-HSL. The green curve corresponds to the intracellular levels, while the black plot corresponds to extracellular levels of C14-HSL, respectively. Panels (C) and (E) present levels of the activated LacI and C4-HSL obtained for the A toggle settled at the G-state (LacI > TetR). Panels (D) and (F) present levels of the activated TetR and C4-HSL obtained for the A toggle settled at the R-state (TetR > LacI).

We observe from Fig. SI-7.6 that the intracellular and extracellular levels of the QS signaling molecule C14-HSL become asymptotically indistinguishable from one another as $d \to \infty$. The asymptotic behavior of the S toggle for large values of d can be analytically understood after introducing a small parameter $\varepsilon = d^{-1}$ into the S-model (1) which becomes a singularly-perturbed problem [61]. Setting formally $\varepsilon = 0$ in the singularly-perturbed problem as required by the theory of singular perturbations [61], the differential equations (SI-8.1c) and (SI-8.1d) can be reduced to elementary algebraic equations $g = g_e$ and $r = r_e$, respectively.

Figure SI-7.7

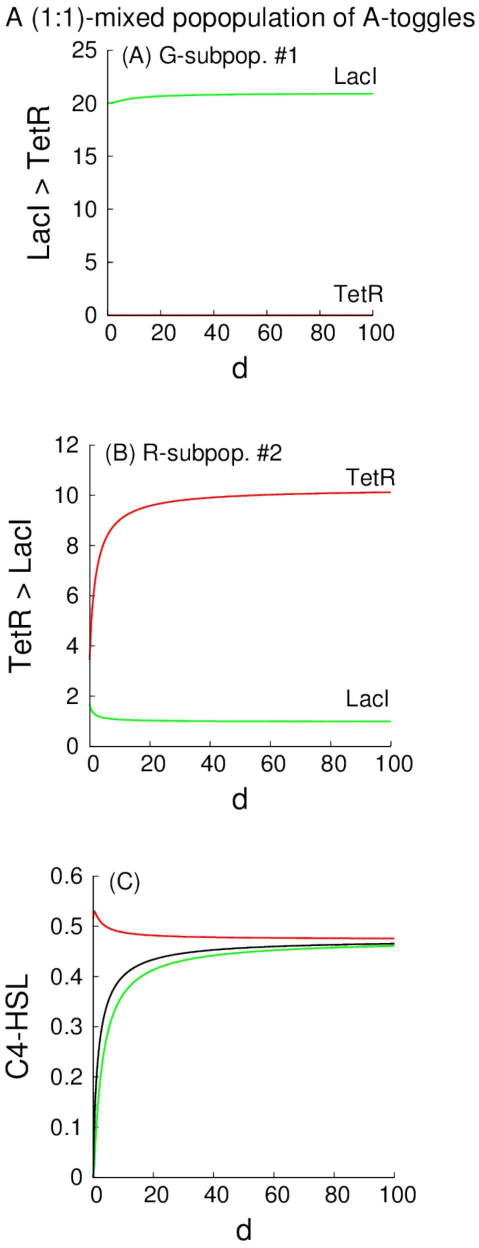

Figure SI-7.7. A (1:1)-mixed population of A toggles. Green and red color coded curves in panels (A) and (B) correspond to the intracellular concentrations of LacI and TetR, respectively, while a black color-coded curve in panel (C) corresponds to the extracellular concentration of C4-HSL. In panel (C), the green color-coded curve corresponds to the concentration of C4-HSL within the G-subpopulation, that is, LacI > TetR as in panel (A), while the red color-coded curve corresponds to the R-subpopulation, that is, TetR > LacI as in panel (B).

Figure SI-7.8

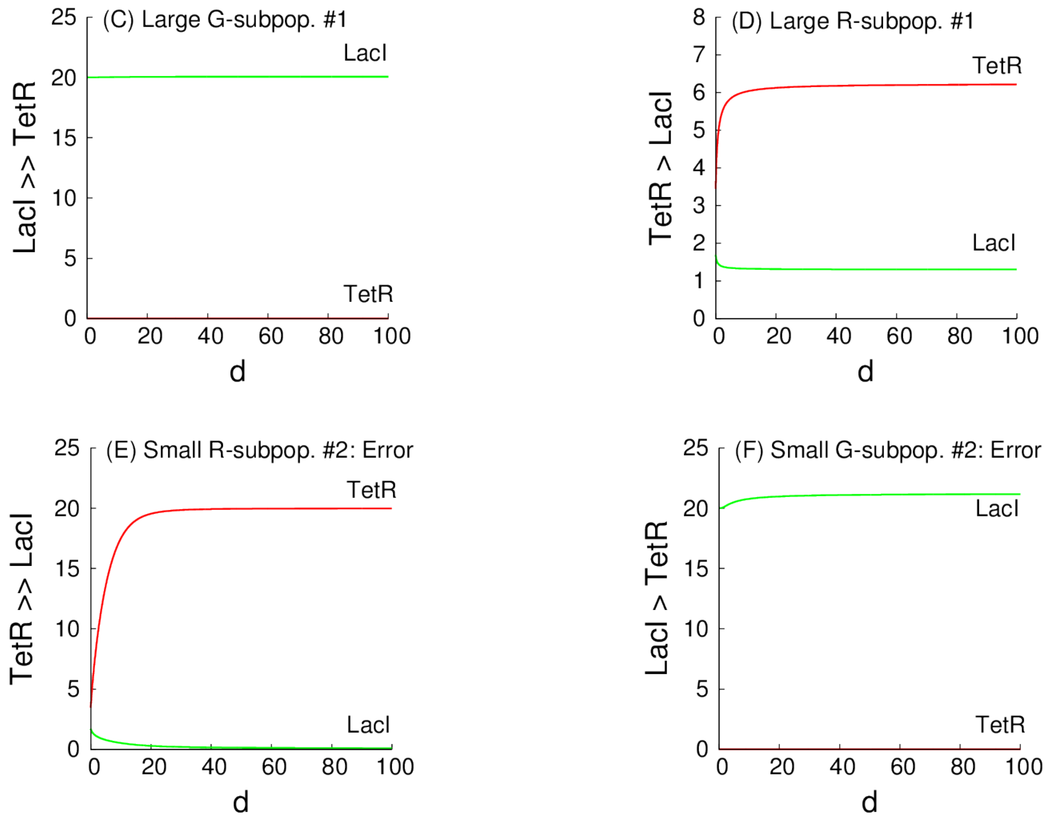

Figure SI-7.8. A (9:1)- and (1:9)-mixed population of A toggles. Here, all notations and color-coding schemes are as in Fig. 9. Panels (A) and (B) correspond to the (9:1)-mixed population, within which the transcription signature LacI $\gg$ TetR dominates in proportion 9:1 (*i.e.*, with 90% of green cells and 10% of red cells), while panels (C) and (D) correspond to the (1:9)-mixed population, within which the opposite transcription signature TetR $\gg$ LacI as well dominates in proportion 9:1 (*i.e.*, with 90% of red cells and 10% of green cells.)

Figure SI-7.9

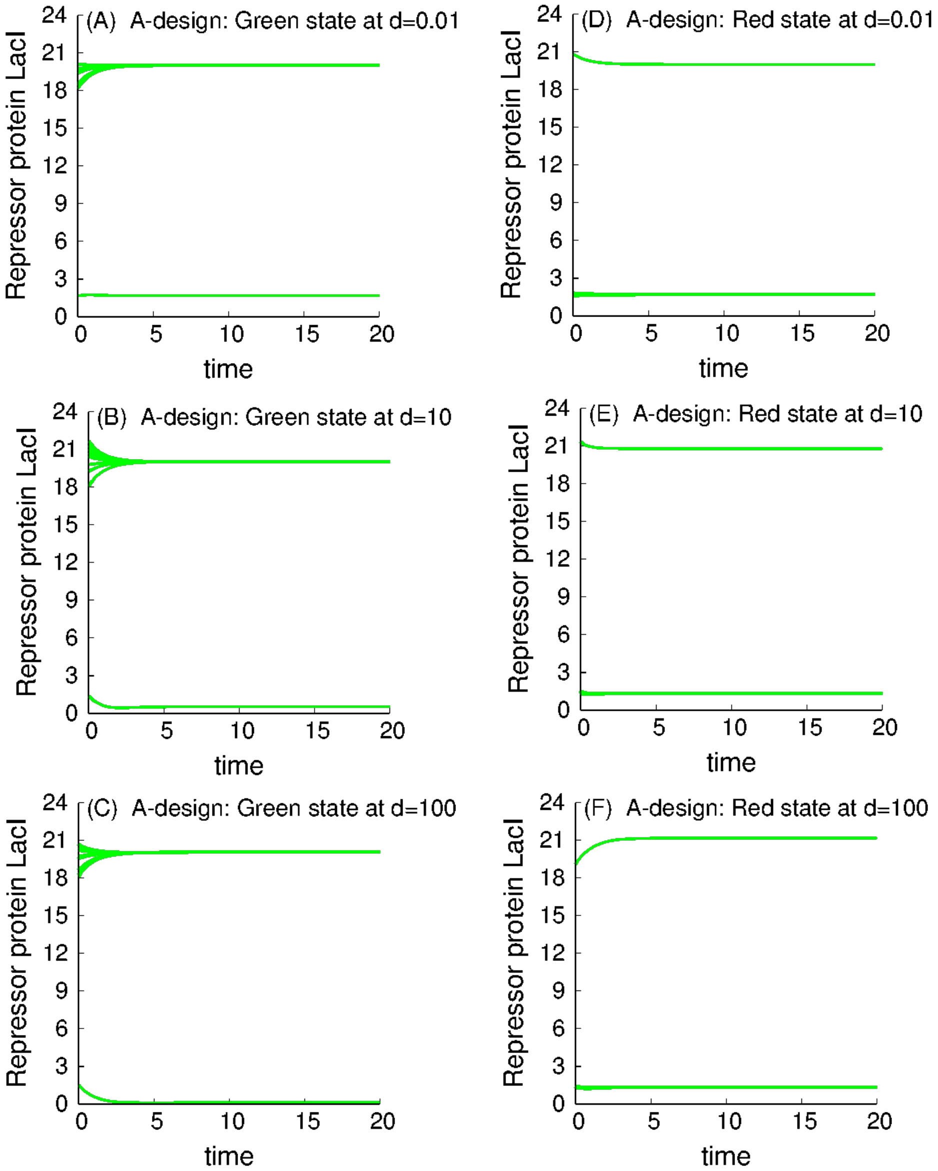

Figure SI-7.9. Lack of any self-correction capability for spontaneous errors by A toggles. The left panels correspond to the reference (10:0)-state (G-state), while the right panels correspond to the reference (0:10)-state (R-state). The initial conditions in the left panels correspond to nine "green" cells and one "red" cell. The initial conditions in the right panels correspond to one "green" cell and nine "red" cells.

SI-8 Modification of the S and A Models to Describe Sequestration of AAA+ protease ClpXP

To probe competition and sequestration effects for AAA+ proteases ClpXP in the context of our monotone theory-based modeling studies described in the main text, we have modified S and A models by adding the corresponding Michaelis-Menten type degradation [37]. Because all scaling procedures used for this case are similar to the scaling procedures described in detail earlier, we omit laborious technical details.

SI-8.1 Modification of the S Model

A dimensionless modified S_m model is

$$\frac{dx_i}{dt} = \gamma_x + \frac{a_1}{1+y_i^{n_Y}} + \frac{a_3\, g_i^{n_G}}{1+g_i^{n_G}} - \delta\, x_i - \frac{k_{ssrA}\, x_i}{1+\ Z_i}, \quad \text{(SI-8.1a)}$$

$$\frac{dy_i}{dt} = \gamma_y + \frac{a_2}{1+x_i^{n_X}} + \frac{a_4 r_i^{n_R}}{1+r_i^{n_R}} - \delta\, y_i - \frac{k_{ssrA}\, y_i}{1+\ Z_i}, \quad \text{(SI-8.1b)}$$

$$\frac{dg_i}{dt} = \gamma_g + \frac{a_5}{1+y_i^{n_Y}} + d\Big(g_e - g_i\Big) - \delta_g\, g_i, \quad \text{(SI-8.1c)}$$

$$\frac{dr_i}{dt} = \gamma_r + \frac{a_6}{1+x_i^{n_X}} + d\Big(r_e - r_i\Big) - \delta_r\, r_i, \quad i = 1, \ldots, N, \quad \text{(SI-8.1d)}$$

$$\frac{dg_e}{dt} = \frac{\rho}{N}\sum_{i=1}^{N} d\Big(g_i - g_e\Big) - \delta_e\, g_e, \quad 0 \le \rho \le 1, \quad \text{(SI-8.1e)}$$

$$\frac{dr_e}{dt} = \frac{\rho}{N}\sum_{i=1}^{N} d\Big(r_i - r_e\Big) - \delta_e\, r_e. \quad \text{(SI-8.1f)}$$

Here, all state variables are as defined for the original (non-modified) S model (1), see the main text. New parameters in (SI-8.1b) and (SI-8.1b) include: k_{ssrA}, a maximal degradation rate for ssrA tagged proteins, δ is an intracellular dilution rate due to cell growth. A new term Z_i used in (SI-8.1b) and (SI-8.1b) is

$$Z_i = x_i/K_{MX} + y_i/K_{MY}. \quad \text{(SI-8.2)}$$

(SI-8.2) assumes that the degradation kinetics of all ssrA-tagged proteins via ClpXP is the same and, hence, can be described with the same Michaelis-Menten equation [37]. Parameters, K_{MY} and K_{MY}, used in (SI-8.2) are scaled Michaelis constants. All other parameters in (SI-8.1a) - (SI-8.1f) are as defined for the original (non-modified) S model (1).

SI-8.2 Modification of the A Model

A dimensionless modified A_m model is

$$\frac{dx_i}{dt} = \gamma_x + \frac{a_1}{1 + y_i^{n_Y}} + \frac{a_4\, r_i^{n_R}}{1 + r_i^{n_R}} - \delta\, x_i - \frac{k_{ssrA}\, x_i}{1 + \; Z_i}, \tag{SI-8.3a}$$

$$\frac{dy_i}{dt} = \gamma_y + \frac{a_2}{1 + x_i^{n_X}} - \delta\, y_i - \frac{k_{ssrA}\, y_i}{1 + \; Z_i}, \tag{SI-8.3b}$$

$$\frac{dr_i}{dt} = \gamma_r + \frac{a_6}{1 + x_i^{n_X}} + d\Big(r_e - r_i\Big) - \delta_r\, r_i, \quad i = 1, \ldots, N, \tag{SI-8.3c}$$

$$\frac{dr_e}{dt} = \frac{\rho}{N} \sum_{i=1}^{N} d\Big(r_i - r_e\Big) - \delta_e\, r_e. \tag{SI-8.3d}$$

Here, all state variables and parameters are as defined for the S_m model (SI-8.1).

SI-8.3 Reference parameter values

For the sake of simplicity and as an important extreme situation, we assume that the reference number of AAA+ protease ClpXP molecules per cell is of the same order of magnitude as the reference number of ssrA-tagged protein molecules, *i.e.*, LacI and TetR (SI-2.2 Nondimensionalization). In other words, we assume that the number of AAA+ protease ClpXP molecules per cell is about 40 monomers per cell, that is, ssrA-tagged LacI and TetR should compete for AAA+ protease ClpXP. A set of all dimensionless parameter values used in the modified models can be found in Table SI-8.1, and is computed based on the data obtained from [37].

Table SI-8.1. Dimensionless parameter values used in the computational modeling.

Name	Description of dimensionless parameters	Value
k_{ssrA}	a maximal degradation rate for ssrA tagged proteins	10.0
K_{MX}	a parameter reciprocal to the non-monotonicity degree for LacI	33.0
K_{MY}	a parameter reciprocal to the non-monotonicity degree for TetR	33.0
δ	an intracellular dilution rate due to cell growth	0.7

References

1. Kobayashi H, Kærn M, Araki M, Chung K, Gardner TS, Cantor CR, et al. Programmable cells: interfacing natural and engineered gene networks. Proceedings of the National Academy of Sciences of the United States of America. 2004;101(22):8414–8419.

2. Kuznetsov A, Kærn M, Kopell N. Synchrony in a population of hysteresis-based genetic oscillators. SIAM Journal on Applied Mathematics. 2004;65(2):392–425.

3. Wang J, Zhang J, Yuan Z, Zhou T. Noise-induced switches in network systems of the genetic toggle switch. BMC systems biology. 2007;1(1):50.

4. Gardner TS, Cantor CR, Collins JJ. Construction of a genetic toggle switch in *Escherichia coli*. Nature. 2000;403(6767):339–342.

5. Bolouri H. Computational modeling of gene regulatory networks: a primer. World Scientific; 2008.

6. Sauro HM. Enzyme kinetics for systems biology. Future Skill Software; 2011.

7. Arpino JA, Hancock EJ, Anderson J, Barahona M, Stan GBV, Papachristodoulou A, et al. Tuning the dials of synthetic biology. Microbiology. 2013;159(Pt 7):1236–1253.

8. Elowitz MB, Leibler S. A synthetic oscillatory network of transcriptional regulators. Nature. 2000;403(6767):335–338.

9. Farrell CM, Baker TA, Sauer RT. Altered specificity of a AAA+ protease. Molecular cell. 2007;25(1):161–166.

10. Cornish-Bowden A. Fundamentals of enzyme kinetics. John Wiley & Sons; 2013.

11. Tuttle LM, Salis H, Tomshine J, Kaznessis YN. Model-driven designs of an oscillating gene network. Biophysical journal. 2005;89(6):3873–3883.

12. Garde C, Bjarnsholt T, Givskov M, Jakobsen TH, Hentzer M, Claussen A, et al. Quorum sensing regulation in *Aeromonas hydrophila*. Journal of molecular biology. 2010;396(4):849–857.

13. Neidhardt FC, Ingraham JL, Schaechter M. Physiology of the Bacterial Cell: A Molecular Approach. Sinauer Associates Sunderland, MA; 1990.

14. Domach M, Leung S, Cahn R, Cocks G, Shuler M. Computer model for glucose-limited growth of a single cell of *Escherichia coli* B/r-A. Biotechnology and bioengineering. 2000;67(6):827–840.

15. Shuler M, Leung S, Dick C. A mathematical model for the growth of a single bacterial cell. Annals of the New York Academy of Sciences. 1979;326(1):35–52.

16. Shuler M. Single-cell models: promise and limitations. Journal of biotechnology. 1999;71(1):225–228.

17. Castellanos M, Wilson DB, Shuler ML. A modular minimal cell model: purine and pyrimidine transport and metabolism. Proceedings of the National Academy of Sciences of the United States of America. 2004;101(17):6681–6686.

18. Nikolaev EV, Atlas JC, Shuler ML. Computer models of bacterial cells: from generalized coarsegrained to genome-specific modular models. In: Journal of Physics: Conference Series. vol. 46. IOP Publishing; 2006. p. 322.

19. Kim BG, Shuler M. A structured, segregated model for genetically modified *Escherichia coli* cells and its use for prediction of plasmid stability. Biotechnology and bioengineering. 1990;36(6):581–592.

20. Shu J, Shuler M. Prediction of effects of amino acid supplementation on growth of *E. coli* B/r. Biotechnology and bioengineering. 1991;37(8):708–715.

21. Laffend L, Shuler M. Ribosomal protein limitations in *Escherichia coli* under conditions of high translational activity. Biotechnology and bioengineering. 1994;43(5):388–398.

22. Laffend L, Shuler M. Structured model of genetic control via the *lac* promoter in *Escherichia coli*. Biotechnology and bioengineering. 1994;43(5):399–410.

23. Bailey JE. Mathematical modeling and analysis in biochemical engineering: past accomplishments and future opportunities. Biotechnology progress. 1998;14(1):8–20.

24. Atlas J, Nikolaev E, Browning S, Shuler M. Incorporating genome-wide DNA sequence information into a dynamic whole-cell model of *Escherichia coli*: Application to DNA replication. Systems Biology, IET. 2008;2(5):369–382.

25. Segel IH. Biochemical Calculations: How To Solve Mathematical Problems In General Biochemistry. Wiley; 1976.

26. Bhattacharya BS, Sweby PK, Minihane AM, Jackson KG, Tindall MJ. A mathematical model of the sterol regulatory element binding protein 2 cholesterol biosynthesis pathway. Journal of Theoretical Biology. 2014;p. 150–162.

27. Lodish H, Berk A, Kaiser CA, Krieger M, Bretscher A, Ploegh H, et al. Molecular cell biology. Macmillan; 2012.

28. Bernstein JA, Khodursky AB, Lin PH, Lin-Chao S, Cohen SN. Global analysis of mRNA decay and abundance in Escherichia coli at single-gene resolution using two-color fluorescent DNA microarrays. Proceedings of the National Academy of Sciences. 2002;99(15):9697–9702.

29. Prindle A, Selimkhanov J, Li H, Razinkov I, Tsimring LS, Hasty J. Rapid and tunable post-translational coupling of genetic circuits. Nature. 2014;.

30. Thomas PW, Stone EM, Costello AL, Tierney DL, Fast W. The quorum-quenching lactonase from *Bacillus thuringiensis* is a metalloprotein. Biochemistry. 2005;44(20):7559–7569.

31. Raj M. PCR Amplification, and Sequence Comparison of lacI gene in WT *E. coli* C29 cells and a presumptive *lacI* Knockout *E. coli* C29 cells to Determine the Difference in the Basal Expression Level of *lacZ* in Lac Operon. J Exp Microbiol Immunol. 2004;6:13–19.

32. Semsey S, Jauffred L, Csiszovszki Z, Erdőssy J, Stéger V, Hansen S, et al. The effect of LacI autoregulation on the performance of the lactose utilization system in *Escherichia coli*. Nucleic acids research. 2013;p. gkt351.

33. Ramos JL, Martínez-Bueno M, Molina-Henares AJ, Terán W, Watanabe K, Zhang X, et al. The TetR family of transcriptional repressors. Microbiology and Molecular Biology Reviews. 2005;69(2):326–356.

34. Markiewicz P, Kleina LG, Cruz C, Ehret S, Miller JH. Genetic studies of the lac repressor. XIV. Analysis of 4000 altered *Escherichia coli* lac repressors reveals essential and non-essential residues, as well as" spacers" which do not require a specific sequence. Journal of molecular biology. 1994;240(5):421–433.

35. Daniel R, Rubens JR, Sarpeshkar R, Lu TK. Synthetic analog computation in living cells. Nature. 2013;497(7451):619–623.

36. Carbonell-Ballestero M, Duran-Nebreda S, Montañez R, Solé R, Macía J, Rodríguez-Caso C. A bottom-up characterization of transfer functions for synthetic biology designs: lessons from enzymology. Nucleic acids research. 2014;42(22):14060–14069.

37. Chen Y, Kim JK, Hirning AJ, Josić K, Bennett MR. Emergent genetic oscillations in a synthetic microbial consortium. Science. 2015;349(6251):986–989.

38. Postle K, Nguyen TT, Bertrand KP. Nucleotide sequence of the repressor gene of the TN10 tetracycline resistance determinant. Nucleic acids research. 1984;12(12):4849–4863.

39. Baumeister R, Flache P, Melefors Ö, von Gabain A, Hillen W. Lack of a 5'non-coding region in Tn 1721 encoded *tetR* mRNA is associated with a low efficiency of translation and a short half-life in *Escherichia coil.* Nucleic acids research. 1991;19(17):4595–4600.

40. Wang Y, Tegenfeldt JO, Reisner W, Riehn R, Guan XJ, Guo L, et al. Single-molecule studies of repressor–DNA interactions show long-range interactions. Proceedings of the National Academy of Sciences of the United States of America. 2005;102(28):9796–9801.

41. Pai A, You L. Optimal tuning of bacterial sensing potential. Molecular systems biology. 2009;5(1).

42. Chen CC, Riadi L, Suh SJ, Ohman DE, Ju LK. Degradation and synthesis kinetics of quorum-sensing autoinducer in *Pseudomonas aeruginosa* cultivation. Journal of biotechnology. 2005;117(1):1–10.

43. Flynn JM, Levchenko I, Seidel M, Wickner SH, Sauer RT, Baker TA. Overlapping recognition determinants within the ssrA degradation tag allow modulation of proteolysis. Proceedings of the National Academy of Sciences. 2001;98(19):10584–10589.

44. Selinger DW, Saxena RM, Cheung KJ, Church GM, Rosenow C. Global RNA half-life analysis in *Escherichia coli* reveals positional patterns of transcript degradation. Genome research. 2003;13(2):216–223.

45. Ochsner UA, Reiser J. Autoinducer-mediated regulation of rhamnolipid biosurfactant synthesis in *Pseudomonas aeruginosa.* Proceedings of the National Academy of Sciences. 1995;92(14):6424–6428.

46. Takaya A, Tabuchi F, Tsuchiya H, Isogai E, Yamamoto T. Negative regulation of quorum-sensing systems in *Pseudomonas aeruginosa* by ATP-dependent Lon protease. Journal of bacteriology. 2008;190(12):4181–4188.

47. Brint JM, Ohman DE. Synthesis of multiple exoproducts in *Pseudomonas aeruginosa* is under the control of RhlR-RhlI, another set of regulators in strain PAO1 with homology to the autoinducer-responsive LuxR-LuxI family. Journal of Bacteriology. 1995;177(24):7155–7163.

48. Sontag ED. Monotone and near-monotone biochemical networks. Systems and Synthetic Biology. 2007;1:59–87.

49. Harary F. On the notion of balance of a signed graph. Michigan Mathematical Journal. 1953;2:143?146.

50. Smith H. Monotone dynamical systems: An introduction to the theory of competitive and cooperative systems, Mathematical Surveys and Monographs, vol. 41. Providence, RI: AMS; 1995.

51. Angeli D, Sontag ED. Monotone control systems. IEEE Trans Automat Control. 2003;48(10):1684–1698.

52. Golubitsky M, Schaeffer DG, Stewart I. Singularities and groups in bifurcation theory. vol. 2. Springer New York; 1988.

53. Kuznetsov YA. Elements of applied bifurcation theory. vol. 112. Springer Science & Business Media; 2013.

54. Nikolaev EV. Bifurcations of limit cycles of differential equations admitting an involutive symmetry. Sbornik: Mathematics. 1995;186(4):611.

55. Krupa M. Bifurcations of relative equilibria. SIAM journal on mathematical analysis. 1990;21(6):1453–1486.

56. Sontag ED. Mathematical control theory: deterministic finite dimensional systems. vol. 6. Springer; 2013.

57. Shnol E. The synchronization of oscillators which interact via a medium. Journal of Applied Mathematics and Mechanics. 1987;51(1):9–13.

58. Katriel G. Synchronization of oscillators coupled through an environment. Physica D: Nonlinear Phenomena. 2008;237(22):2933–2944.

59. Hartman P. Ordinary differential equations. Wiley, New York; 1964.

60. Gantmakher FR. The theory of matrices. vol. 131. American Mathematical Soc.; 1959.

61. Vasil'eva AB, Butuzov VF, Kalachev LV. The boundary function method for singular perturbation problems. SIAM; 1995.
